# Analysis of cancer genomes reveals basic features of human aging and its role in cancer development

**DOI:** 10.1038/ncomms12157

**Published:** 2016-08-12

**Authors:** Dmitriy I. Podolskiy, Alexei V. Lobanov, Gregory V. Kryukov, Vadim N. Gladyshev

**Affiliations:** 1 Division of Genetics, Department of Medicine, Brigham and Women's Hospital and Harvard Medical School, Boston, Massachusetts 02115, USA; 2 Broad Institute, Cambridge, Massachusetts 02142, USA

## Abstract

Somatic mutations have long been implicated in aging and disease, but their impact on fitness and function is difficult to assess. Here by analysing human cancer genomes we identify mutational patterns associated with aging. Our analyses suggest that age-associated mutation load and burden double approximately every 8 years, similar to the all-cause mortality doubling time. This analysis further reveals variance in the rate of aging among different human tissues, for example, slightly accelerated aging of the reproductive system. Age-adjusted mutation load and burden correlate with the corresponding cancer incidence and precede it on average by 15 years, pointing to pre-clinical cancer development times. Behaviour of mutation load also exhibits gender differences and late-life reversals, explaining some gender-specific and late-life patterns in cancer incidence rates. Overall, this study characterizes some features of human aging and offers a mechanism for age being a risk factor for the onset of cancer.

## Abstract (web summary)

Somatic mutations are associated with disease, including cancer. Here, the authors analyse cancer genomic data and show that somatic mutations increase with age and that cancer incidence lags 15 years behind this increase, later in life, mutation and cancer incidence are reduced.

## Graphical abstract

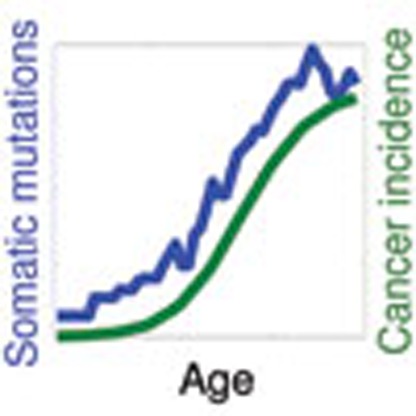

Recent analyses of human whole-genome germline mutations revealed that mutation load in offspring behaves on average as a monotonic function of paternal age, affecting the risk of autism and schizophrenia in the children of older fathers^1^,^2^,^3^,^4^. Growth of somatic mutation load and burden also modulates the risk of disease by increasing the likelihood of mutations directly affecting the relevant genes and perturbing gene regulatory networks. For example, studies suggest that mitochondrial DNA mutations are involved in the development of Alzheimer's and Parkinson's diseases^5^,^6^, and an aging-associated increase in mtDNA mutations would thus lead to an increase in the risk of these neurological diseases with age. Cancers grow from clonal expansions of single mutated somatic cells. Initiation of clonal expansion and transition from a non-malignant to malignant regime are associated with mutations in two to seven gene drivers of cancer^7^, and an age-dependent increase in the overall somatic mutation load naturally leads to a proportional increase in somatic mutation burden, affecting probability of driver mutations and making cancer a disease of aging^8^,^9^.

Although identification and quantitative analysis of age-related somatic mutations are extremely important for understanding the aetiology and estimating the baseline risk of cancer and other diseases of aging, such analysis remains a major technical challenge. Human somatic mutation accumulation rates were previously estimated to be within the range of 70–700 mutations per year in proliferating cells, and an order of magnitude lower in non-replicating cells^10^. Variability in estimated mutation rates highlights many difficulties associated with accurate measurement and quantitative analysis of age-related somatic mutations. In particular, since somatic mutations differ among individual cells of the organism^11^, numerous single cell genomes from subjects of different age are needed to assess the age-dependence of average mutation load and to quantify mutation accumulation rates.

A key feature of somatic mutations in proliferating cells is that they may lead to cancer. It is known that somatic mutation numbers increase with age in many cancers^12^,^13^, consistent with earlier reports of age-related accumulation of mutations in model animals^11^. Genomes of cells in the clone include multiple mutations originating well before the onset of cancer^13^, and clones may be thought of as ‘lenses' amplifying mutational patterns in single cells observed at premalignant stages. Thus, cancer genomes become a powerful tool for characterizing age-related accumulation of human somatic mutations^14^, and an alternative to single cell technologies. Here we show that analyses of cancer genomes allow estimation of the aging-associated increase in mutation load and burden, in turn leading to insights into both aging and cancer.

## Results

### Non-linear increase and slowdown of mutation load with age

**Figure 1:**
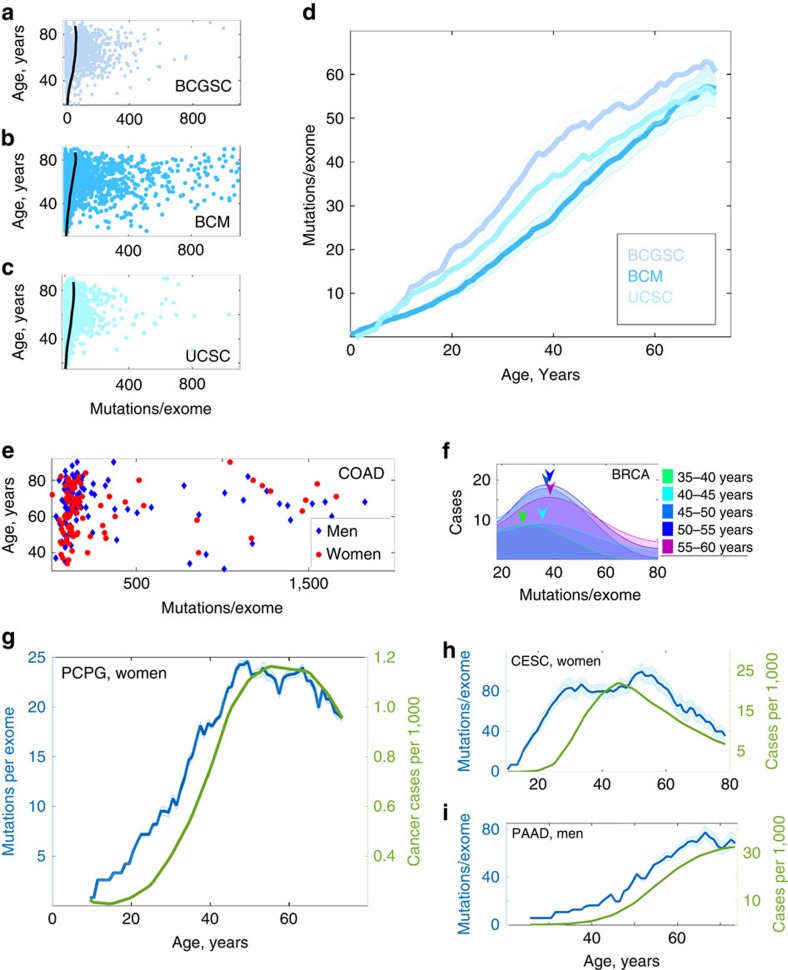
Accumulation of mutations with age and cancer incidence. (**a**) Somatic mutation distribution and combined mutation load for gender-nonspecific cancers (ESCA, LIHC, PAAD, PCPG, SARC, STAD and UVM in both men and women) sequenced by Canada's Michael Smith Genome Sciences Centre (BCGSC). Each point represents a particular cancer sample. Black line denotes behaviour of the median of the distribution with age. (**b**) The same for samples sequenced by Baylor College of Medicine (BCM); cancers include ESCA, LIHC, LGG, PAAD, PCPG, STAD, THCA and UVM. (**c**) The same for samples sequenced by the University of California-Santa Cruz (UCSC); cancers include ESCA, KIRP, LIHC, PAAD, PCPG, SARC and UVM. (**d**) Behaviour of the mutation load distribution medians with age for distributions depicted in **a**–**c**. (**e**) Mutation load distribution for patients with colon adenocarcinoma (COAD), samples sequenced by BCM, IlluminaGA pipeline. Cancers in men are shown in blue, and in women in red. (**f**) Dynamics of the mutation load distribution *P*(*N*, *t*) of somatic mutation counts *N* with age *t* for breast adenocarcinoma (BRCA) in women. Samples sequenced by Washington University, IlluminaGA human-curated pipeline. The actual histograms of mutation loads are approximated by 8 degree polynomials. For each age cohort, the distribution peak is denoted by a coloured arrow. (**g**) Somatic mutation load versus cancer incidence (cases per 1,000 capita) for pheochromocytoma and paraganglioma (PCPG) cancers in women; samples sequenced by Broad Institute, IlluminaGA automated variant-calling pipeline. Cancer incidences (green) and age-related somatic mutation accumulation pattern (blue). (**h**) The same for cervical squamous cell carcinoma and endocervical adenocarcinoma (CESC). Samples sequenced by BCGSC, IlluminaHiSeq automated pipeline. (**i**) The same for pancreatic adenocarcinoma (PAAD) in men. Samples sequenced by BCM, IlluminaGA automated pipeline. Results for other cancers, datacenters and variant-calling pipelines are presented in Supplementary Figs 1–16. Errors are s.d., calculated using bootstrapping as described in Methods.

Taking advantage of the availability of thousands of human cancer genomes in the Cancer Genome Atlas (TCGA)^15^, we quantitatively assessed age-related mutational patterns in cancers. First, we combined different cancer types sequenced by five major research centers (Baylor College of Medicine, Broad Institute, Canada's Michael Smith Genome Sciences Centre, the University of California Santa Cruz and Washington University School of Medicine) and analysed the resulting statistical ensembles of whole-exome samples to assess multi-tissue age-related changes in mutation load. We found that each of the ensembles produced by different sequencing centres was characterized by a non-uniform distribution of cumulative mutation load (Fig. 1a–c), with most cancer samples having <150 mutations/exome and a few samples showing much larger numbers of mutations. As estimated values of somatic mutation load in a given sample depend on the choice of sequencing technology and variant-calling pipelines, and because the lists of cancer types sequenced by individual centres varied, the median mutation loads and their behaviour with age were slightly different among the ensembles of samples produced by different centres. However, in all cases, they were located at relatively low values of 10–150 mutations per exome, and grew with age monotonically but non-linearly, significantly slowing down in late life (Fig. 1d). This non-linear accumulation of somatic mutations with age is also consistent with recent estimations of the behaviour of mutation load with age in hematopoietic clones^16^,^17^.

### Patterns of mutation accumulation in individual tissue types

Analysis of the distributions of mutation loads *P*(*N*, *t*) was then carried out for the data sets representing individual cancer types and segregated according to patient gender, sequencing centre and variant-calling pipeline (Fig. 1e shows a representative example). For every large data set, the distribution *P*(*N*, *t*) possessed a distinct Poisson peak *N*=*N*_full_(*t*) with the position of the peak *N*_full_(*t*) and its width *δN*_full_(*t*) being of the same order. The position of the peak *N*_full_(*t*) grew monotonically with age *t*, then stopped growing in late life (a representative example is in Fig. 1f). The position of the Poisson peak was always located close to the median value of the distribution *P*(*N*, *t*).

Somatic mutation accumulation rates were then extracted from the dynamics of the peak *N*=*N*_full_(*t*), revealing 0.93 mutations per exome per year on average in all tissues and in both genders (Supplementary Tables 1 and 2). Behaviour of the load *N*_full_(*t*) remained distinctly non-linear for all considered tissue types, in particular, the exponential dependence 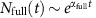 was a significantly better fit then the linear one, *N*_full_(*t*)∼*t*, at early and intermediate ages. This observation can be compared with other studies. An analysis based on tri-nucleotide mutational signatures followed linear mutation growth patterns with age^18^, while another study suggested that the exponential age-dependent growth provides a better fit for the behaviour of the full mutational load with age^19^. It was also found that the linear growth rates do not correlate well among different cancer types^18^. We observed a similar pattern. It can be argued that the linear mutation accumulation rates are not well-defined physically: if the behaviour of mutational load with age is 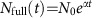, the linear growth rate is given by *N*_0_*α*. The value of *N*_0_ and thus the linear rate itself depend on the choice of variant-calling pipeline used to estimate the mutation load (Supplementary Figs 1–18). On the other hand, we observed (see below) that the mutation accumulation rate doubling time *α*^−1^ does correlate well between cancers.

A noticeable slowdown of mutation accumulation was consistently observed in late life, at 50–80 years, although the age of the beginning of the slowdown varied among cancers (Fig. 1g–i, Supplementary Figs 1–18). For all considered tissue types, the width of the peak *δN*_full_(*t*) remained of the same order as *N*_full_(*t*) at all ages, although the lack of statistical power due to strong heterogeneity of cancers precluded discriminating with certainty between the linear, *δN*_full_(*t*)∼*t*, and the exponential, 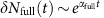, dependences of the load distribution width on age.

### Mutation accumulation rates match all-cause mortality rates

As cancer incidence rates are also known to slowdown in late life^20^,^21^, we compared the behaviour of characteristic age-adjusted mutation loads *N*_full_(*t*) for different cancers with the corresponding US cancer incidence curves (Fig. 1g–i, Supplementary Figs 19–31) obtained from the CDC Wonder Database^22^. We have also compared the patterns with the cancer incidence curves for the UK (obtained from Cancer Research UK^23^) and Australia (obtained from Australian Government^24^) (Fig. 2a–f, Supplementary Figs 32–42). Since for all considered cancer types, the incidence doubling rates and the ages of cancer incidence reaching plateau were the same among the three countries, we focused subsequent analyses on the largest data set that represented US cancer incidence.

It was found that age-adjusted mutation loads and cancer incidences were correlated across all ages. This correlation was particularly strongly expressed in the relation between somatic mutation accumulation and cancer incidence doubling rates. For all considered cancers and tissue types, both age-adjusted mutation loads *N*_full_(*t*) and cancer incidence numbers *N*_incidence_(*t*) grew exponentially with age as 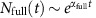 and 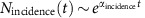 during most of the adulthood. Remarkably, the exponential rates *α*_full_ and *α*_incidence_ were always close to each other and corresponded to the human all-cause mortality doubling rate of 0.125 per year (Fig. 3a,b). In fact, the average of the rates *α*_full_ and *α*_incidence_ among different cancers was within the 1*σ* bound from the human all-cause mortality doubling rate 0.125 per year, and more than half of cancers were within the 2*σ* bound from it, that is, for different cancers mutations accumulate at essentially the same pace, related to the human all-cause mortality doubling rate.

We have also performed a similar analysis of whole genomes for several cancers (Supplementary Figs 43 amd 44). A much smaller number of whole-genome samples compared with the number of available whole exomes has prevented us from identifying the mutation accumulation doubling rates with statistical significance, although we were able to detect mutational load accumulation slowdown at late ages (for example, breast adenocarcinoma and liver hepatic carcinoma (LIHC)). The total characteristic mutational loads in whole genomes exceeded those in whole exomes by 2 orders of magnitude, as should be expected.

### Differences in mutation accumulation among human tissues

By calculating values of *α*_full_ for different cancer types (Supplementary Tables 1 and 2), we estimated the mutation accumulation rates in the corresponding tissues, as well as variability of these rates (Fig. 3a,b). One class of outliers located more than the 2*σ* bound away from the human all-cause mortality doubling rate included reproductive organs represented by gender-specific cancers, such as CESC (cervical squamous cell carcinoma), OV (ovarian serous cystadenocarcinoma), TGCT (testicular germ cell tumours), PRAD (prostate adenocarcinoma), UCEC and UCS (cancers of corpus uteri). Acute myeloid leukaemia (LAML) was also a notable 2*σ* outlier, although the rate *α*_full_ for LAML was close to the average. Another 2*σ* outlier was uveal melanoma, with both *α*_full_ and *α*_incidence_ significantly below the average.

To test whether the observed correlation between the rates *α*_full_ and *α*_incidence_ is not due to the sample selection bias for mutational catalogues, we estimated the distributions of chronological age for different samples represented in TCGA (Supplementary Figs 45 and 46); determined the median chronological ages from these distributions; and calculated the median ages of cancer incidence from incidence curves (Supplementary Tables 3 and 4). For all cancer types represented in TCGA, the median age of cancer incidence was significantly below the median age of patients. On the other hand, the correlation between cancer incidence doubling rate and mutation accumulation rate was observed for early–late mid ages.

### Analysis of silent mutation load

To shed light on the nature of the observed behaviour of the median age-adjusted load *N*_full_(*t*), the same quantitative analysis of cancer genomes was repeated for silent mutations only. A strong linear correlation was found between *α*_full_ and *α*_silent_ (the exponential rate of silent mutation accumulation) among all analysed cancers (Fig. 3c). While the analysis of age-adjusted mutation load *N*_full_(*t*) was largely similar to the analysis of silent mutation load *N*_silent_(*t*), the former had a higher statistical power, since for every tissue type and age the total median mutation load was significantly higher than the median silent mutation count (Fig. 3d,e). The fact that the behaviour of silent mutation counts with age is completely similar to the one of full mutation counts supports the idea that the observed mutation accumulation is the result of a passive stochastic process.

### Mutational patterns most consistently associated with aging

To identify which types of mutations are most strongly accumulated with age, we performed the analysis of mutational patterns in available TCGA samples^12^,^25^,^26^ by applying the method of proper orthogonal decomposition to the TCGA data sets for both developed and early stage cancers (Fig. 3f–h, Supplementary Figs 47–69). A strong prevalence of age-associated CT and GA mutations was found, while the contribution of various indels into the dominating age-correlated mutational signature was generally low (Fig. 3f). The majority of CT and GA mutations occur at CpG sites due to replication errors^27^, corresponding to CpG→TpG and CpG→CpA transitions.

For all considered cancers, the leading age-correlated mutational pattern contributed >50% to the total mutation counts (Fig. 3g), and the projection of mutation counts onto the leading mutational pattern strongly correlated with the characteristic mutation loads *N*_full_(*t*) (Fig. 3h), thus providing an additional method to assess the behaviour of the median age-adjusted mutation load without constructing and analysing full mutation count distributions *P*(*N*, *t*).

It is interesting to note that the mutational signatures 1 and 5 identified in a recent study^18^ as the signatures associated with clock-like mutational processes typically have a contribution to mutational load subdominant to the contribution of signatures associated with extrinsic factors. Thus, they cannot be in the one-to-one correspondence with the leading age-correlated mutational pattern discussed above. If the latter pattern is due to aging, it is expected to have contributions from multiple mutational processes, leading to accumulation of mutations with age. This also explains why the leading pattern is associated with a Poisson-like distribution of mutational loads among different samples. Since it is due to a superposition of the effects of many mutational processes, the distribution of the resulting load is subject to the central limit theorem.

### Time lag between somatic mutation load and cancer incidence

Another feature common to most cancers identified from the comparison of age-adjusted mutation loads *N*_full_(*t*) and cancer incidence curves *N*_incidence_(*t*) was a time delay Δ between the onsets of *N*_full_(*t*) and *N*_incidence_(*t*).

We used several methods to estimate the magnitude of the delay Δ for each cancer. First, the ages of inflection, where the initial exponential regimes of mutation load and incidence growth cease and are followed by slowdown, were calculated for *N*_full_(*t*) and *N*_incidence_(*t*). For all considered cancer types, the incidence inflection and subsequent slowdown were reached 10–20 years later than the same event in mutation accumulation (Fig. 4a). Second, we estimated the time delay between *N*_full_(*t*) and *N*_incidence_(*t*), normalized to the same scale, by minimizing the Euclidean distance functional ∑_*i*_(*N*_full_(*t*_*i*_)−*N*_incidence_(*t*_*i*_))^2^ between the two curves (Fig. 4c,d). Both methods revealed that the cancer incidence lags behind the age-adjusted mutation rate by Δ≈15±10 years (s.d., see Fig. 4e, Table 1), with the lower bound reached by cancers for which early diagnostics methodologies are available (including CESC and breast invasive carcinoma (BRCA)) or slowly developing cancers, such as THCA.

The observed lags between age-dependent cancer incidence and somatic mutation load (Table 1) coincided with known estimations of pre-clinical cancer development times^28^,^29^,^30^,^31^,^32^,^33^, as well as tumour volume doubling times^34^,^35^,^36^,^37^,^38^,^39^,^40^. The latter can be related to cancer pre-clinical development time by evaluating the time required for a clonal expansion initiated from a single cell to reach the size of 10^9^−10^10^ cells, when diagnosis becomes inevitable.

### Gender specificity in somatic mutation accumulation

By examining cancers common to both genders, that is, excluding gender-specific (testicular, prostate, breast, cervical, ovarian and uterine) cancers, we found that the total mutation load was noticeably higher in men than in women, and this pattern was observed for all sequencing centres (Fig. 5a–c). Men also had a higher age-adjusted total cumulative burden of mutations, indicating a higher probability of encountering a damaging mutation at each age (Fig. 5d–f). Analysis of individual common cancers demonstrated that half of the analysed cancer types (10 out of 20, Supplementary Figs 1–22) exhibited a higher age-adjusted mutation load in men than in women, and most of the remaining cancers showed approximately equal mutation loads in men and women.

To further quantitatively assess the gender effects on the landscape of all cancers, we estimated the difference in the mutation load score between men and women (Male–Female, denoted further as ‘MF score'). The MF scores of incidence and mutation load were defined as integrals of cancer incidence curves and age-adjusted mutation load curves over the accessible interval of ages of patients, with subsequent subtraction of the result of integration for women from the result of integration for men (Fig. 5g,h). This analysis showed that a higher overall mutation load in men characterized cancers with an overall higher incidence in men and *vice versa*. For example, analysis of BRCA showed both a higher mutation load and an earlier inflection in women than in men (Fig. 5i), consistent with the known higher incidence rate for breast cancer in women. In the case of HNSC, the mutation accumulation load and late life slowdown in mutation accumulation were similar in men and women (Fig. 5j), whereas STAD showed a higher mutation accumulation in men than in women (Fig. 5k), which again agreed with the incidence rates.

## Discussion

The results of both statistical analysis of somatic mutation load/burden distributions and age-dependent mutational patterns in various cancers suggest that the age-dependent behaviour of median mutation load and burden has the origin common to different cancers. We suggest that the growth of mutation load and burden, as well as behaviour of the identified dominant age-dependent mutational signatures are due to the progressive decrease in fitness with age, that is, the process of aging itself. This possibility is supported by several lines of evidence.

First, as somatic mutations are identified by comparing tumour and control sequences from the same patient, there exists a significant bias towards detection of mutations, which occur during the early stages of clonal expansion, including mutations that occur well before cancer initiation^13^. Similarly, as cancers are typically very inhomogeneous^12^,^41^,^42^,^43^,^44^,^45^, mutations common to all subclones of the sequenced tumour will be most significantly enriched in the final mutation count. This again includes mutations originating prior to initiation of the clonal expansion, leading to cancer. Thus, careful analysis of mutation load and burden distributions in cancer samples allows one to estimate behaviour of the median mutation (non-cancer) load and burden with age in a normal tissue.

Second, as age-related accumulation of somatic mutations in normal tissues is a stochastic passive point-like process, the corresponding distributions of mutation load and burden should be expected to be Poisson-like, unlike distributions of somatic mutations in developed cancers. The latter are strongly influenced by the effects of positive selection^46^, leading to the distributions of somatic mutation loads in the corresponding samples acquiring heavy non-Poisson tails. We have found that the peaks *N*=*N*_full_(*t*) of mutation load and burden distributions are essentially Poisson-like, pointing towards the passive nature of mutation accumulation processes contributing to the peaks of the distributions *P*(*N*_full_, *t*). This is further confirmed by the analysis of distributions of somatic silent mutation load in different cancer types. Behaviour of such distributions with age is found to be similar to the behaviour of the *N*_full_ distributions.

Third and most importantly, mutation load and burden doubling times for the 30 considered cancer types coincide by the order of magnitude with the human all-cause mortality doubling time (Fig. 3a,b). Mutational clocks run with the same pace in different cancer types despite differences in physiology of those cancers, and despite them being characterized by different development time scales and supported by mutations in different drivers. The human all-cause mortality rate doubling time is a known universal characteristic quantifying human morbidity and the accumulation of molecular damage during the process of aging^47^: it is well-known that the incidence of diseases of aging follow the Gompertz mortality curve, parameterized in turn by the human mortality rate doubling time. An age-related increase in the overall mutation burden, proportional to the probability of deleterious mutations, is also a proxy of accumulation of molecular damage in cells. Detected variability in mutation load doubling times among considered cancers thus suggests differences in morbidity increase rates and the rates of aging among different human tissues. In particular, we find a faster (by ∼20%) aging of the human reproductive system, consistent with the reduction in fertility in humans. We also observed a noticeably slower aging of the uvea.

In this respect, we should emphasize that the genome of the most recent ancestor cell of a tumour will also contain a number of mutations realized after the cancer initiation, and our estimates of the characteristic mutational load should only be considered as a lower limit on this quantity. Thus, delays between onsets of mutational load and cancer incidence estimated here are also lower limits, and the actual delays should be somewhat higher than those found by the method outlined in this study.

For all 30 studied cancer types, the median mutation load and burden in different tissues were found to be Granger-causal^48^ to the cancer incidence in the same tissues, preceding the latter by 15±10 years (s.d., Fig. 4a–d). As continuous accumulation of somatic mutations leads to a proportional increase in mutation burden, probability of cancer driver mutations and transformation of clonal expansions to tumours, we interpret the observed delays in cancer incidence versus somatic mutation accumulation as pre-clinical development time scales for cancers. For many cancers (among the 30 cancers discussed here), such time scales were previously unknown. The Granger causality between the median mutation load and cancer incidence also naturally extends the celebrated argument of Armitage and Doll^49^, explaining why the cancer incidence doubling rates are the same among different cancer types.

It is important to note that the hard causality was not established in this analysis, as other factors influenced by aging, for example, epimutations, immune system dysregulation and stem cell niche depletion, were not factored in. However, similarity between the doubling rates of mutational load accumulation, burden accumulation and cancer incidence, as well as Granger-causality relation between mutational load, and cancer incidence do suggest that the accumulation of mutational load with age is a component of cumulative damage and therefore is one of the many factors behind the age-dependent growth of cancer incidence. What leads to the growth of mutational load with age is a separate important question, and here we argue that it is the process of aging itself. One would expect the latter to lead to systematic dysregulation of various functional subsystems of an organism, for example, immune system and epigenetic dysregulation, stem cell niche depletion and so on, on the very same characteristic time scale. In a sense, somatic mutations are just a particular representation of molecular damage, the deleteriome, accumulated in an aging organism^50^.

Finally, our analysis showed that the total somatic mutation load is generally higher in men than in women, which is in good agreement with total cancer incidences in men and women. While the difference in age-adjusted cancer incidence between men and women is well-known^51^,^52^, its molecular explanation was previously lacking. It is tempting to hypothesize that such difference is largely due to differences in mutation accumulation patterns between men and women and, ultimately, differences in the rates of aging between two genders. Interestingly, the total estimated mutation load in men exceeded that in women mostly because somatic mutations started to accumulate earlier by approximately a decade in men than in women, rather than due to faster accumulation rates in men—somatic mutation accumulation rates in men and women were approximately the same for most cancers (Supplementary Tables 1 and 2). This fits well with the behaviour of human Gompertz all-cause mortality curves: while all-cause mortality rate doubling times are similar for men and women, male mortality seems to increase faster in early life than female mortality.

Since cancer is a disease of aging^8^,^9^, age-related changes in mutation accumulation also expose patterns of damage accumulation in cells^53^. An increase in average somatic mutation burden was consistent with an increase in cumulative damage, leading to an exponential increase in mortality as expressed by the Gompertz law. Mortality rate decelerates and reaches plateau in very old humans and laboratory animals^54^ approximately at the age of an average lifespan, which again is consistent with the observed deceleration of the aging process in late life, presumably due to population heterogeneity and other factors. Thus, we suggest that the decline in the rate of aging observed at the population level explains deceleration in the somatic mutation rate in late life, and therefore, reduction in cancer incidence and mortality.

Overall, a cancer genome-derived quantitative assessment of somatic mutations has direct implications for understanding the aging process, causal relationships between aging, accumulation of somatic mutations and the incidence of cancer, and evaluation of the risk for the diseases of aging. The patterns of growth of somatic mutation load and burden with age characterize the rates of aging in different tissues and different individuals, expose gender effects and offer insights into deceleration of aging, mortality rate and cancer incidence in late life. Taken together, this analysis provides a quantitative validation for age being a cancer risk factor.

## Methods

### Collecting data and estimating somatic mutation load

Human whole exomes available from TCGA^15^ corresponding to 30 different cancer types were analysed. For every available cancer type, whole-exome samples were segregated according to the sequencing centre which produced the sample, used variant-calling pipeline, and age and gender of the patient. Somatic mutation numbers were directly extracted from the MAF files (Level 2 data of TCGA) corresponding to each patient. For the whole-genome study, the same full genome data for BRCA, chronic lymphocytic leukaemia, liver hepatic carcinoma (LIHC), B-cell lymphoma, medulloblastoma and pilocytic astrocytoma were used as in refs 12, 18.

For every cancer type and every available whole-exome/genome sample, the total number of somatic mutations *N* was calculated. The gender and age data of patients were collected from the corresponding clinical data files available in TCGA. For whole genomes, age data were available from ref. 18. The distribution function *P*(*N*, *t*) of somatic mutation loads in different age-stratified cohorts was then constructed. The appropriately normalized distributions *P*(*N*, *t*) can be interpreted as probabilities to find a number *N* of somatic mutations per exome/genome in a sample from a patient with age *t*.

The distributions *P*(*N*, *t*) have the following properties:

- The distributions *P*(*N*, *t*) significantly differ from the normal distribution 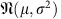 even under the assumption of time dependence of the mean *μ* and the s.d. *σ*; in particular, distributions *P*(*N*, *t*) are skewed,
- For different cancer types, the distributions *P*(*N*, *t*) typically possess a single distinct peak at a relatively low somatic mutation count 10<*N*(*t*)<300 for exomes and *N*(*t*) of the order of a few thousands for genomes,
- Behaviour of the distribution *P*(*N*, *t*) in the vicinity of the peak at *N*=*N*_full_(*t*) is distinctly Poisson-like in the sense that the width of the peak is of the same order as the value of *N* at the peak,
- The position *N*_full_(*t*) of the peak depends on the average age *t* in the cohort and slowly grows with *t*, while the value *P*(*N*_full_, *t*) of probability density at the peak decreases with age,
- Away from the Poisson-like peak at *N*=*N*_full_(*t*) the distributions are characterized by distinctly non-Gaussian, non-Poisson heavy tails, representing relatively rare events of hypermutable cancers or mutation accumulation due to non-Poisson processes.

For every cancer type considered, analysis of the data produced by different sequencing centres has often led to a noticeable variability in somatic mutation count numbers (up to 50% difference in somatic load for less represented cancers). The same observation applied to data sets produced using different variant-calling pipelines for the same sequencing centre. To minimize the effects of variability, all available data produced by different sequencing centres, variant-calling pipelines for every available cancer type have been considered.

Statistical significance of mutational load dependence on age was independently estimated by three methods:

1. For every cancer and every age cohort, bootstrapping procedure was performed, which included random draws (with replacement) of five samples out of the available pool and repeating the procedure of estimating the mutational load outlined above. The error bars for characteristic mutation load were then estimated as s.d. of results of bootstrapping from the mean.
2. For every cancer type/sequencing centre/variant-calling pipeline, a generalized linear model was constructed, relating the source mutational count data (GLM predictors) and the constructed characteristic mutational load (GLM response variable). Statistical significance of the identified values of characteristic mutation load was extracted from GLM errors.
3. The errors in determination of Poisson *λ*=*N*_full_(*t*) extrated from the univariate distributions were collected and then the error provided by moving average of *N*_full_(*t*) was straightforwardly estimated.

All three methods provided estimates of statistical errors of *N*_full_(*t*) of the same order. Smallness of error at early ages is explained by a relatively small number of samples available for those ages. A relatively low overall magnitude of error is fully explained by the fact that moving average over the interval of ages >20 years has been taken. As moving averages are essentially the sum of *N*_full_(*t*) in subsequent time points, the overall error is suppressed by central limit theorem.

### Statistical analysis of mutation load distributions

For every individual cancer type, sequenced by a given centre among five represented in TCGA (Baylor College of Medicine, Broad Institute, Washington University School of Medicine, Canada's Michael Smith Genome Sciences Centre and the University of California Santa Cruz), the distributions of somatic mutation load in different patient age cohorts were constructed. For every sample, the total mutation burden was estimated using PolyPhen2 (ref. 55), and the distributions of somatic mutation burden were then constructed for every analysed cancer type. Somatic mutation load and burden typical for a particular age cohort were estimated by locating the position of the Poisson peak of the distribution of somatic mutation load and burden.

Accumulation of mutations in non-malignant/non-cancer tissues is known to be a random point-like stochastic process satisfying the Poisson distribution. Most recent common ancestor cell contains many such mutations, originating prior to cancer initiation. For the latter (and clonal expansion) to happen, a subsequent number of mutations in two to seven genes-drivers of cancer and/or genes–tumour suppressors is required^7^. After such a transition from the malignant to non-malignant regime is initiated, one can no longer generally expect the somatic mutation count number *N* in the cells of the expanding area to follow the Poisson law, as somatic mutation accumulation becomes strongly subjected to positive selection forces, and heavy non-Poisson tails in the distribution *P*(*N*, *t*) should be expected. Every hypermutable cancer is characterized by its own history of mutation accumulation^46^, and the relative fraction of mutations originating prior to cancer initiation in most recent common ancestor cell is relatively low in this case; such cancers represent events on the heavy tails of the distributions *P*(*N*, *t*). These considerations allow one to focus on the behaviour of *N*_full_ corresponding to the Poisson peak of the distribution *P*(*N*, *t*), interpreting it as a characteristic number of accumulated somatic mutations or characteristic somatic mutation load.

In order to determine dynamics of this load with age, the following strategy has been pursued:

We have constructed a univariate fit of the distribution *P*(*N*, *t*) derived from the data to the Poisson distribution; a fit to the univariate Poisson distribution has allowed us to analyse both relatively small (such as ACC) and relatively large (such as BRCA) data sets in a similar manner,

- The Poisson distribution parameter *λ* provided an approximate position of the peak *N*=*N*_full_(*t*) of the distribution *P*(*N*, *t*),
- The function *N*=*N*_full_(*t*) was then subjected to moving average filtering to suppress the effects of noise and statistical fluctuations due to the smallness of the sample size; the window sizes Δ*t*=20, 25, 30, 35 years were chosen; we have found that the result for the moving-averaged *N*=*N*_full_(*t*) depends only very weakly on the window size at Δ*t*>20 years, while a notable degree of stochasticity is present in the filtered *N*=*N*_full_(*t*), if window sizes smaller than 20 years are chosen.
- Since the moving average filtering introduces (**a**) a time shift of 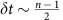 , where *n* is the total number of time points, and (**b**) a bias at ages *t*<Δ*t*−*δt* smaller than the window size minus *δt*, the behaviour of *N*_full_(*t*) at *t*>Δ*t*−*δt* was extrapolated to small ages, see below.

It has been found that the characteristic somatic mutation load *N*_full_(*t*) approximately depends on age according to the law


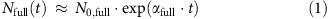


in the interval of ages 20 years<*t*<60 years for most cancer types and exhibits slowdown at later ages (Supplementary Figs 1–18). This dependence was also extrapolated to smaller ages as explained above. The approximate somatic mutation accumulation rate *R*_full_≈*N*_o,full_*α*_full_ was then estimated by constructing the linear least square fit to the mutation count *N*_full_(*t*), while the exponential growth rate *α*_full_ was found by constructing the linear least square fit of the logarithm log(*N*_full_(*t*)) of characteristic mutation load *N*_full_(*t*). Results of these estimations are presented in Supplementary Tables 1 and 2.

An alternative approach for estimation of mutation accumulation rates, which we have pursued, was based on the fact that cancers are only very rarely initiated by silent mutations. For every analysed cancer type and every data sample, we have calculated the distribution functions of silent somatic mutations and followed the steps described above to estimate the characteristic somatic silent mutation number *N*_silent_(*t*), accumulation rates of silent mutations *R*_silent_ and exponential growth rates *α*_silent_ (Supplementary Tables 1 and 2). As discussed in the text, the exponential growth rates *α*_silent_ and *α*_full_ are linearly correlated with each other.

### Estimating lags between mutation load and cancer incidence

For the collected cancer incidence data, it was found that estimated cancer incidence behaved similarly for different cancer types during most of the adult life:


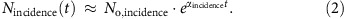


**Figure 2:**
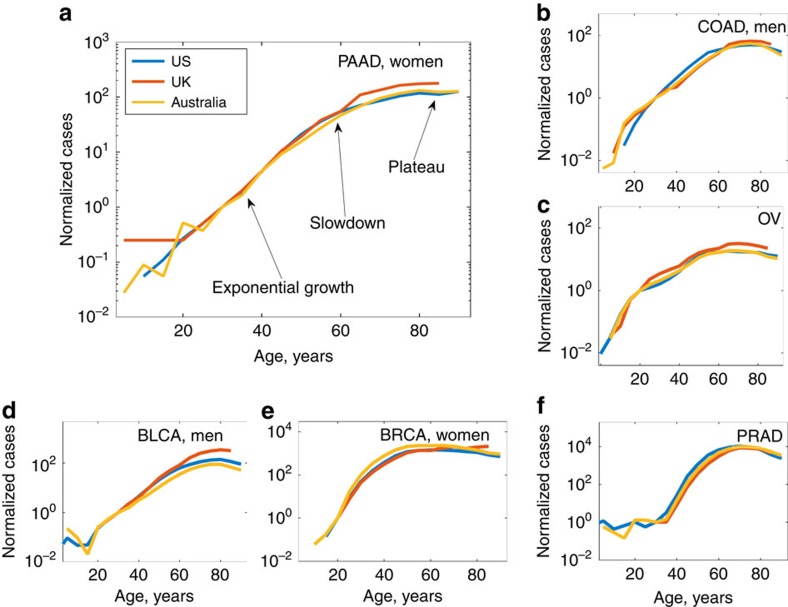
Behaviour of cancer incidence with age. Incidence patterns are normalized for unit incidence at age 30 years and presented in semi-log scale. A typical incidence pattern is characterized by exponential increase at early ages, subsequent slowdown and plateau/decreased incidence in late life. The doubling rates of cancer incidence (linear rates of incidence growth in semi-log scale) and the ages of incidence slowdown/plateau are the same for cancer incidences in all three countries. Standard cancer incidences (cases per 100,000 people) are presented in ‘Growth of cancer incidence with age for different countries' in Supplementary Information. (**a**) Pancreatic cancer in women. (**b**) Colon cancer in men. (**c**) Ovarian cancer. (**d**) Bladder adenocarcinoma in men. (**e**) Breast cancer in women. (**f**) Prostate cancer.

The exponential dependence slowed down reaching plateau (and sometimes decreasing subsequently) in late life for all considered cancer types. We have found that for every cancer type, the magnitude of *α*_incidence_ was essentially the same for US, UK and Australian data (Fig. 2, Supplementary Figs 32–39), and so were the characteristic times of reaching incidence plateau/slowdown. The factors *R*_incidence_ and *α*_incidence_ in equation (2) were estimated using the procedure outlined above.

The lag between cancer incidence curves and *N*_full_(*t*) was estimated using the following two methods:

1. Since both incidence and characteristic somatic mutation loads decreased at late life for all cancers, we estimated the locations of inflection points of incidence curves *N*_incidence_(*t*) and mutation accumulation load curves *N*_full_ and compared them with each other for every cancer type and data set. The inflection point of a curve/function is defined as a point, where its second derivative changes sign: 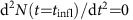 . The delay between the age-related mutation accumulation pattern and cancer incidence can be then estimated as the difference *t*_infl,incidence_−*t*_infl,full_.
2. For every cancer type and data set, we have estimated the Euclidean distance functional between the delayed incidence and characteristic somatic mutation load curves normalized to 1. Such distance (as a function of delay Δ) is defined as the integral

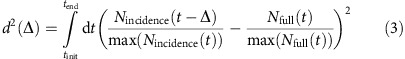


Over the available interval of ages *t*∈[*t*_init_, *t*_end_]. The Euclidean distance *d*^2^(Δ) was then minimized with respect to Δ, and the resulting Δ_min_ (such that *d*^2^(Δ_min_)=min_Δ_ *d*^2^(Δ)) was interpreted as the time lag between mutation accumulation pattern and cancer incidence. The error *δ*Δ in estimation of Δ_min_ was found by solving the equation *d*^2^(Δ_min_±*δ*Δ)≈2*d*^2^(Δ_min_).

### Chronological age distributions for samples covered by TCGA

To make sure that the found lag between the characteristic mutational load and cancer incidence is not due to a possible age-dependence bias present in mutational catalogues, we have constructed chronological age distributions for samples representing individual cancers covered by the TCGA atlas (Supplementary Figs 45 and 46). It was found that the median chronological age of corresponding patients is typically noticeably higher than the median chronological age of cancer incidence for every particular cancer (Supplementary Tables 3 and 4).

### Analysis of mutational patterns

To identify leading age-dependent mutational patterns in TCGA cancer samples, the method of proper orthogonal decomposition was applied. Our approach is different from the one employed in refs 12, 18 in several respects: (i) as we would like to identify the effect of aging on the overall mutation load, we do not need to deconvolve effects of individual mutational processes (many such processes will contribute to the pattern of aging) and thus do not need to perform our analysis in the context of trinucleotides; (ii) since we do not need to identify signatures common to all cancers, instead of pulling all mutational catalogues we independently perform proper orthogonal decomposition for mutational catalogues of individual cancer types (as a result, it is possible to identify differences in age-dependent mutation accumulation for different cancer types/tissues), (iii) while non-negative matrix factorization does not guarantee orthogonality of detected independent components and expansion completeness, singular value decomposition of mutational catalogues does; the identified leading age-dependent component encodes the imprints of all mutational processes characterized by continuous accumulation of mutations with age.

The method included the following steps:

1. For every cancer type and every sample, the numbers of AC, AT, AG, CA, CG, CT, GA, GC, GT, TA, TG, TC SNVs and -A, -C, -T, -G, A-, C-, T-, G-indels were counted; the age of the patient was collected from the clinical data files available in the Cancer Atlas and put to the correspondence to these numbers (20 variables in total, further denoted as *y*_*i*_(*t*), i=1,…, 20),
2. A rectangular 20 × *n* matrix ||*a*_*ij*_||=*y*_*i*_(*t*_*j*_) was constructed for every cancer type, where *n* is the number of available data points,
3. A singular value decomposition of every matrix ||*a*_*ij*_|| was performed (in what follows, we denote singular values of the matrix ||*a*_*ij*_|| as *λ*_(*k*)_ and *a* ^(*k*)^, *b* ^(*k*)^ as corresponding left and right singular vectors),
4. It was explicitly checked that for every matrix ||*a*_ij_|| the largest singular value *λ*_(1)_ dominates over the rest, thus implying the fidelity of the low rank approximation 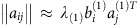 ,
5. The matrix ||*a*_*ij*_|| was projected on its right singular vectors *b* ^(*k*)^ according to the prescription 
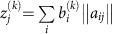
 ; the resulting projections (or ‘modes') 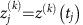 then represented functions of time *z* ^(*k*)^(*t*) denoting behaviour of different accumulation patterns *b* ^(*k*)^ of SNVs and indels with age *t*.

We found that for every considered tissue type the mode *z*^(1)^(*t*), corresponding to the leading singular value *λ*^(1)^ of the matrix, fitted very well with the overall change in the characteristic somatic mutation count *N*_full_(*t*) monotonically increasing with age and slowing down the increase at late ages, while the second dominant mode *z*^(2)^(*t*) together with the rest of the modes oscillate stochastically near 0 (Supplementary Figs 48–69). This observation allowed us to associate the patterns *b*^(1)^ with the process of aging. As discussed in the main text of the paper, for the pattern of SNVs/indels most consistently changing with age in non-malignant tissues is strongly dominated by CT and GA single nucleotide variants.

### Calculating the MF score

The integral MF (‘Male–Female') score of somatic mutation load discussed in the text of the paper was defined according to the following procedure:

1. Integrals


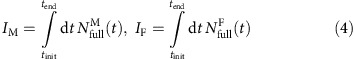


of characteristic somatic mutation loads for men and women were calculated over full accessible intervals of ages.

2. The MF score for mutation load was then defined as 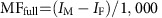. The MF score for incidence was calculated similarly, with a different normalization: 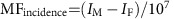.

### Data availability

**Figure 3:**
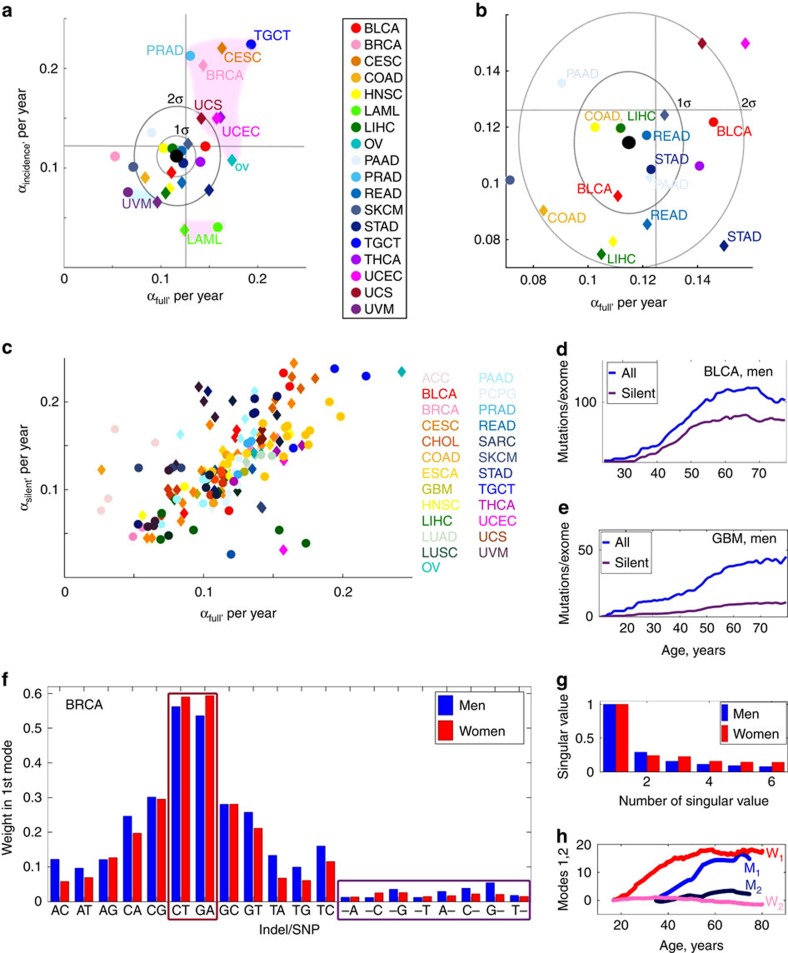
Mutation accumulation doubling rates and mutational patterns. (**a**) Somatic mutation accumulation doubling rate *α*_full_ versus cancer incidence rate doubling rate *α*_incidence_ for different cancer types. Cancers in men are denoted by circles, and cancers in women by diamonds according to the colour scheme on the right. A black spot is an average of rates over all cancers. Grey ovals are the contours of the 1*σ* and 2*σ* deviations from the average. Straight grey lines correspond to the human mortality doubling rate of 0.125 per year. Pinkish clouds correspond to cancers representing reproductive tissues (upper) and leukaemia (lower). The blue cloud represents cancer of the uvea. (**b**) Magnification of **a** showing cancers within the 1*σ*, 2*σ* areas. (**c**) Somatic mutation accumulation doubling rate *α*_full_ versus doubling rate *α*_silent_ for accumulation of silent mutations for different cancer types (gender-specific data combined). Points of the same colour correspond to the data sets representing the same cancer type, sequenced by different datacenters using different variant-calling pipelines. (**d**) Full average counts of somatic mutations per exome versus silent mutations for bladder urothelial carcinoma (BLCA) in men. Samples sequenced by Broad Institute, IlluminaGA pipeline. (**e**) The same for glioblastoma multiforme (GBM) in men. Samples sequenced by Broad Institute, IlluminaGA pipeline. (**f**) Identifying age-dependent mutational signatures for the breast invasive carcinoma (BRCA) data set. Samples sequenced by Washington University School of Medicine, IlluminaGA human-curated pipeline. Relative weights of different mutation types in the first mutational signature. The signature is dominated by CT and GA mutations (dark red rectangle), the contribution of simple indels is subdominant (violet rectangle). (**g**) Relative weights of the first six signatures, normalized to 1; the first signature dominates, corresponding to the pattern of somatic mutation accumulation associated with aging. (**h**) Behaviour of projections onto the first and second leading mutational signatures with age.

**Figure 4:**
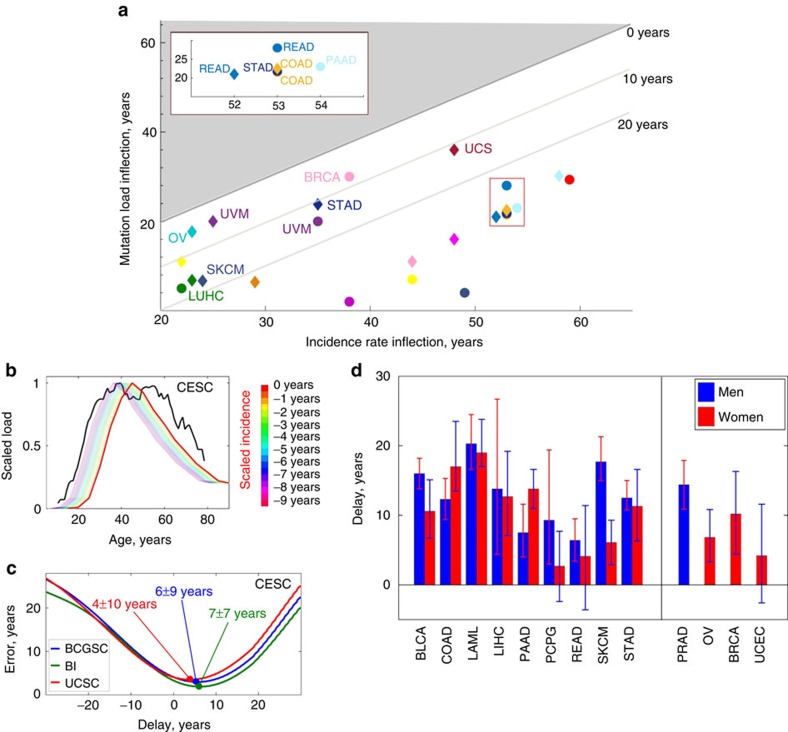
Lags between cancer incidence and mutation accumulation. (**a**) Inflection points in cancer incidence versus inflection points in somatic mutation accumulation. Bounds corresponding to 0, 10 and 20 year delays in cancer incidence versus mutation accumulation are shown, and the select cancers are labelled. The red rectangle is amplified in the inset. The area coloured in grey corresponds to the situation when mutation load inflection occurs later than incidence inflection; no cancers were found to belong to this region. (**b**) Identifying the delay between reported cancer incidence and somatic mutation accumulation for cervical cell carcinoma and endocervical adenocarcinoma (CESC). Samples sequenced by BCGSC, IlluminaGA pipeline. The cancer incidence (red) and somatic mutation accumulation (black) curves are renormalized to the same scale, then the incidence curve is displaced until the Euclidean distance functional between the two curves reaches its minimum. (**c**) Behaviour of time delay estimation error for data sets produced by BCGSC (blue), BI (green) and UCSC (red), cervical cell carcinoma and endocervical adenocarcinoma (CESC). The estimated time delay between cancer incidence and somatic mutation accumulation patterns corresponds to the minimum of the error (denoted by blobs). (**d**) Estimated time delays between cancer incidence and somatic mutation accumulation patterns for non-gender-specific (left) and gender-specific (right) cancers. For every cancer type, averaging over estimates based on the results by different datacenters/pipelines was performed. Red bars correspond to women, and blue to men. Errors are s.d., calculated using bootstrapping as described in Methods.

**Figure 5:**
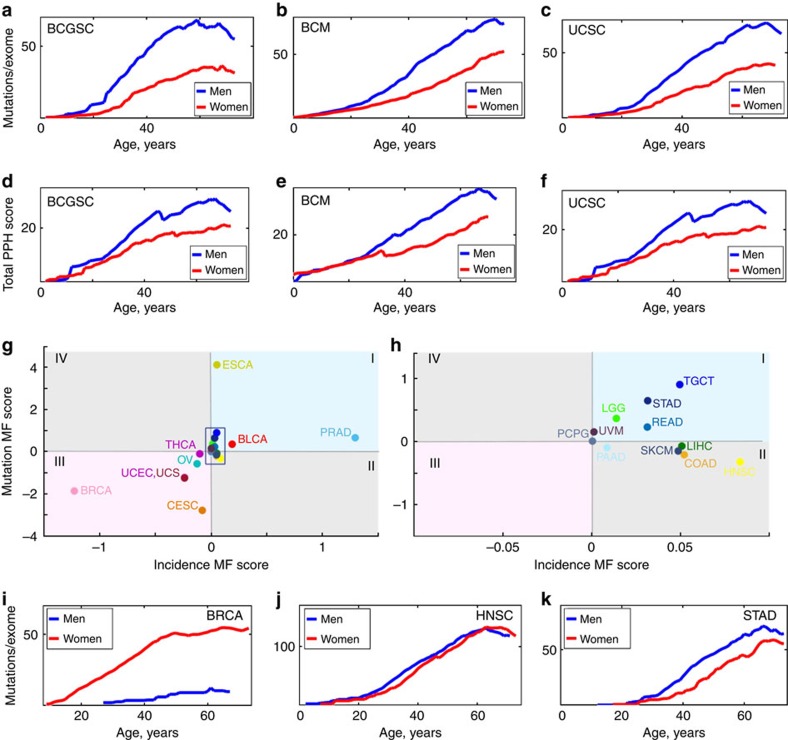
Gender effects in the behaviour of mutation load and burden. (**a**) Total mutation load for combined non-gender-specific cancers (ESCA, LIHC, PAAD, PCPG, SARC, STAD and UVM) sequenced by BCGSC, IlluminaHiSeq automated pipeline. The data in men are shown in blue and in women in red. (**b**) The same for cancers (ESCA, LIHC, LGG, PAAD, PCPG, STAD, THCA and UVM) sequenced by BCM, IlluminaGA automated pipeline. (**c**) The same for cancers (ESCA, KIRP, LIHC, PAAD, PCPG, SARC and UVM) sequenced by UCSC, IlluminaGA automated pipeline. (**d**–**f**) Total mutation burdens for the same combinations of cancers, sequencing centres and pipelines as in **a**–**c**. Values of mutation burden were estimated using PolyPhen2 (ref. 55). (**g**) The MF (Male—Female) scores of mutation accumulation rates versus cancer incidence rates characterizing differences in mutation load between men and women. The blue area on the plots corresponds to cancers with higher overall mutation load in men than in women, and the pink area to cancers with lower overall mutation load in men than in women. (**h**) The area in rectangle in **g** is amplified and cancers indicated. (**i**–**k**) Examples of gender-specific mutation load behaviour of several cancer types. (**i**) Breast invasive carcinoma (BRCA). Samples sequenced by WUSM using IlluminaGA human-curated pipeline. Mutation load in men is significantly lower than mutation load in women for all ages. (**j**) Head and neck squamous cell carcinoma (HNSC). Samples sequenced by BI using IlluminaGA automated pipeline. Mutation load in men and women is approximately the same for all ages. (**k**) Stomach adenocarcinoma (STAD), samples sequenced by BCM using IlluminaGA automated pipeline. Mutation load in men is higher than in women for all ages.

All data used in this study are publicly available. Whole exomes used in the study were obtained from TCGA^15^ (https://tcga-data.nci.nih.gov). Somatic mutation count data for whole genomes analysed in the study can be found in Supplementary Information of ref. 18. Cancer incidence data were collected from 1998–2011 CDC WONDER database of United States Department of Health and Human Services, Centers for Disease Control and Prevention^22^ (US data were used in Figs 1, 3 and 4), Cancer Research UK^23^ and Australian Government^24^. Any other data is available from the authors upon request.

## Additional information

**How to cite this article:** Podolskiy, D. I. *et al*. Analysis of cancer genomes reveals basic features of human aging and its role in cancer development. *Nat. Commun.* 7:12157 doi: 10.1038/ncomms12157 (2016).

## Supplementary Material

Supplementary Information Supplementary Figures 1-69 and Supplementary Tables 1-4.

## Figures and Tables

**Table 1:** Delays of cancer incidence relative to somatic mutation accumulation patterns for different cancer types.

**Cancer**	**Delay (men), years**	**Error estimation (men), years**	**Delay (women), years**	**Error estimation (women), years**	**Pre-clinical development, years**	**Tumor volume doubling time, years**
BLCA	16.0	±2.2	10.6	+4.5		0.35 (ref. 35)
				−3.9		
BRCA	0.0	±5.6	10.2	+6.1	12 (refs 30, 32)	0.4 (refs 30, 32)
				−5.8		
CESC	NA	NA	5.1	±8.8	12 (ref. 31)	0.24 (ref. 35)
COAD	12.3	+3.0	17.0	+6.5	17 (ref. 29)	0.36 (ref. 30)
		−2.9		−3.5		
LAML	20.3	+4.2	19.0	+4.8		
		−3.7		−2.0		
LIHC	13.8	+12.9	12.7	+6.5	6–12 (ref. 30)	0.2–0.3 (ref. 34)
		−9.4		−5.6		
OV	NA	NA	6.8	+4.0	>4 (ref. 28)	
				−3.5		
PAAD	7.5	+4.1	13.8	±2.8	11–17 (ref. 33)	0.18–0.7 (ref. 36)
		−3.5				
PCPG	9.3	+10.1	2.7	+5.0		
		−6.3		−5.1		
PRAD	14.4	±3.5	NA	NA		0.5–2 (ref. 39)
READ	6.4	+3.1	4.1	+7.3	17 (ref. 29)	
		−3.0		−7.7		
SKCM	17.7	+3.6	6.1	±3.2		0.13 (ref. 35)
		−2.7				
STAD	12.5	+2.5	11.3	+5.3		0.17–2.0 (ref. 37)
		−1.7		−5.0		
UCEC	NA	NA	4.2	+7.4		0.24 (ref. 35)
				−6.8		

BLCA, bladder urothelial carcinoma; BRCA, breast invasive carcinoma; CESC, cervical cell carcinoma and endocervical adenocarcinoma; COAD, colon adenocarcinoma; LAML, acute myeloid leukemia; LIHC, liver hepatic carcinoma; NA, not applicable; OV, ovarian serous cystadenocarcinoma; PAAD, pancreatic adenocarcinoma; PCPG, pheochromocytoma and paraganglioma; PRAD, prostate adenocarcinoma; READ, rectum adenocarcinoma; SKCM, skin cutaneous melanoma; STAD, Stomach adenocarcinoma; UCEC, uterine corpus endometrial carcinoma.

Delay times for different data centres/variant-calling pipelines were calculated as described in the text. Results from different data centres and variant-calling pipelines were then averaged for every cancer type. The last column is tumour volume doubling time as defined in ref. 35. Errors are s.d., calculated using bootstrapping as described in Methods.
