# Supplementary material for: Analysis of cancer genomes reveals basic features of human aging and its role in cancer development
Source: Nat Commun. 2016 Aug 12;7:12157. doi: 10.1038/ncomms12157 (PMC4990632; doi:10.1038/ncomms12157)
Supplement: Supplementary Information — Supplementary Figures 1-69 and Supplementary Tables 1-4. [file ncomms12157-s1.pdf]

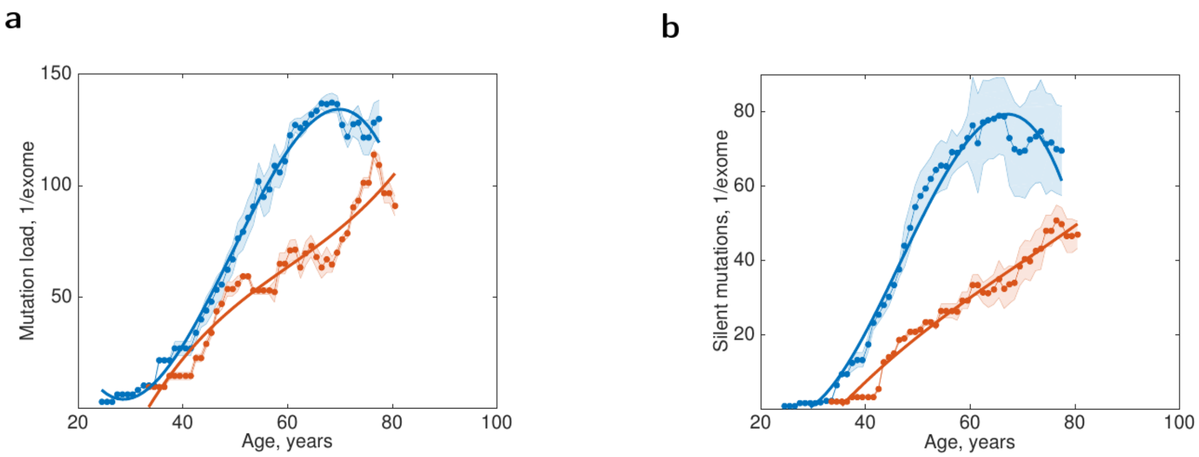

Supplementary Figure 1 | Characteristic somatic mutation load in bladder urothelial carcinoma (BLCA). Men (blue) vs. women (red). **a.** BI, IlluminaGA DNASeq, automated pipeline, all mutations. **b.** BI, IlluminaGA, DNASeq, automated pipeline, silent mutations only.

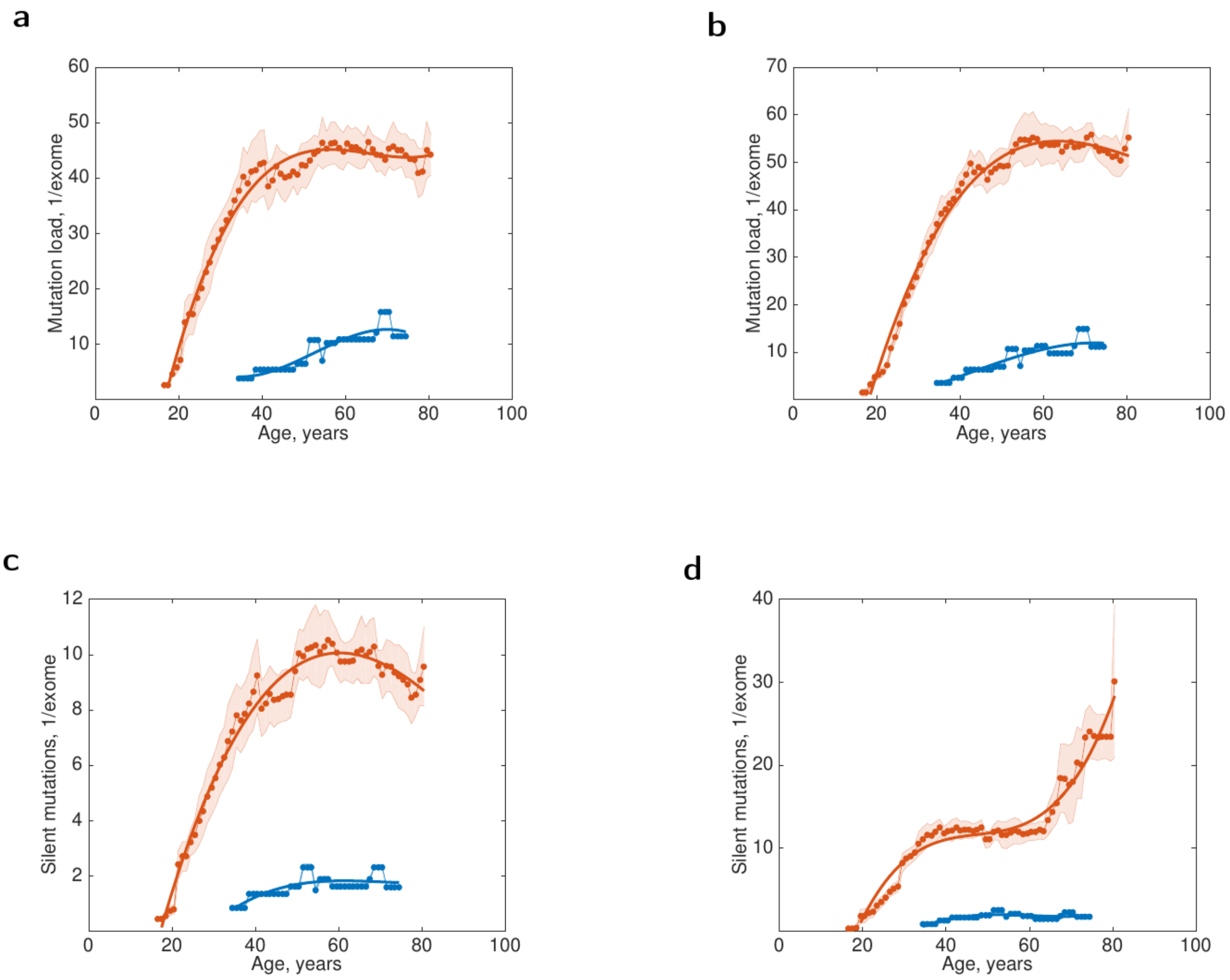

Supplementary Figure 2 | Characteristic somatic mutation load in breast invasive carcinoma (BRCA). Men (blue) vs. women (red). a. WUSM, IlluminaGA DNASeq, automated pipeline, all mutations. **b**. WUSM, IlluminaGA, DNASeq, curated pipeline, all mutations. **c.** WUSM, IlluminaGA DNASeq, silent mutations only. **d.** WUSM, IlluminaGA DNASeq, curated pipeline, silent mutations only.

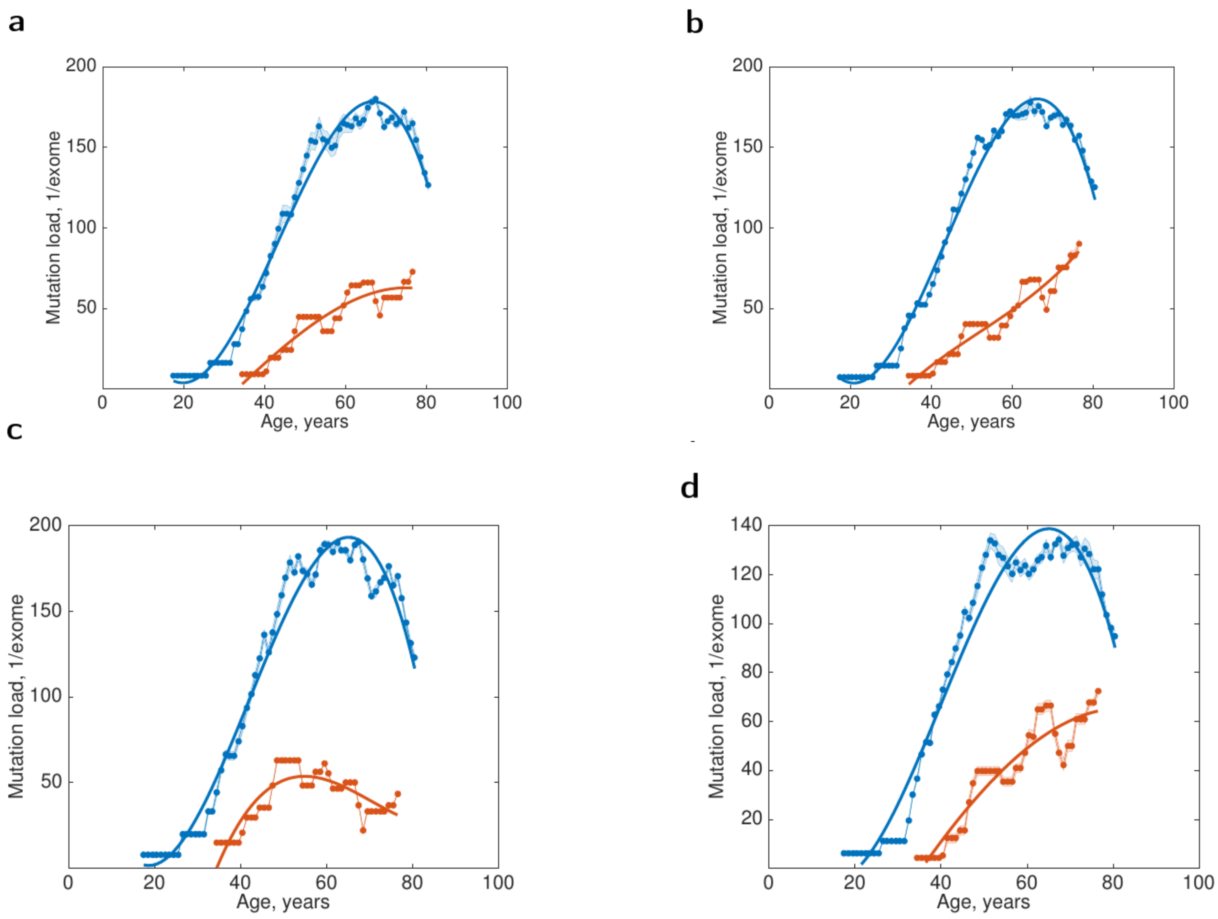

Supplementary Figure 3 | Characteristic somatic mutation load in esophageal carcinoma (ESCA), all mutations. Men (blue) vs. women (red). **a**. BCGSC, IlluminaHISeq DNASeq, automated pipeline. **b**. BI, IlluminaGA, automated pipeline. **c.** BCM, IlluminaGA DNASeq, automated pipeline. **d.** UCSC, IlluminaGA, IlluminaGA DNASeq, automated pipeline.

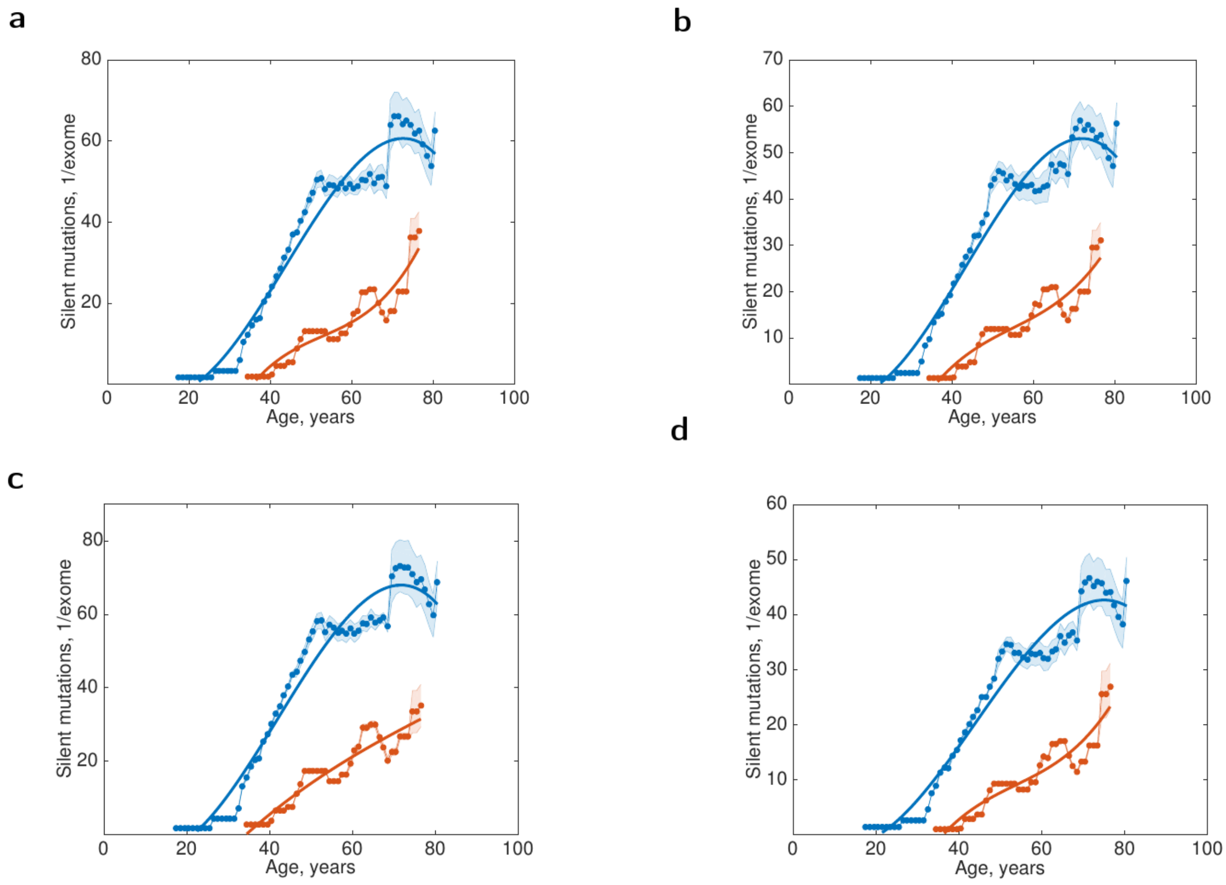

Supplementary Figure 4 | Characteristic somatic mutation load in esophageal carcinoma (ESCA), silent mutations only. Men (blue) vs. women (red). **a.** BSGSC, IlluminaHiSeq DNASeq, automated pipeline. **b.** BI, IlluminaGA, automated pipeline. **c.** BCM, IlluminaGA DNASeq, automated pipeline. **d.** UCSC, IlluminaGA, automated pipeline.

a

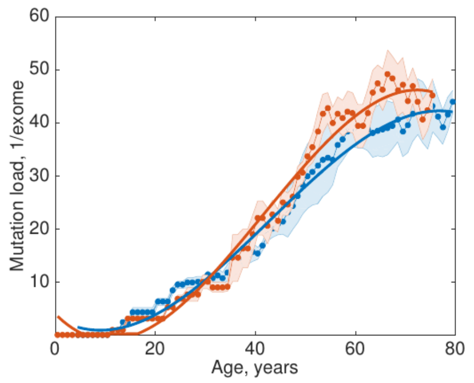

b

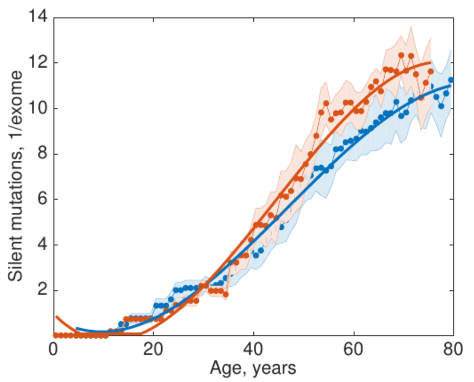

Supplementary Figure 5 | Characteristic somatic mutation load in glioblastoma multiforme (GBM). Men (blue) vs. women (red). **a.** BI, IlluminaGA DNASeq, all mutations. **b.** BI, IlluminaGA DNASeq, silent mutations only.

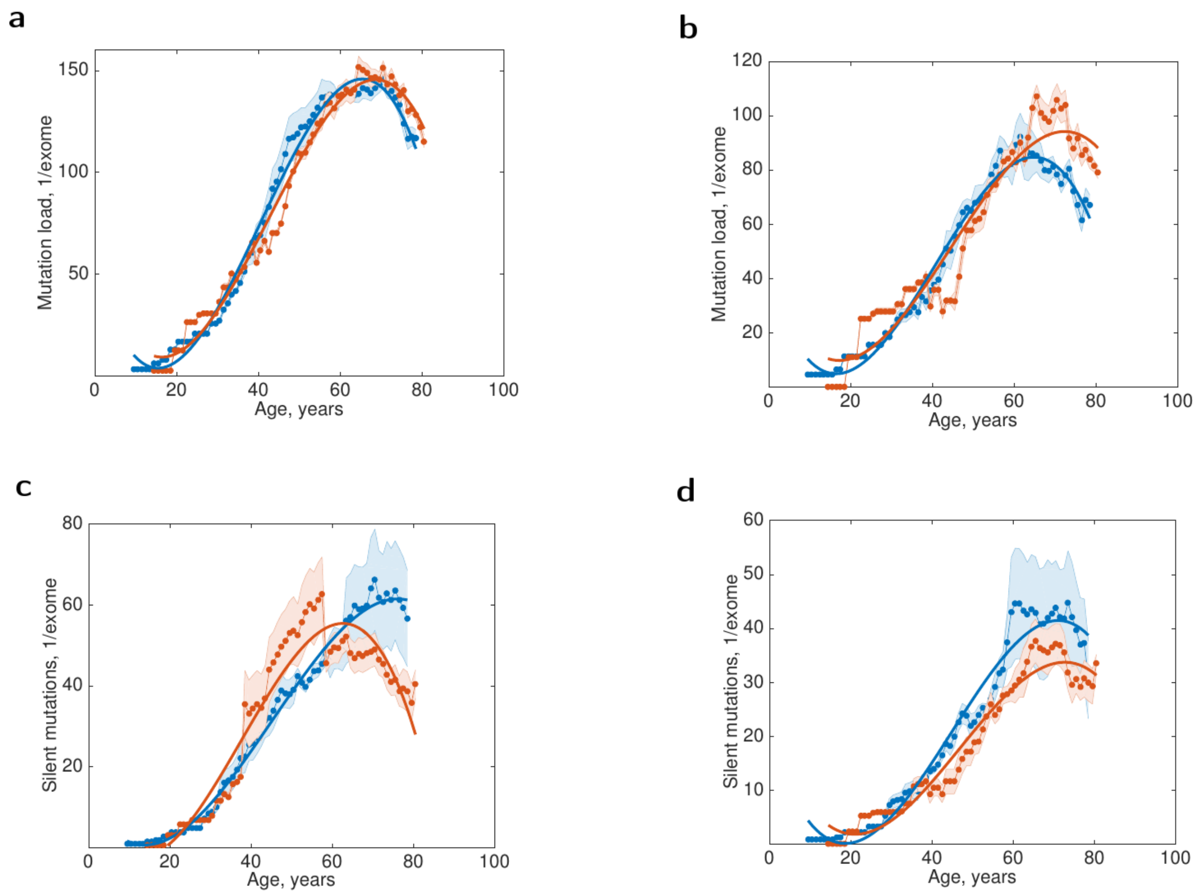

Supplementary Figure 6 | Characteristic somatic mutation load in head and neck squamous cell carcinoma (HNSC). Men (blue) vs. women (red). **a.** BI, IlluminaGA DNASeq, automated pipeline, all mutations. **b.** BI, IlluminaGA DNASeq, all mutations. **c.** BI, IlluminaGA DNASeq, automated pipeline, silent mutations only. **d.** BI, IlluminaGA DNASeq, silent mutations only.

a

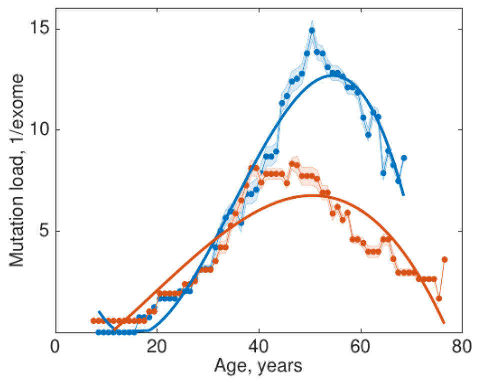

b

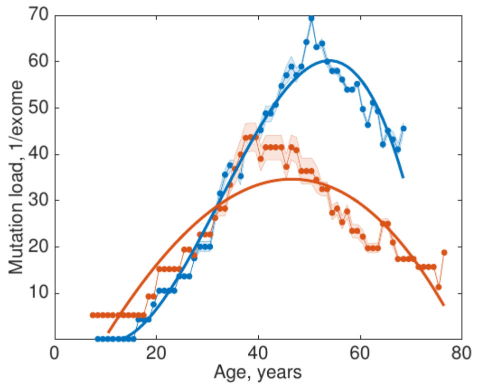

Supplementary Figure 7 | Characteristic somatic mutation load in kidney chromophobe (KICH), all mutations. Men (blue) vs. women (red). **a.** BCM, IlluminaGA DNASeq. **b.** BCM, mixed IlluminaGA DNASeq and SOLiD.

a

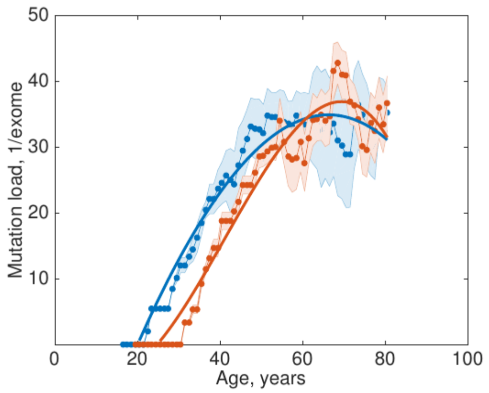

b

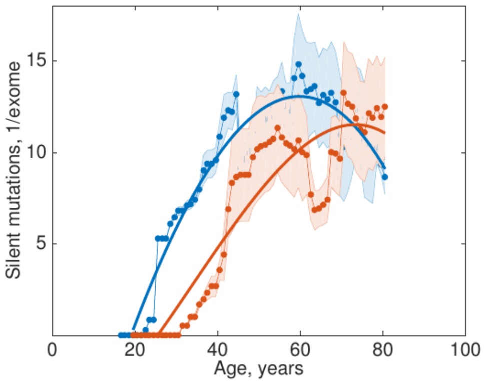

Supplementary Figure 8 | Characteristic somatic mutation load in kidney renal clear cell carcinoma (KIRC). Men (blue) vs. women (red). **a.** BI, IlluminaGA DNASeq, automated pipeline, all mutations. **b.** BCM, mixed IlluminaGA and SOLiD, silent mutations only.

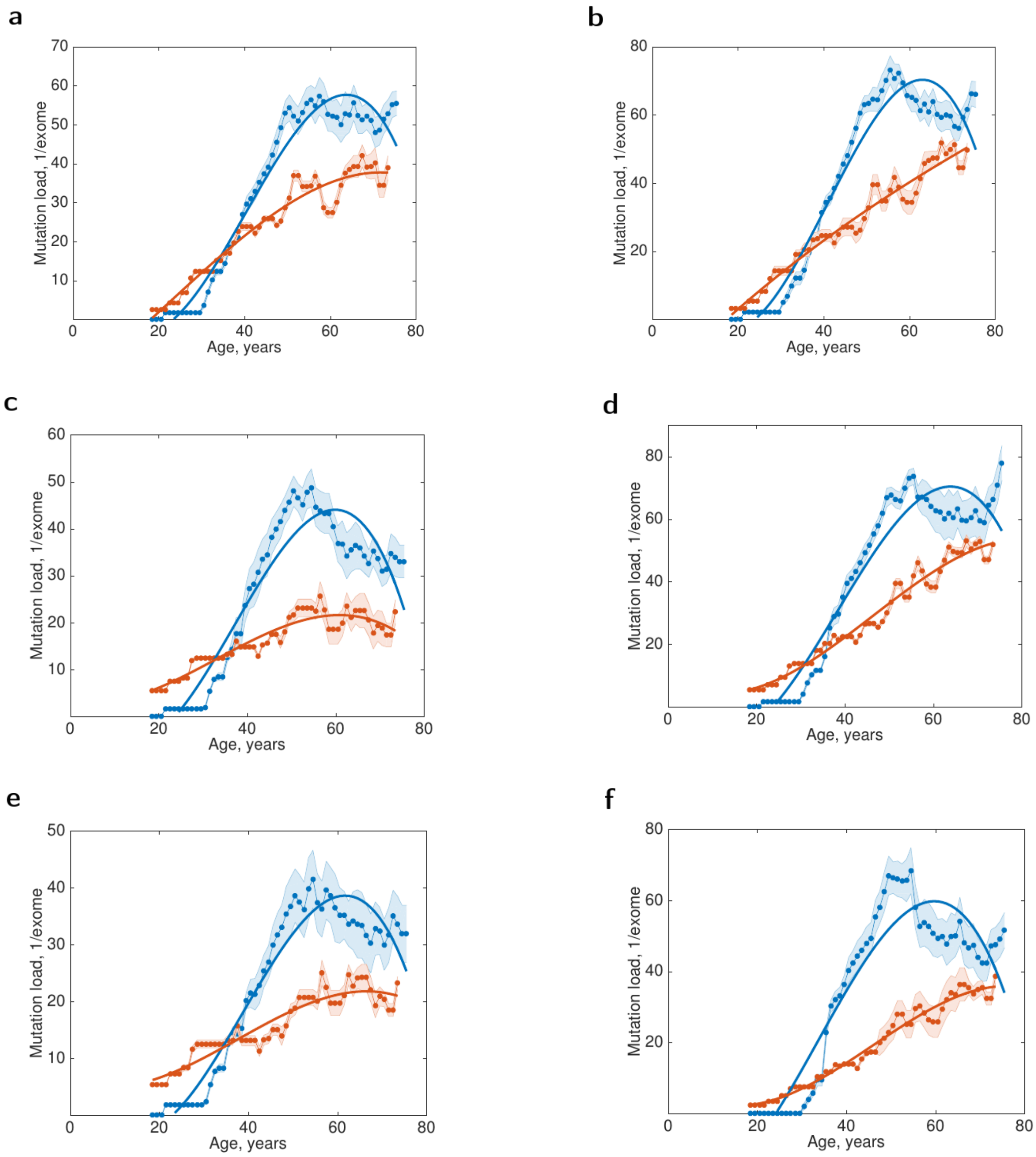

Supplementary Figure 9 | Characteristic somatic mutation load in kidney renal papillary cell carcinoma (KIRP), all mutations. Men (blue) vs. women (red). **a.** BCM, IlluminaGA DNASeq, curated pipeline. **b.** BCM, mixed IlluminaGA and SOLiD. **c.** BI, IlluminaGA DNASeq, automated pipeline. **d.** BI, IlluminaGA DNASeq, curated pipeline. **e.** BI, IlluminaGA DNASeq. **f.** UCSC, IlluminaGA DNASeq, automated pipeline.

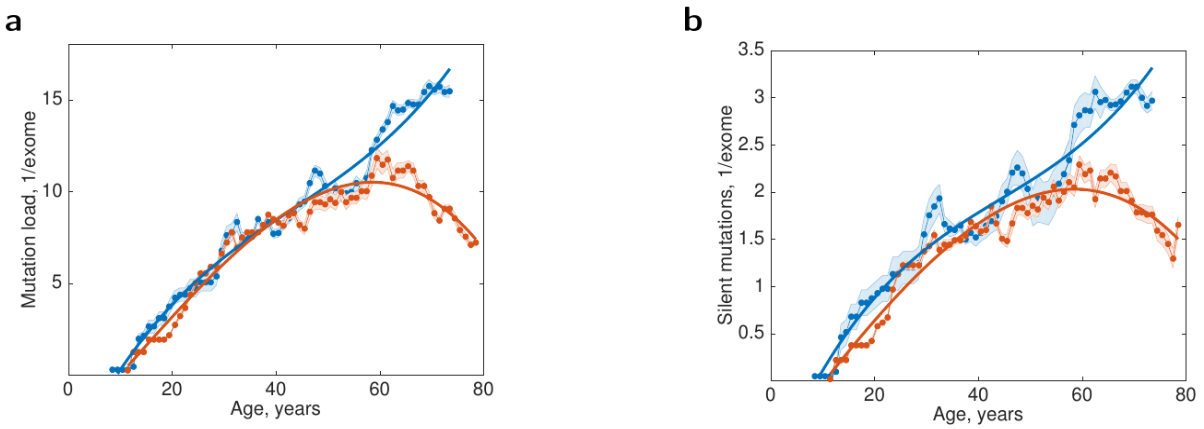

Supplementary Figure 10 | Characteristic somatic mutation load in acute myeloid leukemia (LAML). Men (blue) vs. women (red). **a.** WUSM, IlluminaGA DNASeq, all mutations. **b.** WUSM, IlluminaGA DNASeq, silent mutations only.

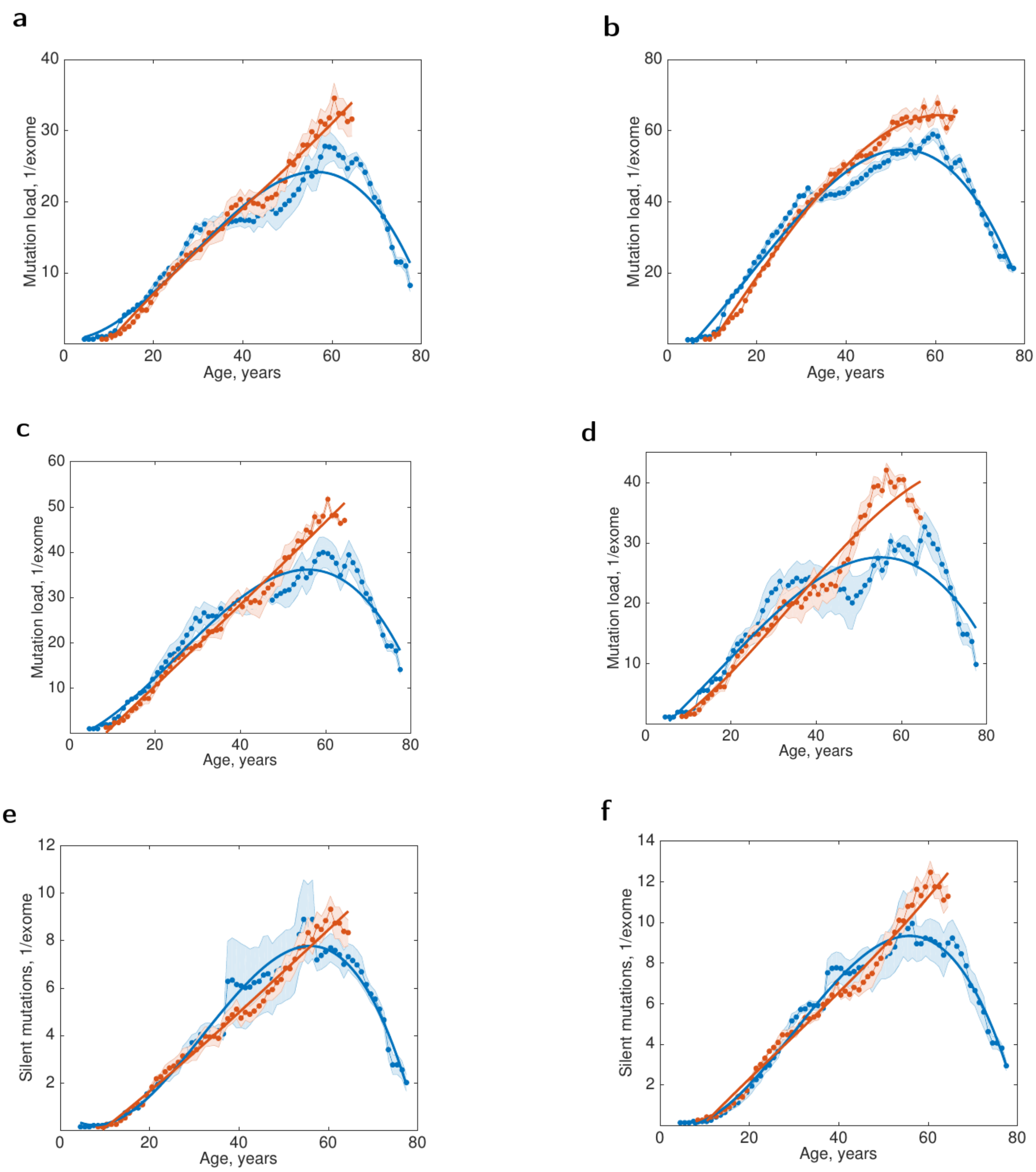

Supplementary Figure 11 | Characteristic somatic mutation load in brain lower grade glioma (LGG). Men (blue) vs. women (red). **a.** BCM, IlluminaGA DNASeq, automated pipeline, all mutations. **b.** BI, IlluminaGA DNASeq, curated pipeline, all mutations. **c.** BI, IlluminaGA DNASeq, all mutations. **d.** BI, IlluminaGA DNASeq, curated pipeline, silent mutations only. **e.** BCM, IlluminaGA DNASeq, automated pipeline, silent mutations only. **f.** BI, IlluminaGA DNASeq, silent mutations only.

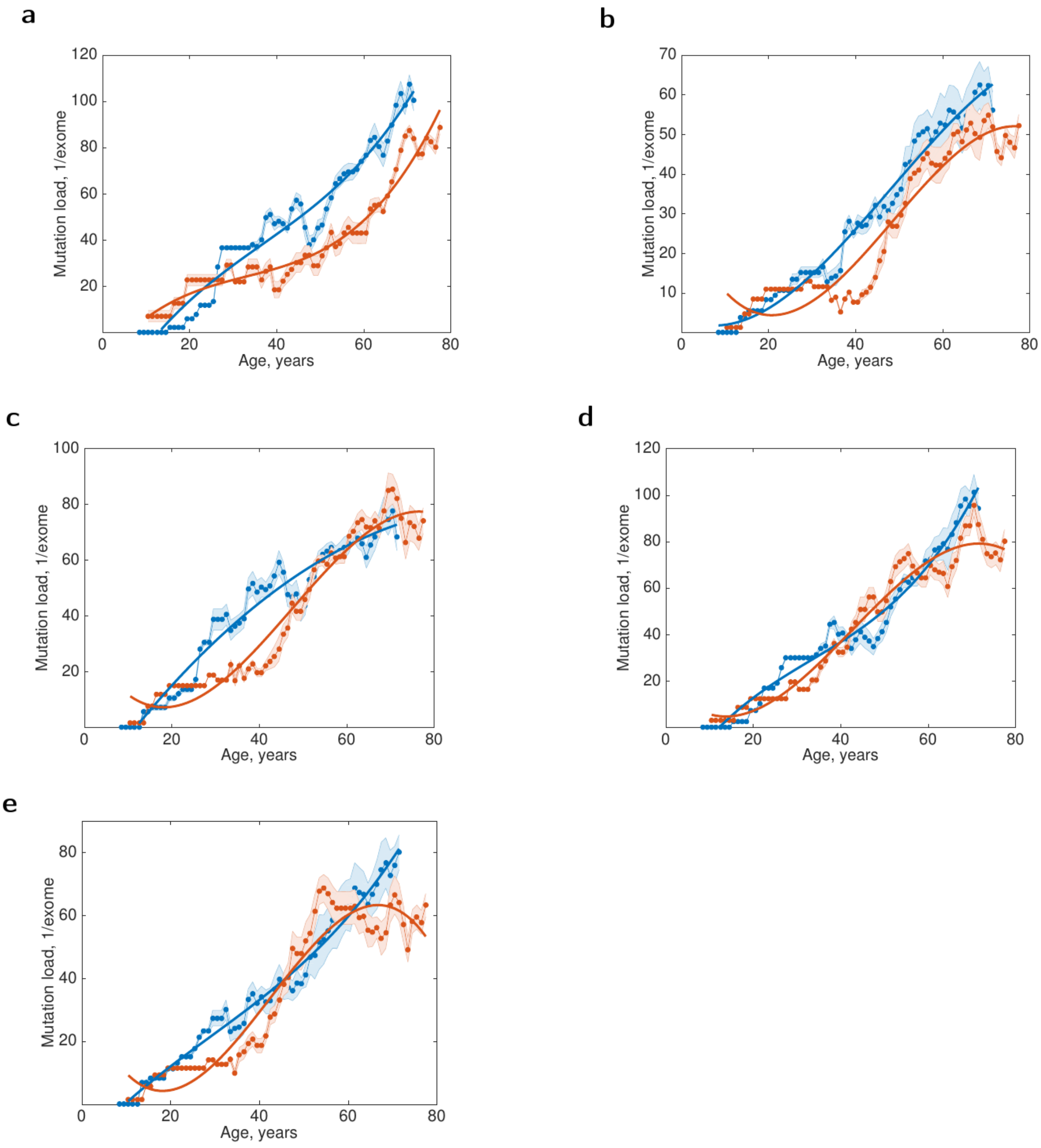

Supplementary Figure 12 | Characteristic somatic mutation load in liver hepatocellular carcinoma (LIHC), all mutations. Men (blue) vs. women (red). **a.** BCGSC, IlluminaHiSeq DNASeq, automated pipeline. **b.** BCM, IlluminaGA DNASeq, automated pipeline. **c.** BCM, mixed IlluminaGA and SOLiD, curated pipeline. **d.** BI, IlluminaGA DNASeq, automated pipeline. **e.** UCSC, IlluminaGA DNASeq, automated pipeline.

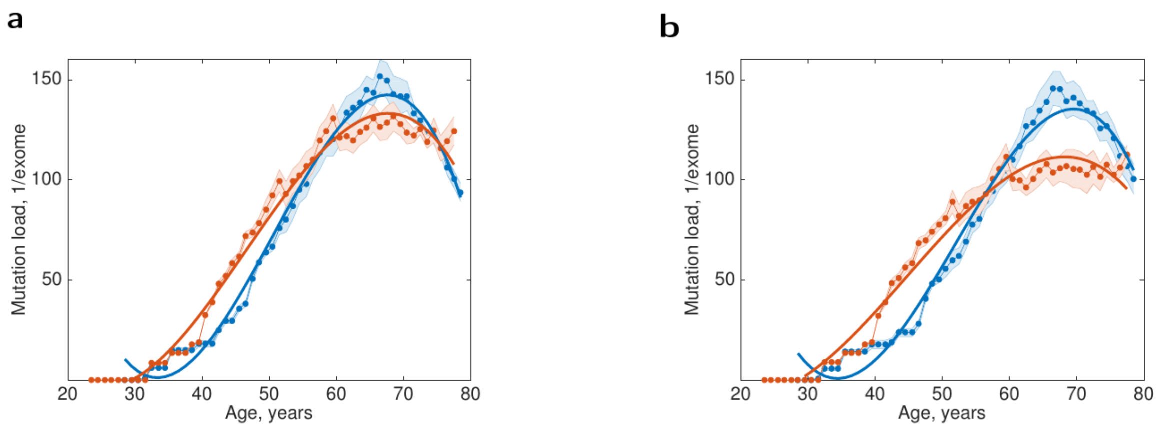

Supplementary Figure 13 | Characteristic somatic mutation load in lung adenocarcinoma (LUAD). Men (blue) vs. women (red). **a.** BI, IlluminaGA DNASeq, automated pipeline, all mutations. **b.** BI, IlluminaGA DNASeq, all mutations.

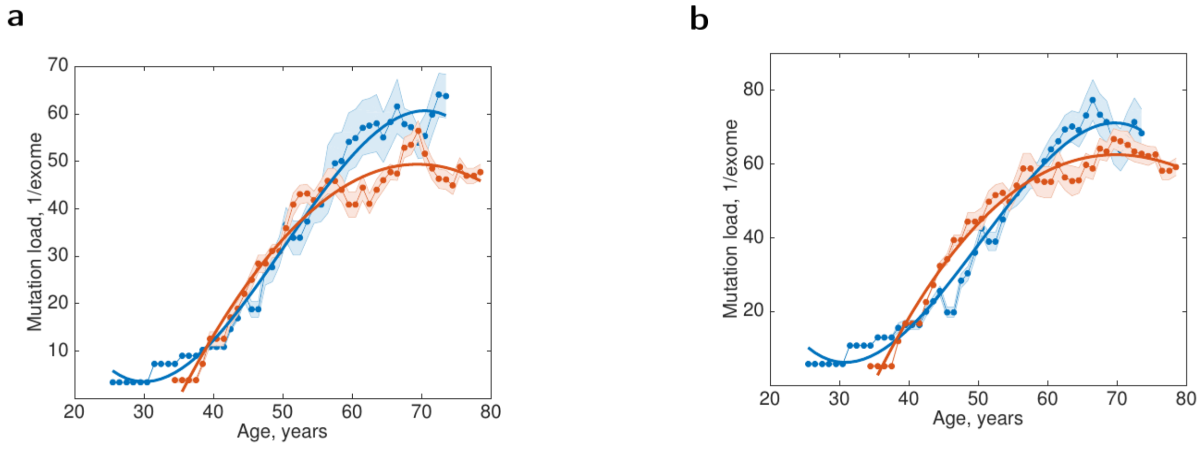

Supplementary Figure 14 | Characteristic somatic mutation load in pancreatic adenocarcinoma (PAAD), all mutations. Men (blue) vs. women (red). **a.** BCGSC, IlluminaHiSeq DNASeq, automated pipeline. **b.** BI, IlluminaGA DNASeq, automated pipeline.

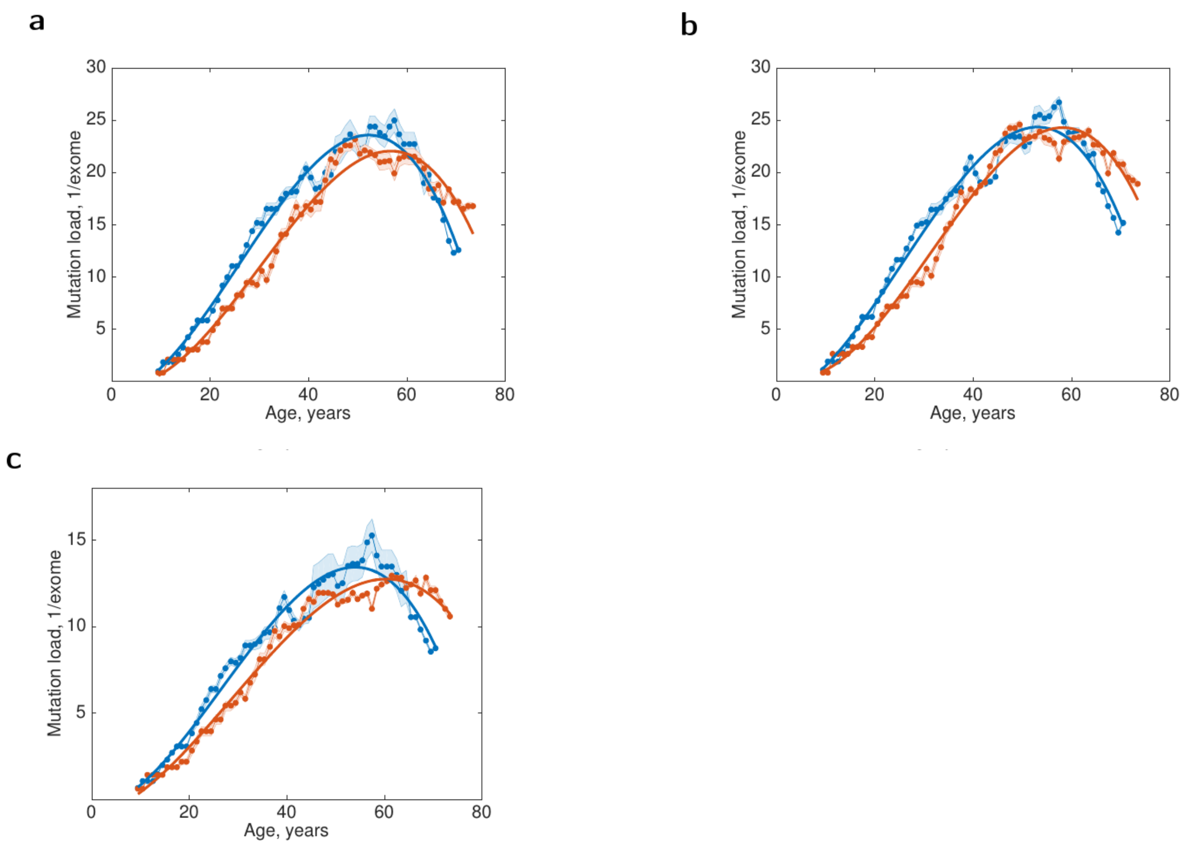

Supplementary Figure 15 | Characteristic somatic mutation load in pheochromocytoma and paraganglioma (PCPG), all mutations. Men (blue) vs. women (red). **a.** BCM, IlluminaGA DNASeq, automated pipeline. **b.** BI, IlluminaGA DNASeq, automated pipeline. **c.** UCSC, IlluminaGA DNASeq, automated pipeline.

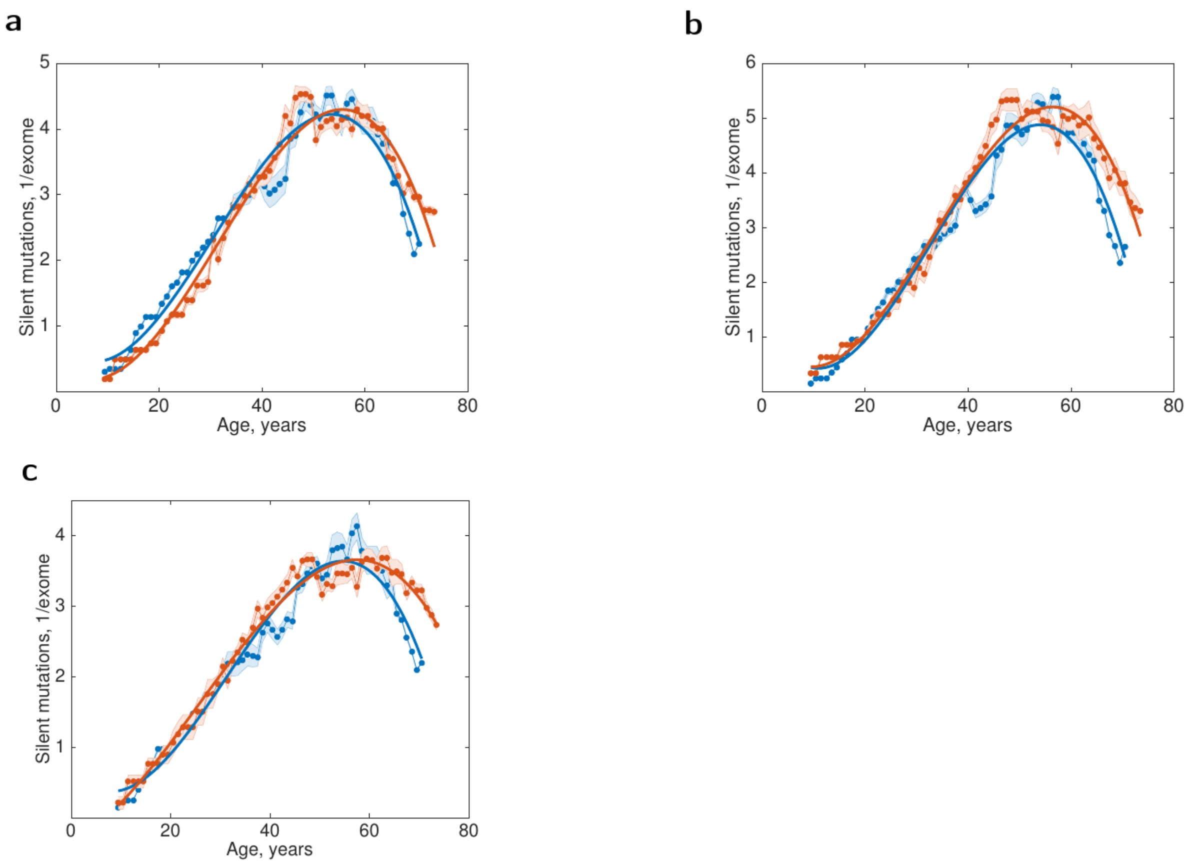

Supplementary Figure 16 | Characteristic somatic mutation load in pheochromocytoma and paraganglioma (PCPG), silent mutations only. Men (blue) vs. women (red). **a.** BCM, IlluminaGA DNASeq, automated pipeline. **b.** BI, IlluminaGA DNASeq, automated pipeline. **c.** UCSC, IlluminaGA DNASeq, automated pipeline.

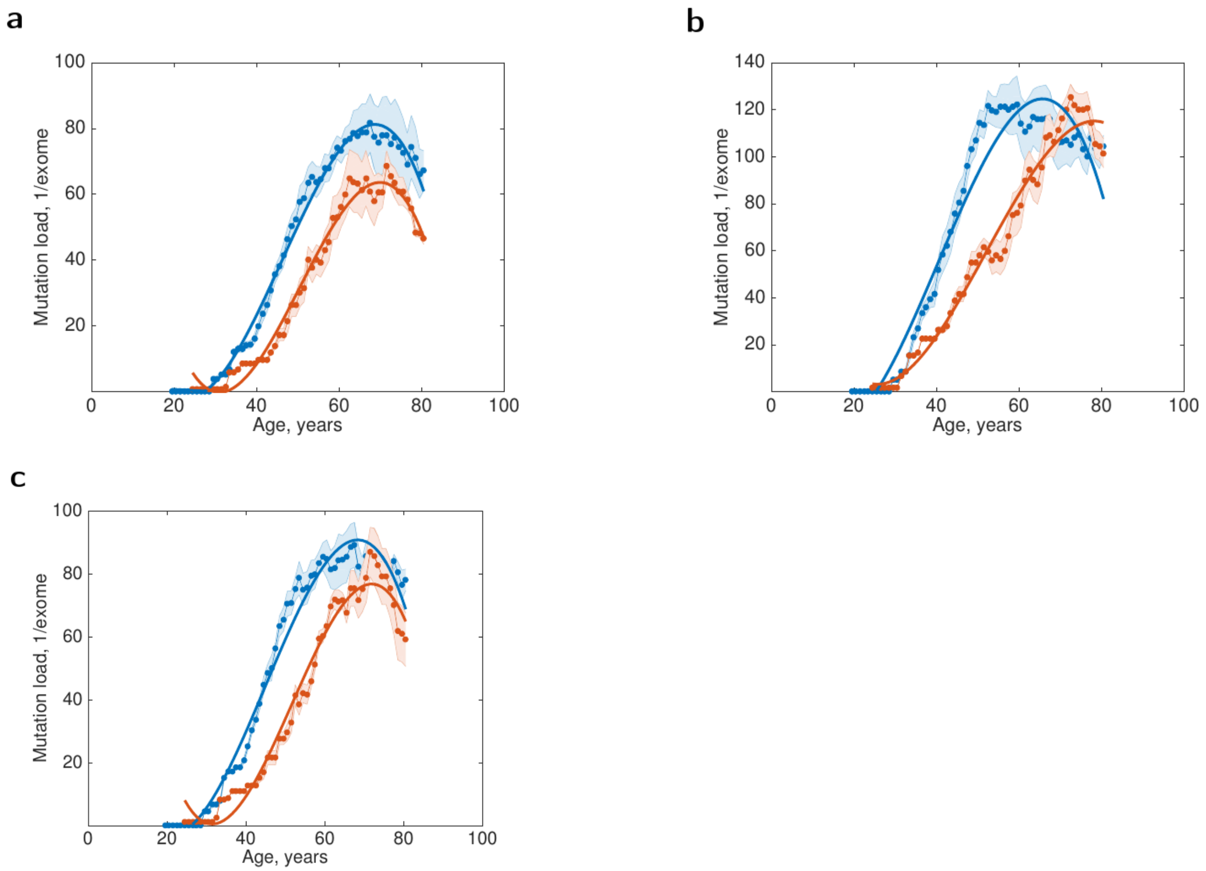

Supplementary Figure 17 | Characteristic somatic mutation load in stomach adenocarcinoma (STAD), all mutations. Men (blue) vs. women (red). **a.** BCM, IlluminaGA DNASeq, automated pipeline. **b.** BI, IlluminaGA DNASeq, automated pipeline. **c.** BI, IlluminaGA DNASeq, curated pipeline.

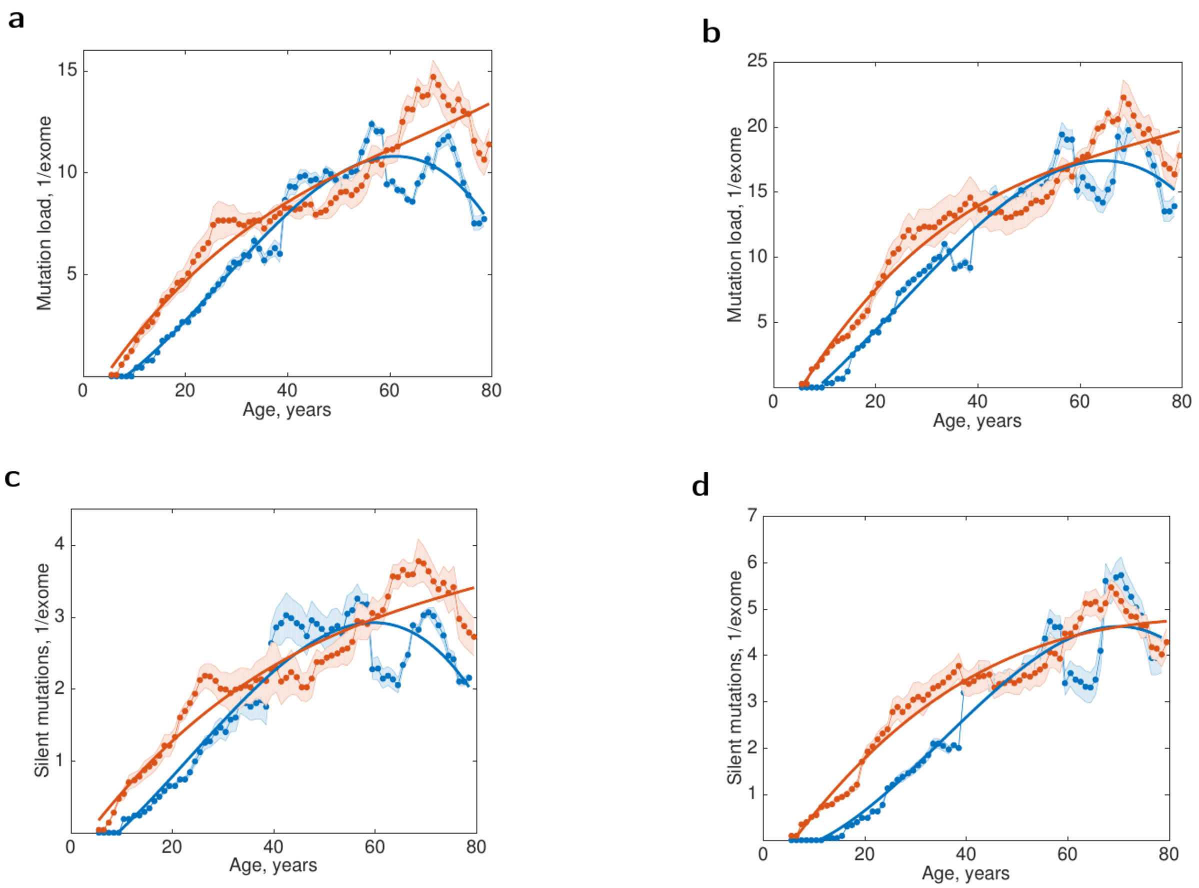

Supplementary Figure 18 | Characteristic somatic mutation load in thyroid carcinoma (THCA). Men (blue) vs. women (red). **a.** BCM, IlluminaGA DNASeq, automated pipeline, all mutations. **b.** BI, IlluminaGA DNASeq, all mutations. **c.** BCM, IlluminaGA DNASeq, automated pipeline, silent mutations only. **d.** BI, IlluminaGA DNASeq, silent mutations only.

Characteristic somatic mutation load vs. cancer incidence

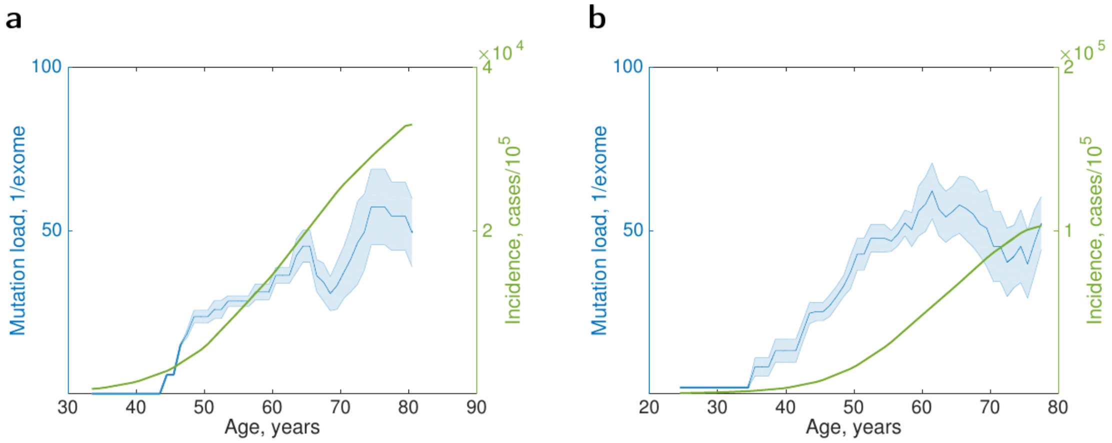

Supplementary Figure 19 | Characteristic somatic mutation load vs. cancer incidence in bladder urothelial carcinoma (BLCA). **a.** Women, BI, IlluminaGA DNASeq, curated pipeline. **b.** Men, BI, IlluminaGA DNASeq, curated pipeline.

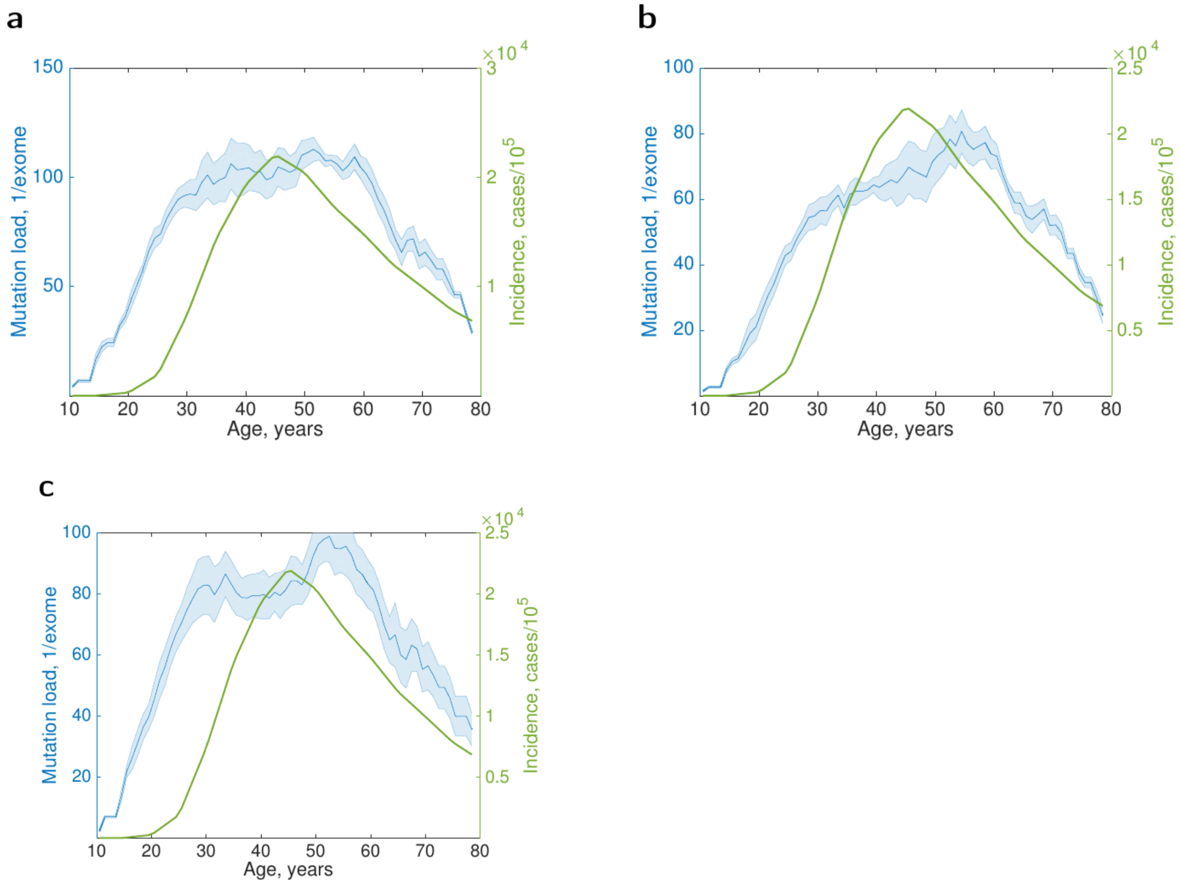

Supplementary Figure 20 | Characteristic somatic mutation load vs. cancer incidence in cervical squamous cell carcinoma and endocervical adenocarcinoma (CESC). **a.** BCGSC, IlluminaHiSeq, automated pipeline. **b.** UCSC, IlluminaGA, automated pipeline. **c.** WUSM, IlluminaGA, curated pipeline.

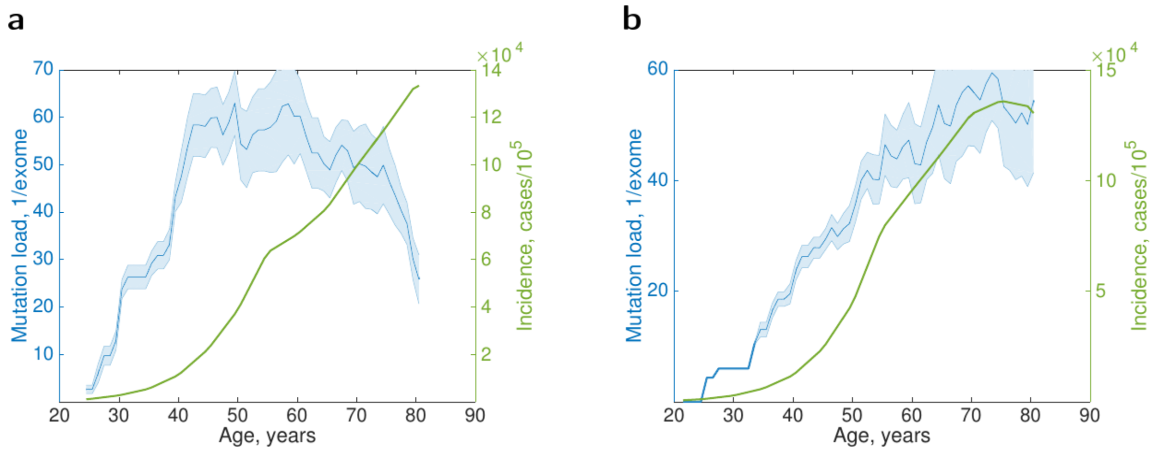

Supplementary Figure 21 | Characteristic somatic mutation load vs. cancer incidence in colon adenocarcinoma (COAD). **a.** Women, BCM, IlluminaGA. **b.** Men, BCM, IlluminaGA.

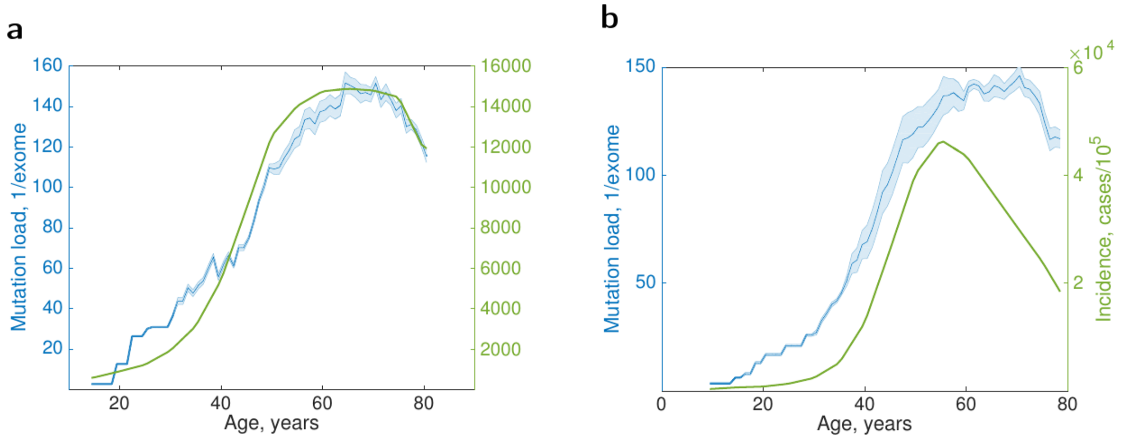

Supplementary Figure 22 | Characteristic somatic mutation load vs. cancer incidence in head and neck squamous cell carcinoma (HNSC). **a.** Women, BI, IlluminaGA, automated pipeline. **b.** Men, BI, IlluminaGA, automated pipeline.

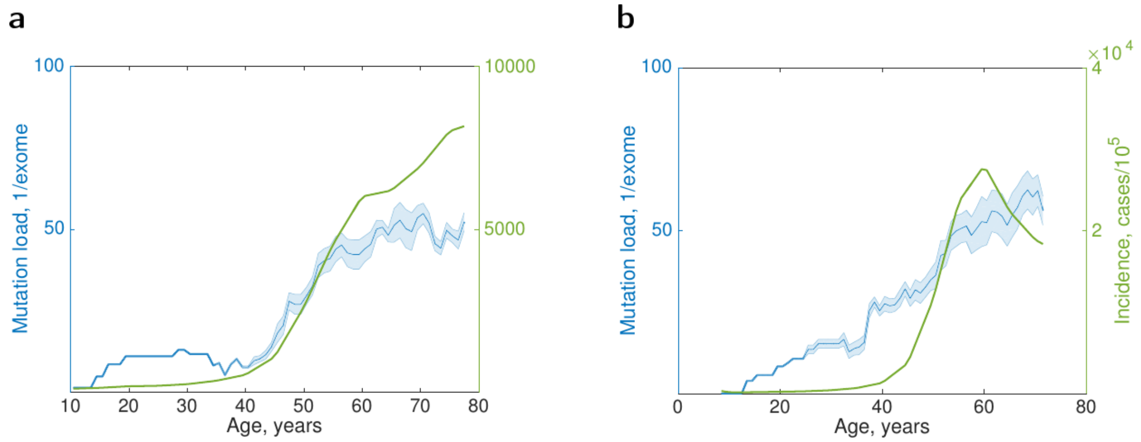

Supplementary Figure 23 | Characteristic somatic mutation load vs. cancer incidence in liver hepatocellular carcinoma (LIHC). **a.** Women, BCM, IlluminaGA, automated pipeline. **b.** Men, BCM, IlluminaGA, automated pipeline.

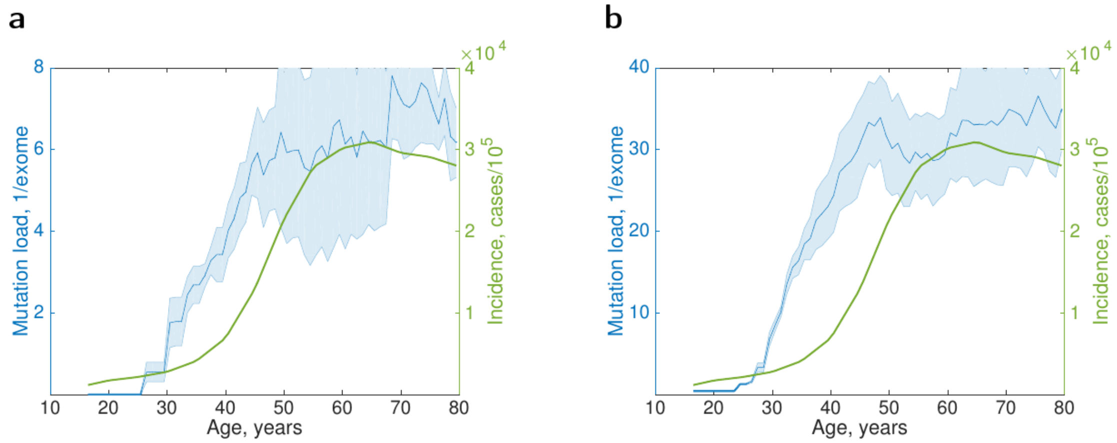

Supplementary Figure 24 | Characteristic somatic mutation load vs. cancer incidence in ovarian serous cystadenocarcinoma (OV). **a.** BCM, SOLiD, curated pipeline. **b.** WUSM, IlluminaGA.

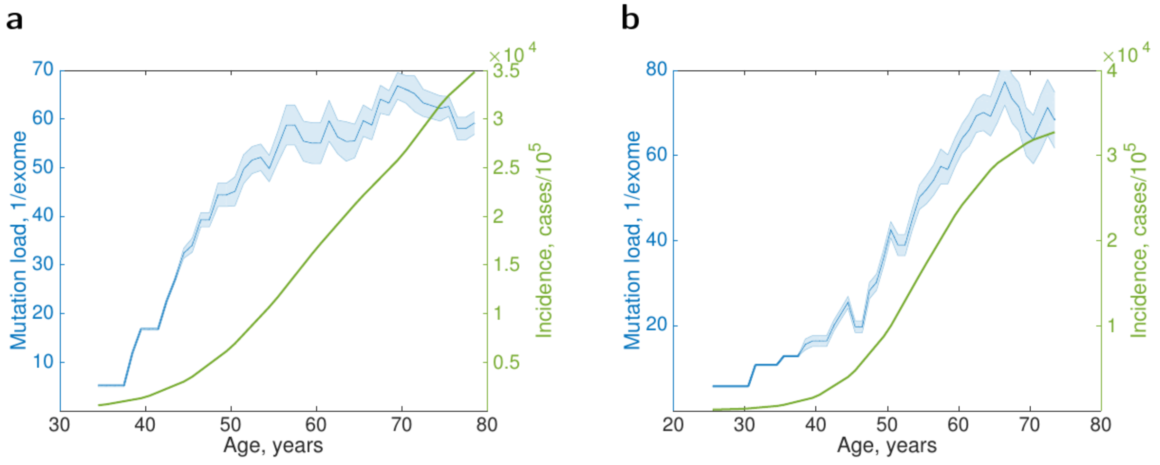

Supplementary Figure 25 | Characteristic somatic mutation load vs. cancer incidence in pancreatic adenocarcinoma (PAAD). **a.** Women, BCM, IlluminaGA, automated pipeline. **b.** Men, BCM, IlluminaGA, automated pipeline.

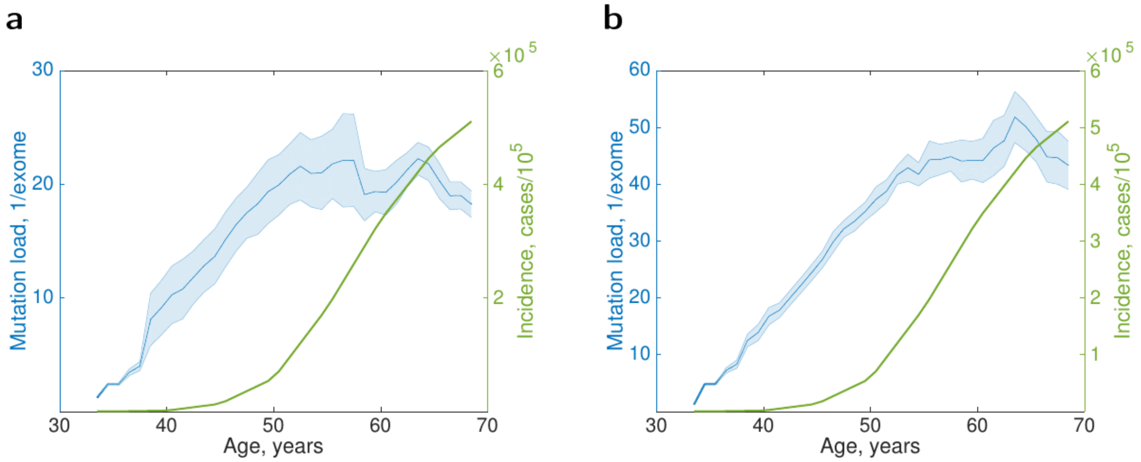

Supplementary Figure 26 | Characteristic somatic mutation load vs. cancer incidence in prostate adenocarcinoma (PRAD). **a.** BCM, IlluminaGA, automated pipeline. **b.** BI, IlluminaGA, automated pipeline.

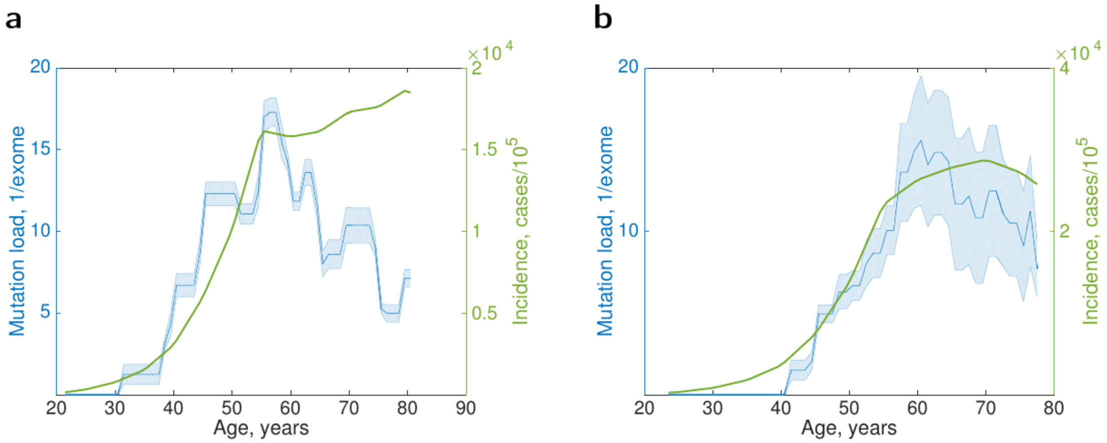

Supplementary Figure 27 | Characteristic somatic mutation load vs. cancer incidence in rectum adenocarcinoma (READ). **a.** Women, BCM, SOLiD. **b.** Men, BCM, SOLiD.

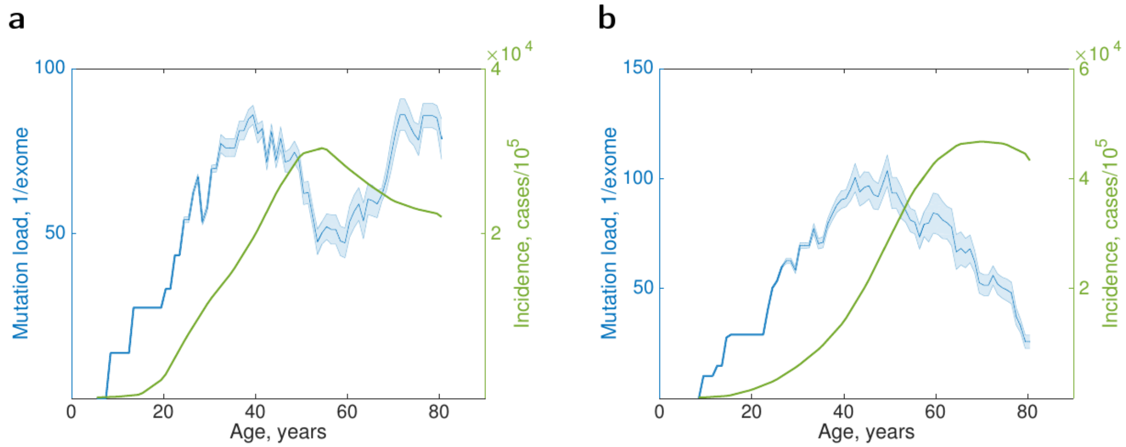

Supplementary Figure 28 | Characteristic somatic mutation load vs. cancer incidence in skin cutaneous melanoma (SKCM). **a.** Women, BCM, IlluminaGA, automated pipeline. **b.** Men, BCM, IlluminaGA, automated pipeline.

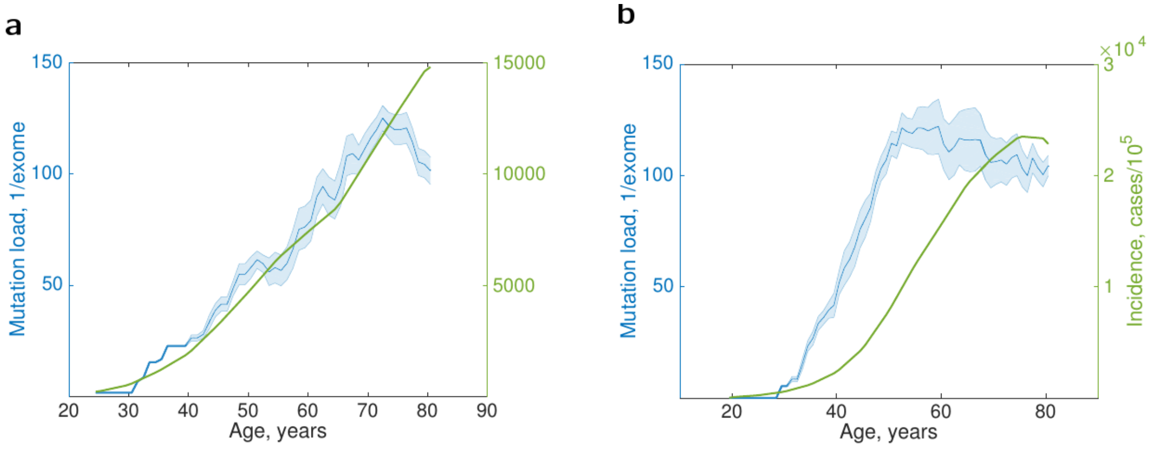

Supplementary Figure 29 | Characteristic somatic mutation load vs. cancer incidence in stomach adenocarcinoma (STAD). **a.** Women, BI, IlluminaGA, automated pipeline. **b.** Men, BI, IlluminaGA, automated pipeline.

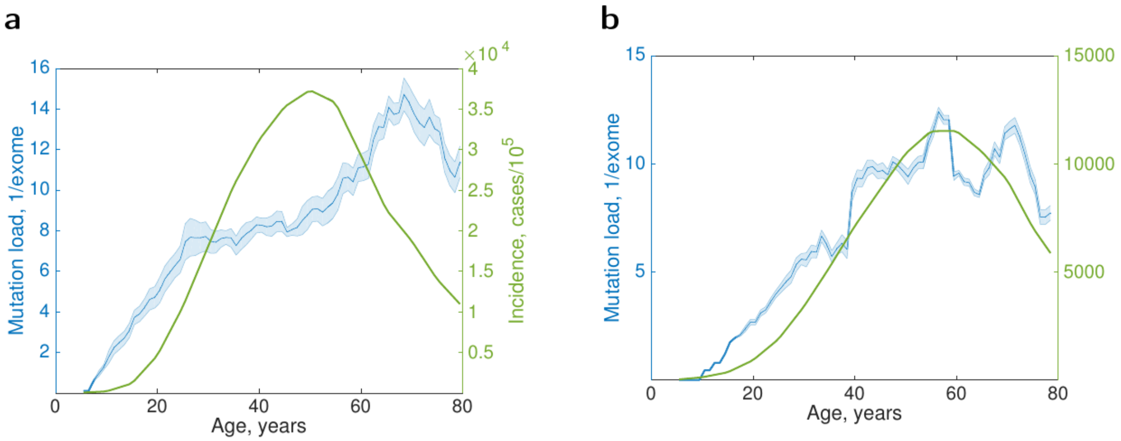

Supplementary Figure 30 | Characteristic somatic mutation load vs. cancer incidence in thyroid carcinoma (THCA). **a.** Women, BCM, IlluminaGA, automated pipeline. **b.** Men, BCM, IlluminaGA, automated pipeline.

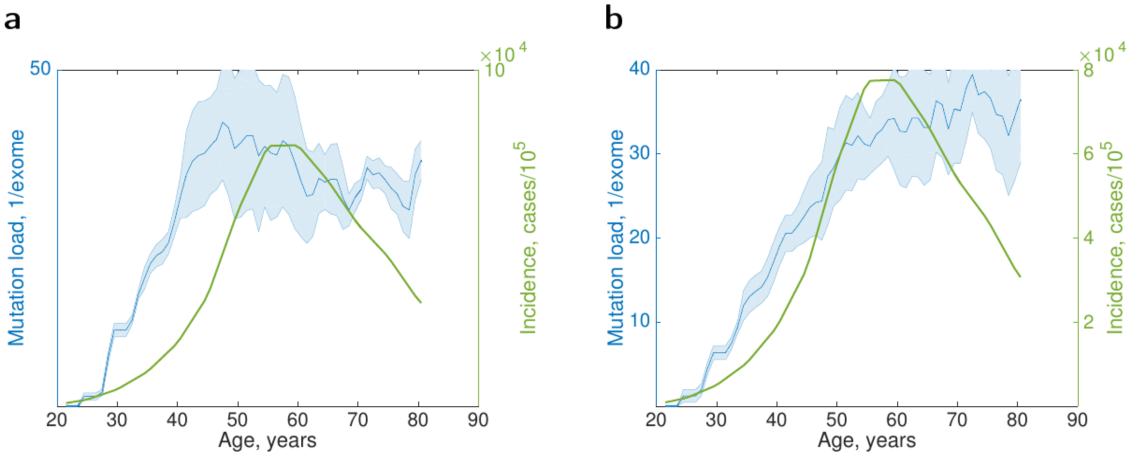

Supplementary Figure 31 | Characteristic somatic mutation load vs. cancer incidence in uterine corpus endometrial carcinoma (UCEC). **a.** BI, IlluminaGA. **b.** WUSM, IlluminaGA.

Growth of cancer incidence with age in different countries

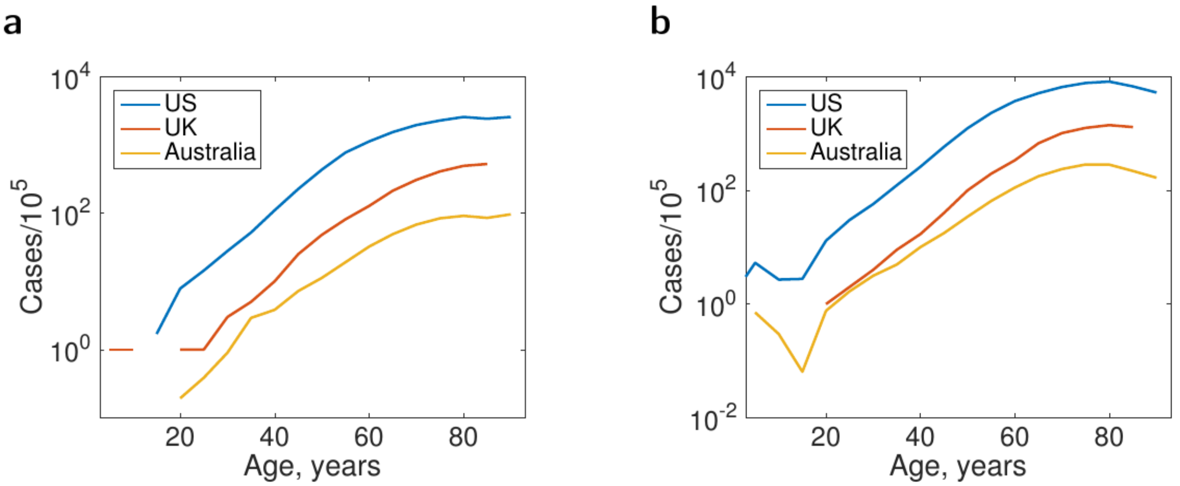

Supplementary Figure 32 | Incidence of bladder cancer in US, UK and Australia, semilog scale. **a.** In women. **b.** In men.

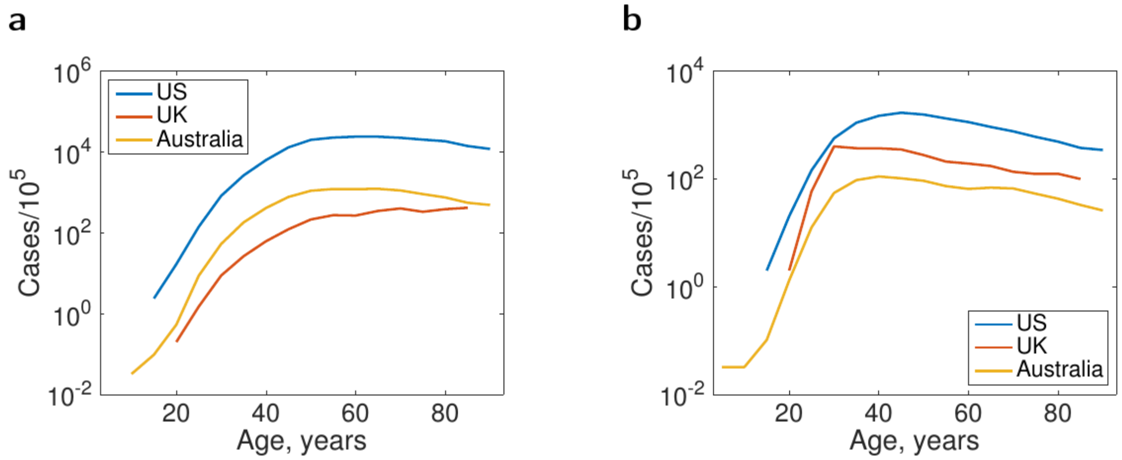

Supplementary Figure 33 | Incidence of breast and cervical cancer in US, UK and Australia, semilog scale. **a.** Breast cancer. **b.** Cervical cancer.

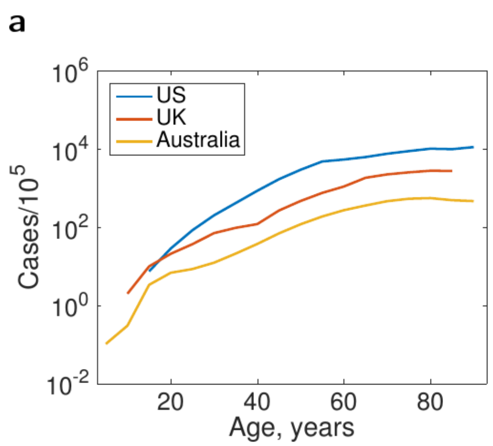

Supplementary Figure 34 | Incidence of colon cancer in US, UK and Australia, semilog scale. **a.** In women. **b.** In men.

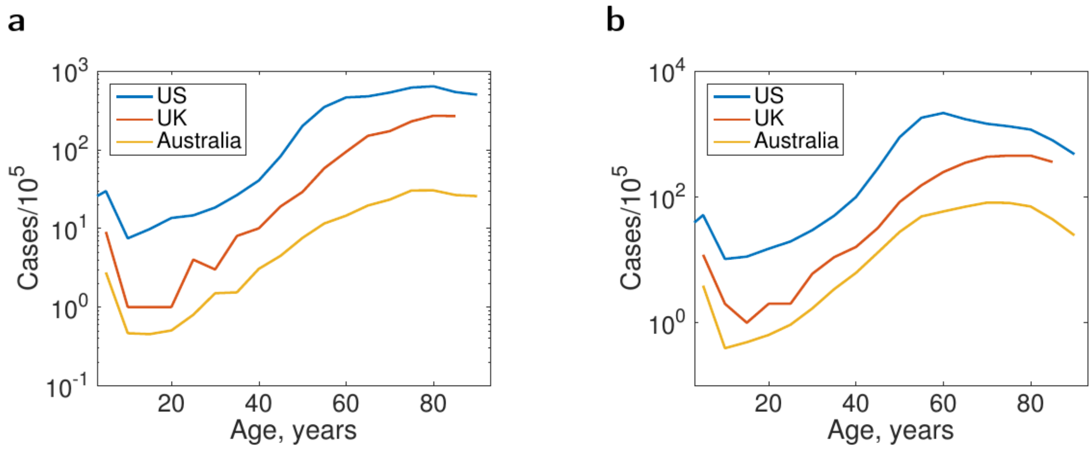

Supplementary Figure 35 | Incidence of liver cancer in US, UK and Australia, semilog scale. **a.** In women. **b.** In men.

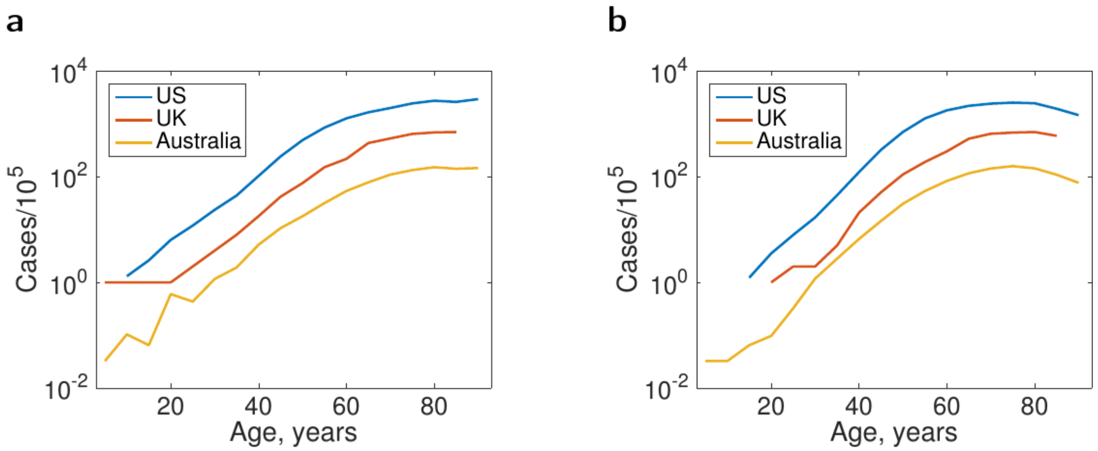

Supplementary Figure 36 | Incidence of pancreatic cancer in US, UK and Australia, semilog scale. **a.** In women. **b.** In men.

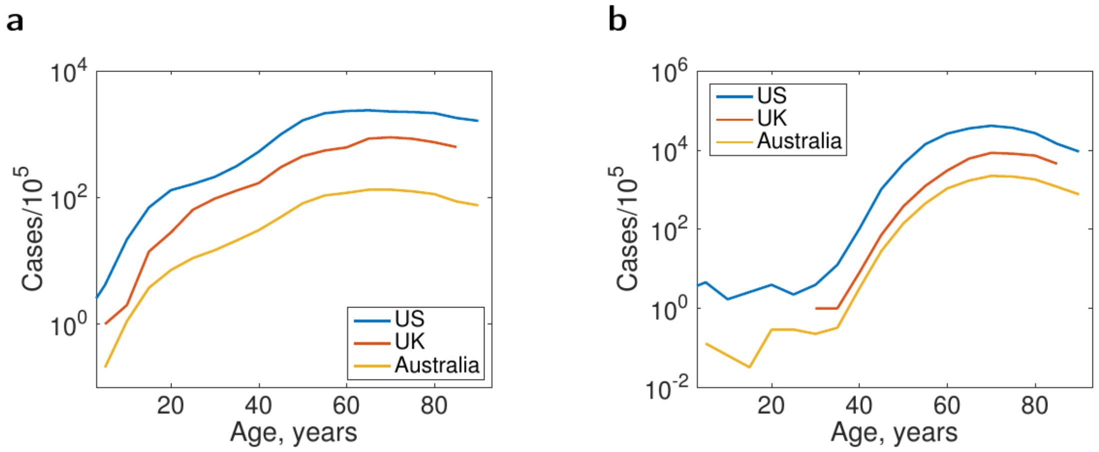

Supplementary Figure 37 | Incidence of ovarian and prostate cancer in US, UK and Australia, semilog scale. **a.** Ovarian cancer. **b.** Prostate cancer.

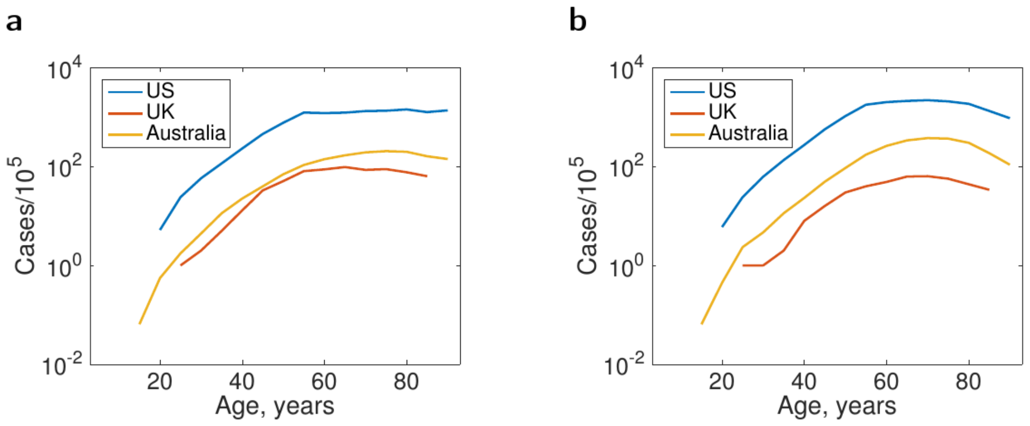

Supplementary Figure 38 | Incidence of rectal cancer in US, UK and Australia, semilog scale. **a.** In women. **b.** In men.

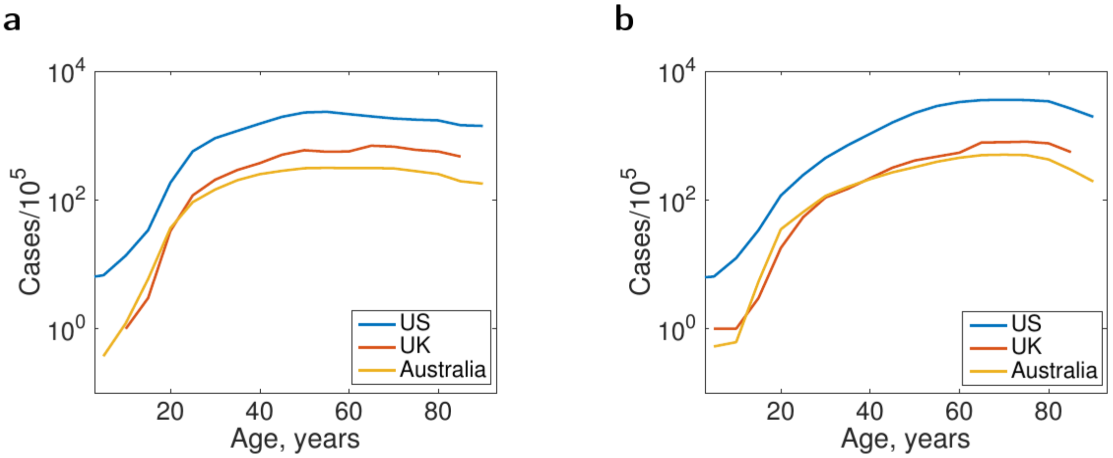

Supplementary Figure 39 | Incidence of skin melanoma in US, UK and Australia, semilog scale. **a.** In women. **b.** In men.

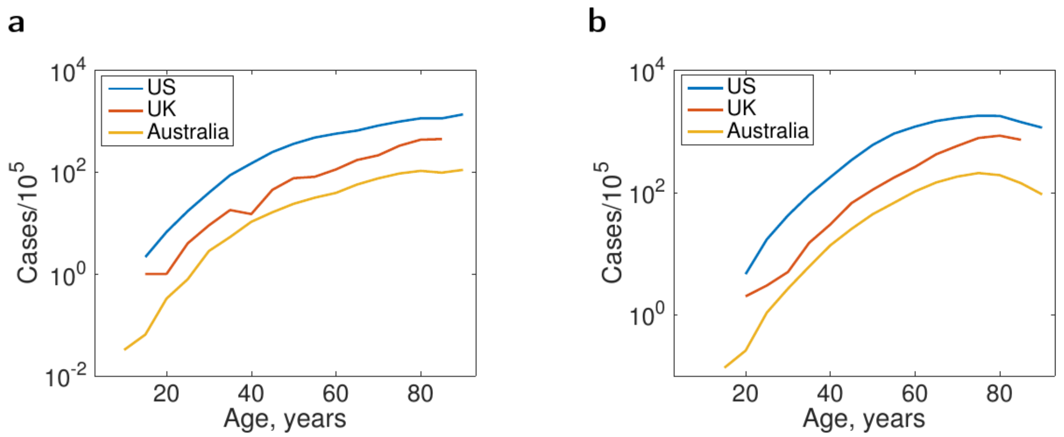

Supplementary Figure 40 | Incidence of stomach melanoma in US, UK and Australia, semilog scale. **a.** In women. **b.** In men.

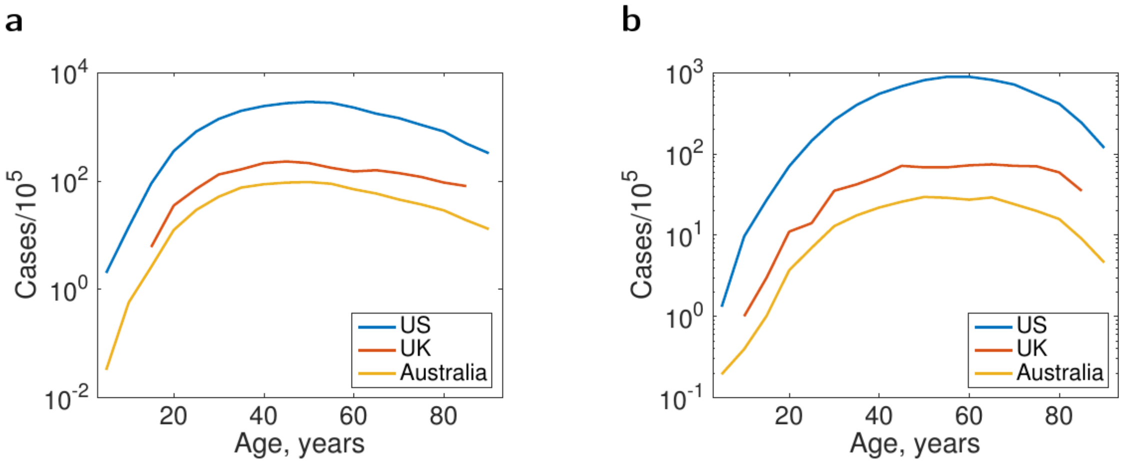

Supplementary Figure 41 | Incidence of thyroid cancer in US, UK and Australia, semilog scale. **a.** In women. **b.** In men.

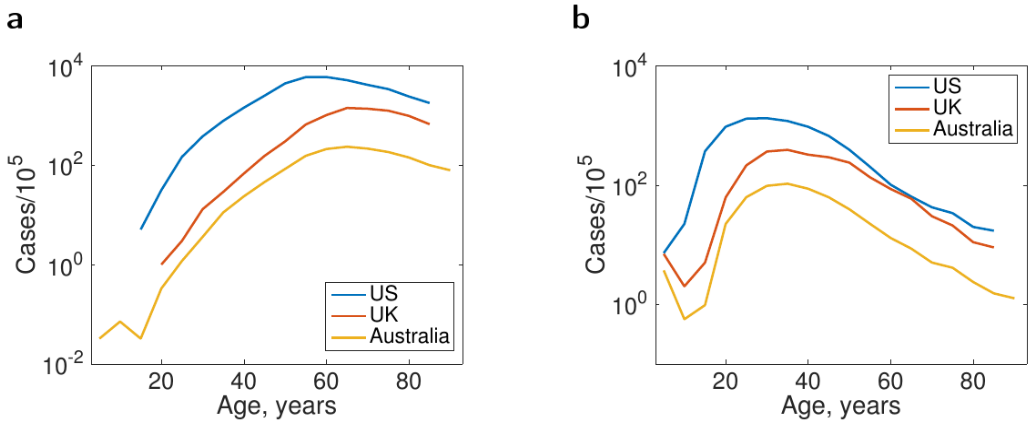

Supplementary Figure 42 | Incidence of uterine and testicular cancer in US, UK and Australia, semilog scale. **a.** Uterine cancer. **b.** Testicular cancer.

Mutation counting in whole genomes

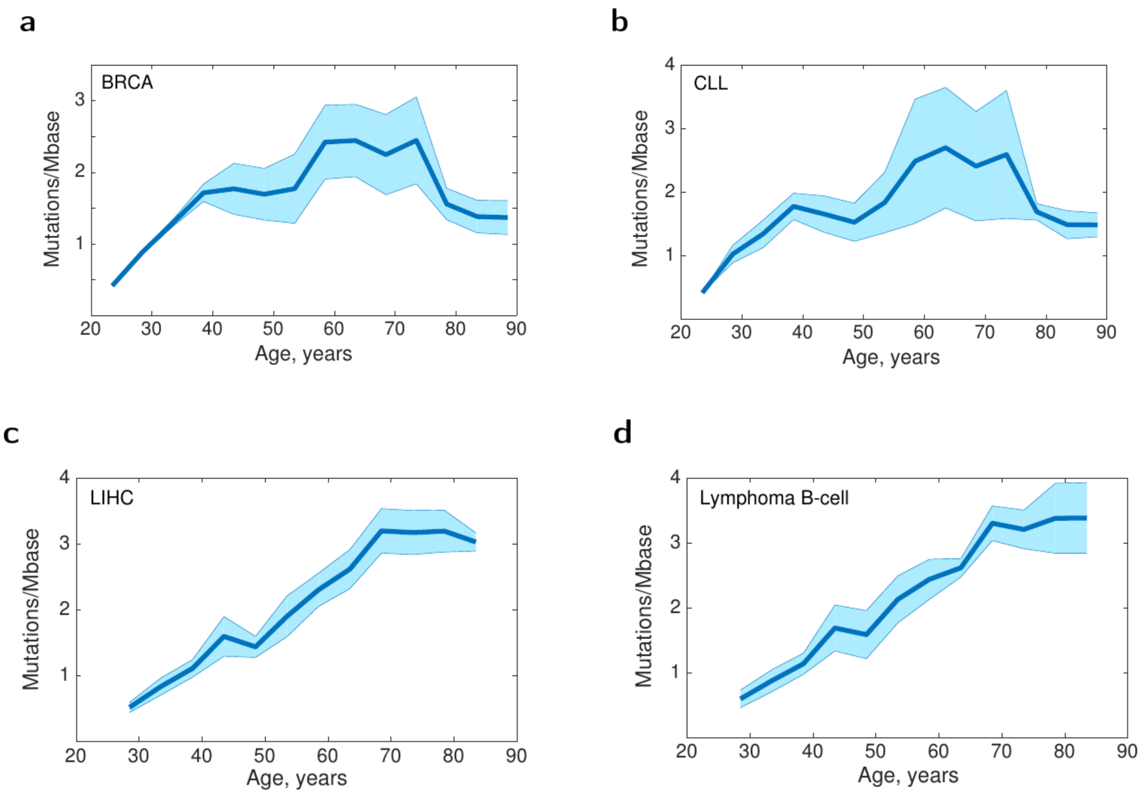

Supplementary Figure 43 | Behavior of mutational load with age in whole genomes. **a.** Breast invasive carcinoma. **b.** Chronic lymphocytic leukemia. **c.** Liver hepatic carcinoma. **d.** B-cell lymphoma.

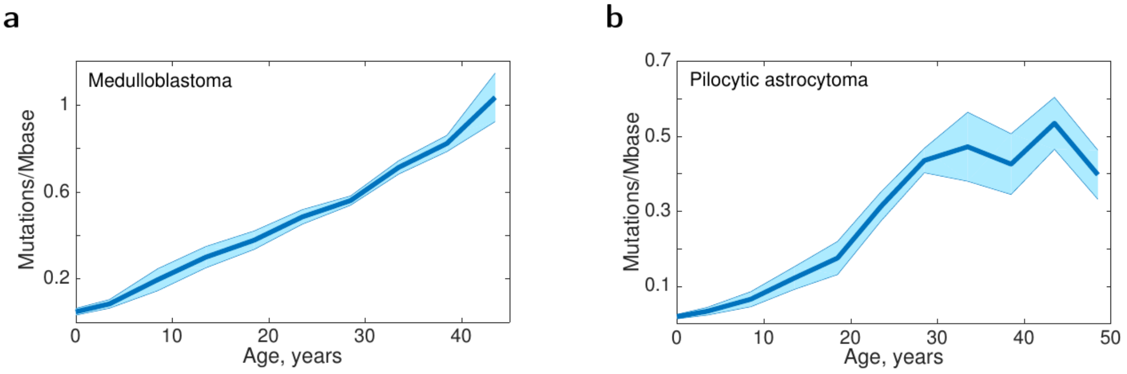

Supplementary Figure 44 | Behavior of mutational load with age in whole genomes. **a.** Medulloblastoma. **b.** Pilocytic astrocytoma.

Examples of chronological age distributions for samples covered by TCGA

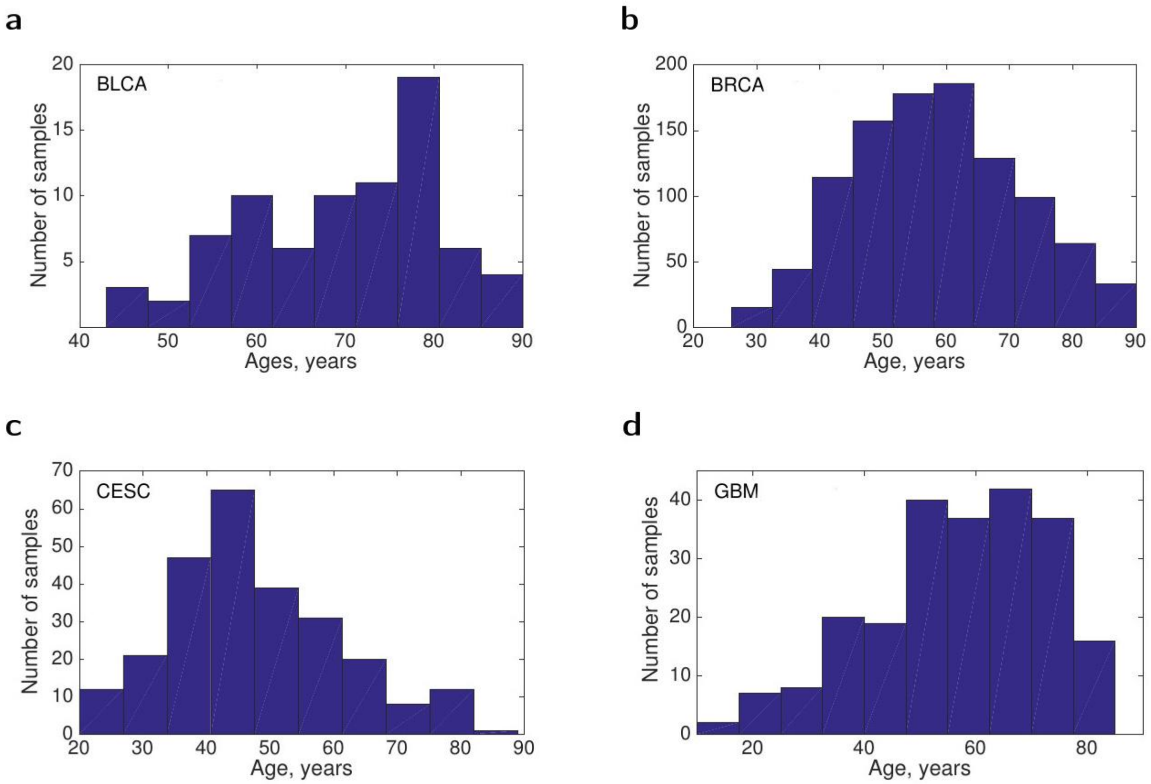

Supplementary Figure 45 | Chronological age distributions for samples covered by TCGA atlas, women. **a.** Bladder adenocarcinoma, BI, automated pipeline. **b.** Breast invasive carcinoma, WUSM, curated pipeline. **c.** Cervical squamous cell carcinoma, BSGSC, IlluminaHiSeq, automated pipeline. **d.** Glioblastoma, BI, Illumina pipeline.

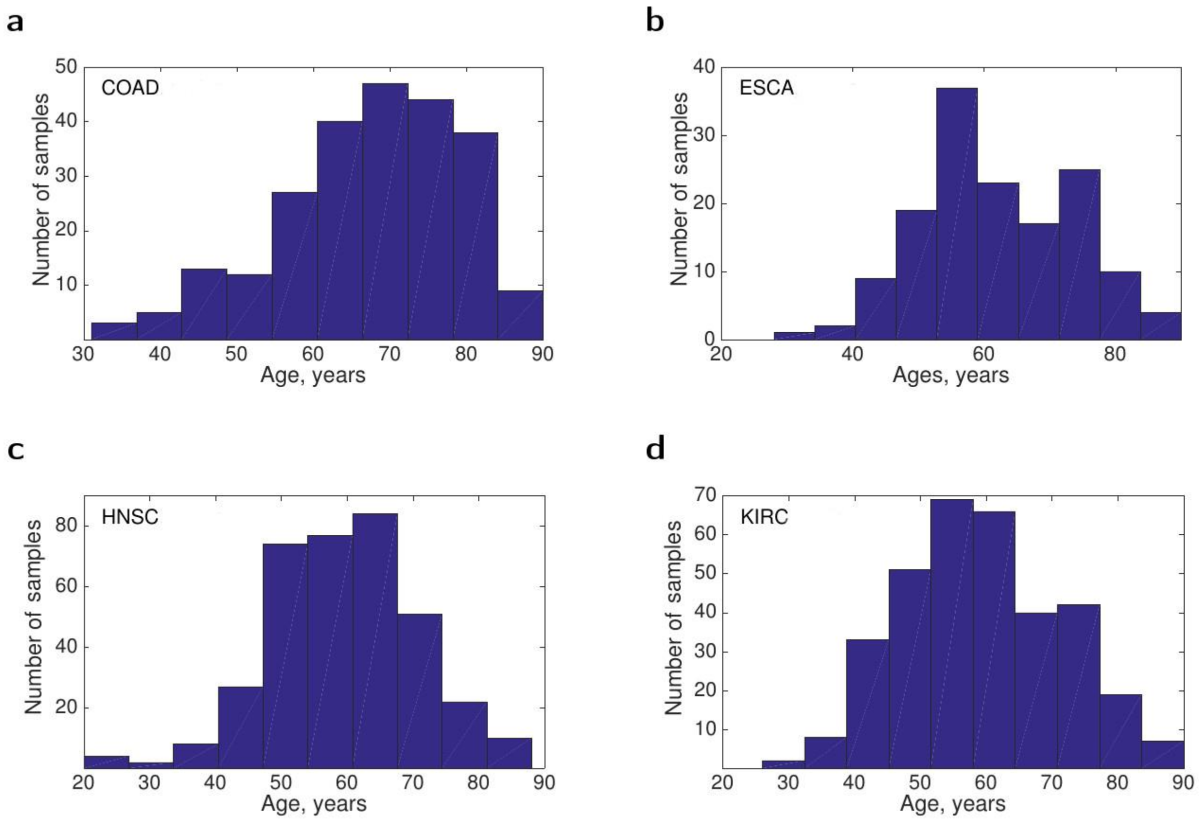

Supplementary Figure 46 | Chronological age distributions for samples covered by TCGA atlas, men. **a.** Colon adenocarcinoma, BCM, IlluminaGA. **b.** Esophageal adenocarcinoma, BCGSC, IlluminaHiSeq, automated pipeline. **c.** Head and neck squamous cell carcinoma. BI, IlluminaGA. **d.** Kidney renal clear cell carcinoma, BI, Illumina, automated pipeline.

Relative weights of different indels and SNVs in the age-correlated somatic mutation load pattern

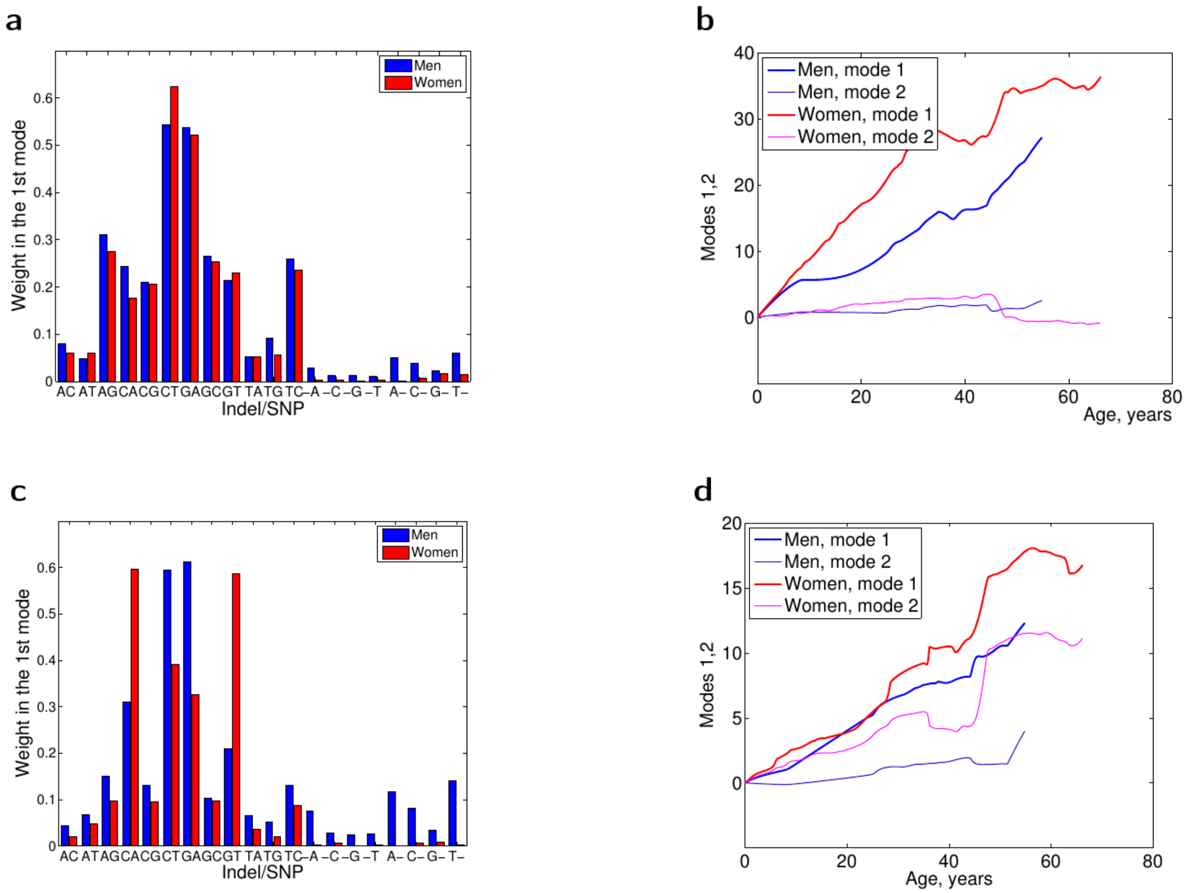

Supplementary Figure 47 | Modal structure of somatic mutation load in adrenocortical carcinoma (ACC). **a.** BCM, IlluminaGA DNASeq, automated pipeline; relative weights of different SNVs and indels. **b.** BCM, IlluminaGA DNASeq, automated pipeline; count estimation using projection on the leading pattern. **c.** BCM, IlluminaGA DNASeq, curated pipeline relative weights of different SNVs and indels. **d.** BCM, IlluminaGA DNASeq, automated pipeline; count estimation using projection on the leading pattern.

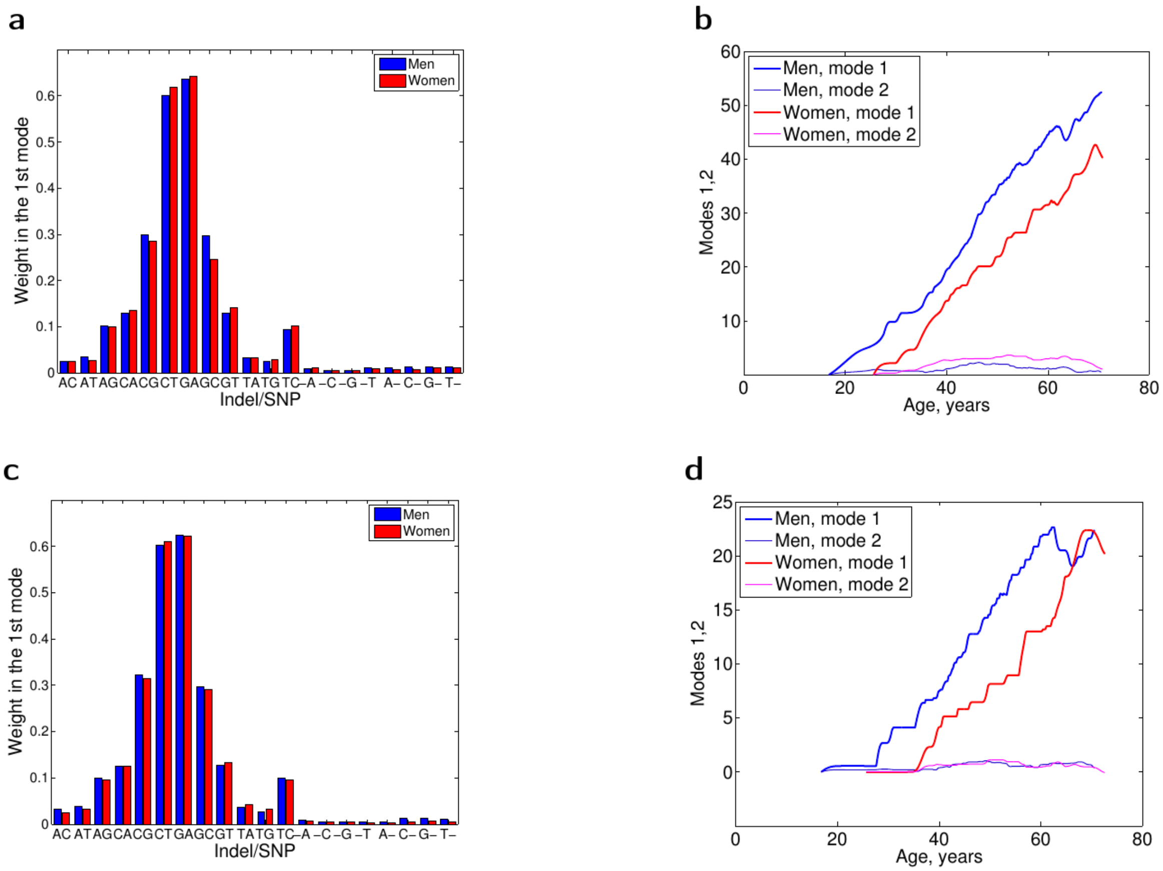

Supplementary Figure 48 | Modal structure of somatic mutation load in bladder urothelial carcinoma (BLCA). **a.** BI, IlluminaGA DNASeq, automated pipeline; relative weights of different SNVs and indels. **b.** BI, IlluminaGA DNASeq, automated pipeline; count estimation using projection on the leading pattern. **c.** BI, IlluminaGA DNASeq, curated pipeline; relative weights of different SNVs and indels. **d.** BI, IlluminaGA DNASeq, curated pipeline; count estimation using projection on the leading pattern.

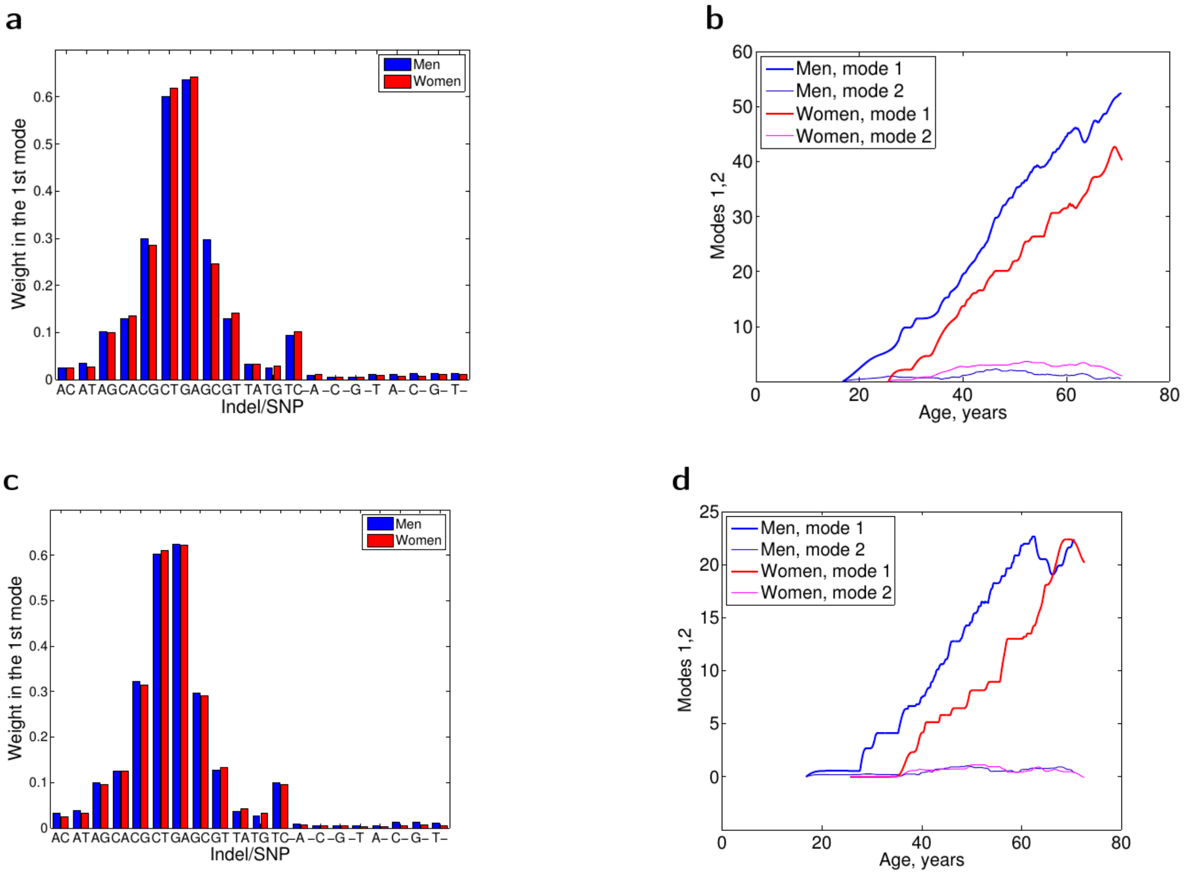

Supplementary Figure 49 | Modal structure of somatic mutation load in breast invasive carcinoma (BRCA). **a.** WUSM, IlluminaGA DNASeq, curated pipeline; relative weights of different SNVs and indels. **b.** WUSM, IlluminaGA DNASeq, curated pipeline; count estimation using projection on the leading pattern. **c.** WUSM, IlluminaGA DNASeq; relative weights of different SNVs and indels. **d.** WUSM, IlluminaGA DNASeq; count estimation using projection on the leading pattern.

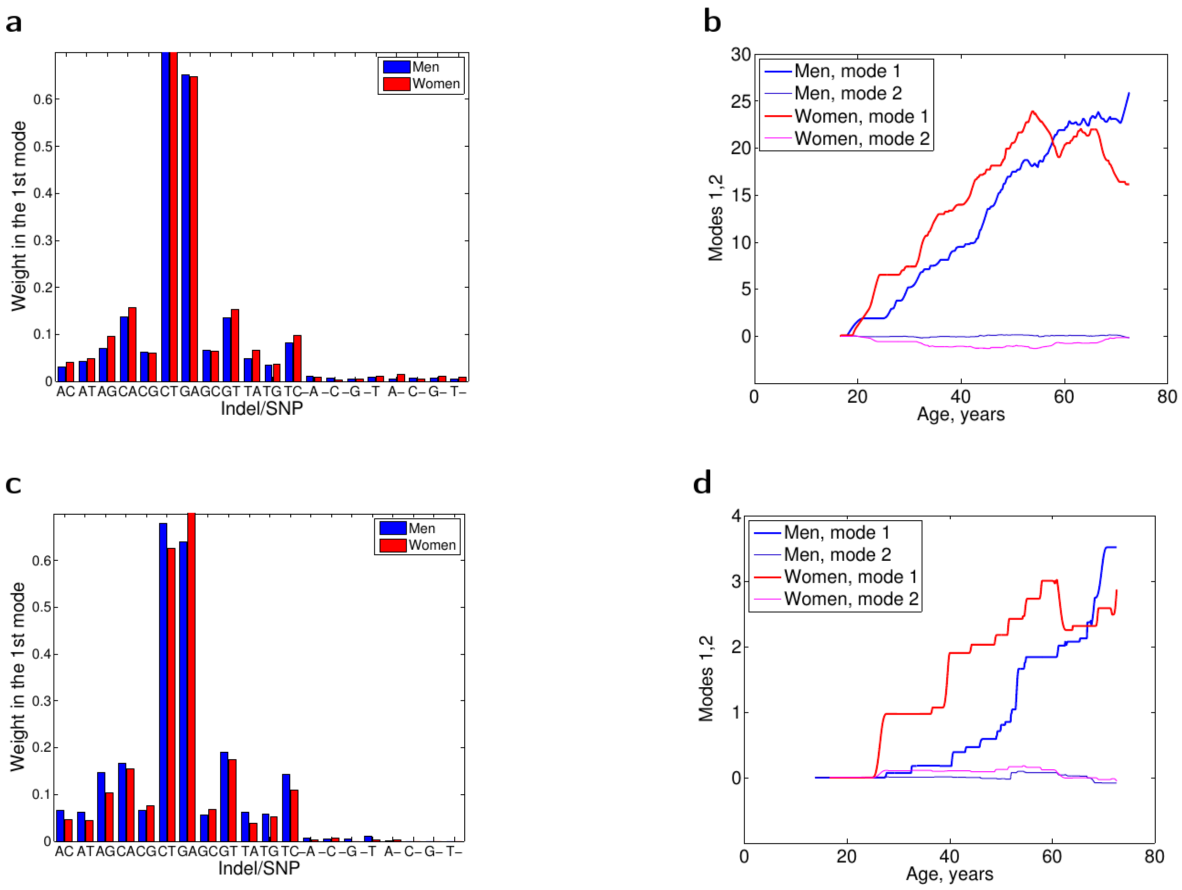

Supplementary Figure 50 | Modal structure of somatic mutation load in colon adenocarcinoma (COAD). **a.** BCM, IlluminaGA DNASeq; relative weights of different SNVs and indels. **b.** BCM, IlluminaGA DNASeq; count estimation using projection on the leading pattern. **c.** BCM, SOLiD DNASeq; relative weights of different SNVs and indels. **d.** BCM, SOLiD DNASeq; count estimation using projection on the leading pattern.

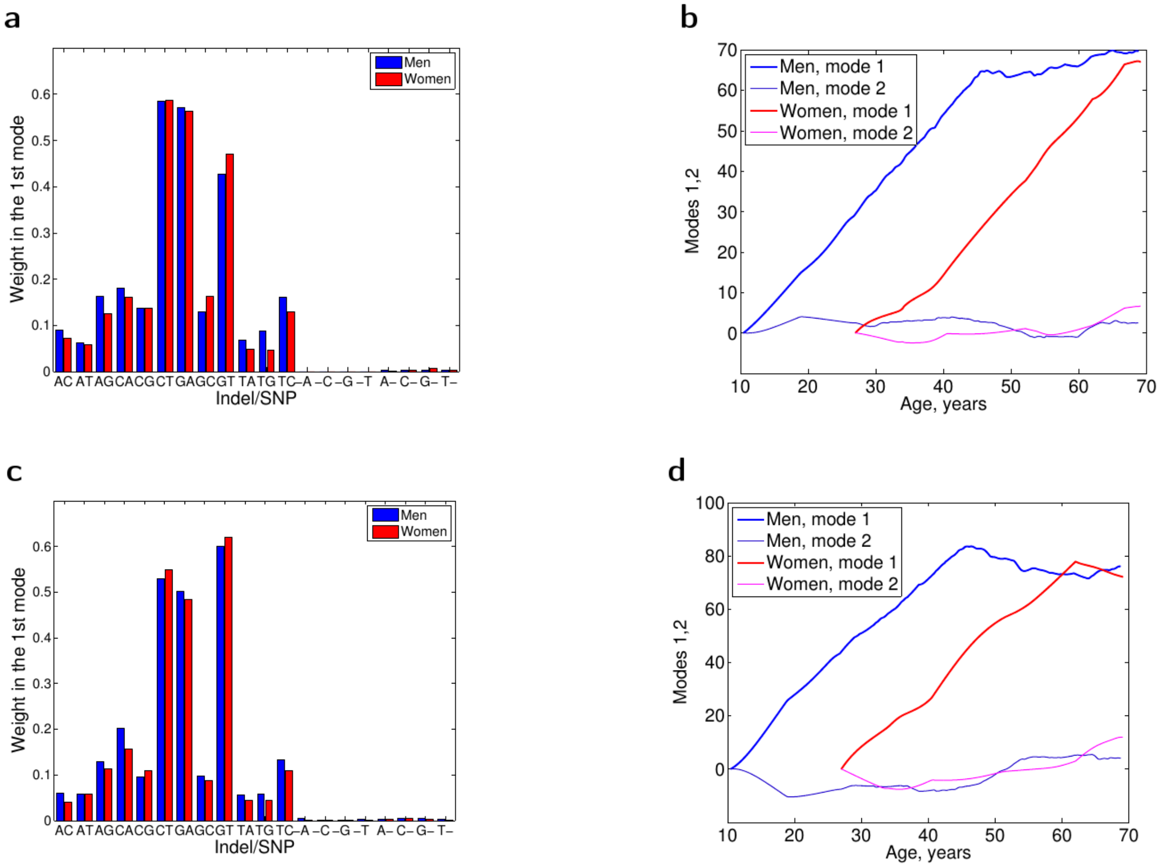

Supplementary Figure 51 | Modal structure of somatic mutation load in esophageal carcinoma (ESCA). **a.** BCGSC, IlluminaHiSeq DNASeq, automated pipeline; relative weights of different SNVs and indels. **b.** BCGSC, IlluminaHiSeq DNASeq, automated pipeline; count estimation using projection on the leading pattern. **c.** BCM, IlluminaGA DNASeq, automated pipeline; relative weights of different SNVs and indels. **d.** BCM, IlluminaGA DNASeq, automated pipeline; count estimation using projection on the leading pattern.

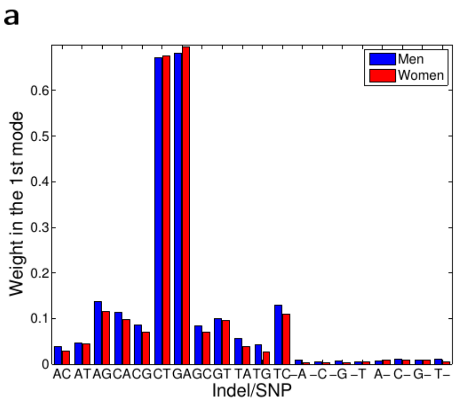

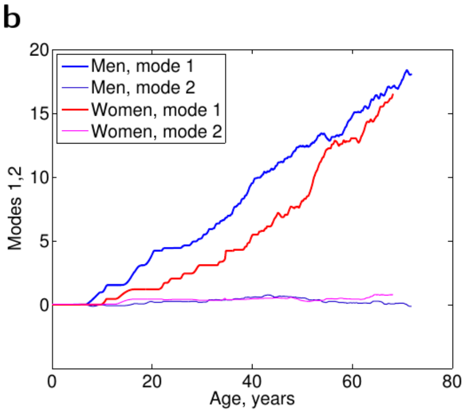

Supplementary Figure 52 | Modal structure of somatic mutation load in glioblastoma multiforme (GBM). **a.** BI, IlluminaGA DNASeq; relative weights of different SNVs and indels. **b.** BI, IlluminaGA DNASeq; count estimation using projection on the leading pattern.

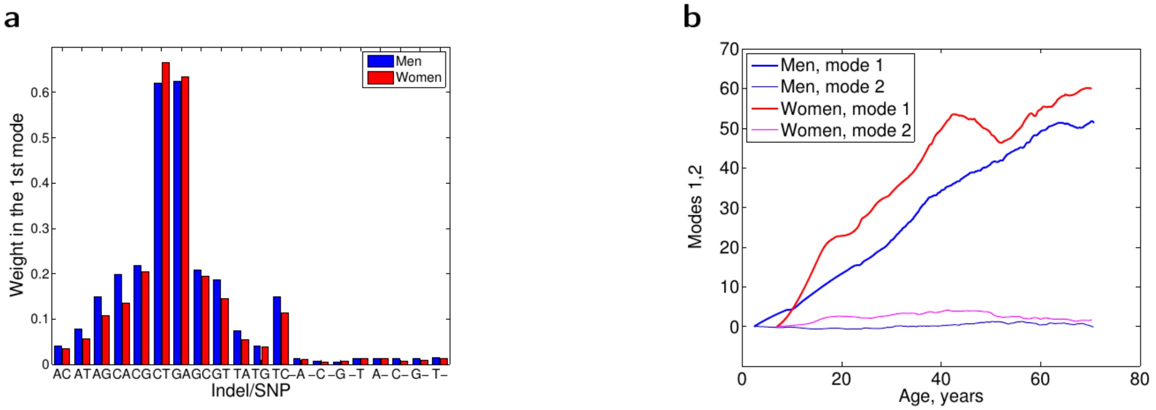

Supplementary Figure 53 | Modal structure of somatic mutation load in head and neck squamous cell carcinoma (HNSC). **a.** BI, IlluminaGA DNASeq, automated pipeline; relative weights of different SNVs and indels. **b.** BI, IlluminaGA DNASeq, automated pipeline; count estimation using projection on the leading pattern.

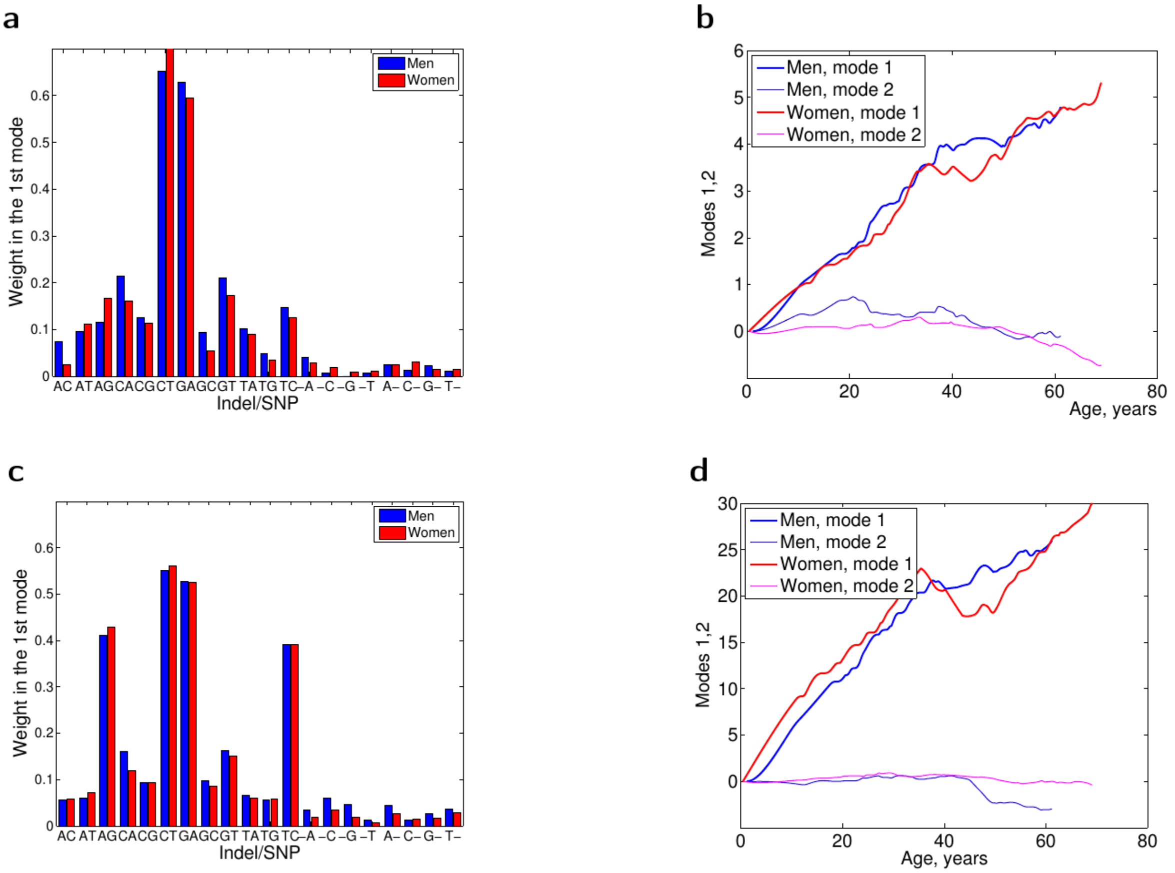

Supplementary Figure 54 | Modal structure of somatic mutation load in kidney chromophobe (KICH). **a.** BCM, IlluminaGA DNASeq, automated pipeline; relative weights of different SNVs and indels. **b.** BCM, IlluminaGA DNASeq, automated pipeline; count estimation using projection on the leading pattern. **c.** BCM, mixed IlluminaGA and SOLiD, curated pipeline; relative weights of different SNVs and indels. **d.** BCM, mixed IlluminaGA and SOLiD, curated pipeline; count estimation using projection on the leading pattern.

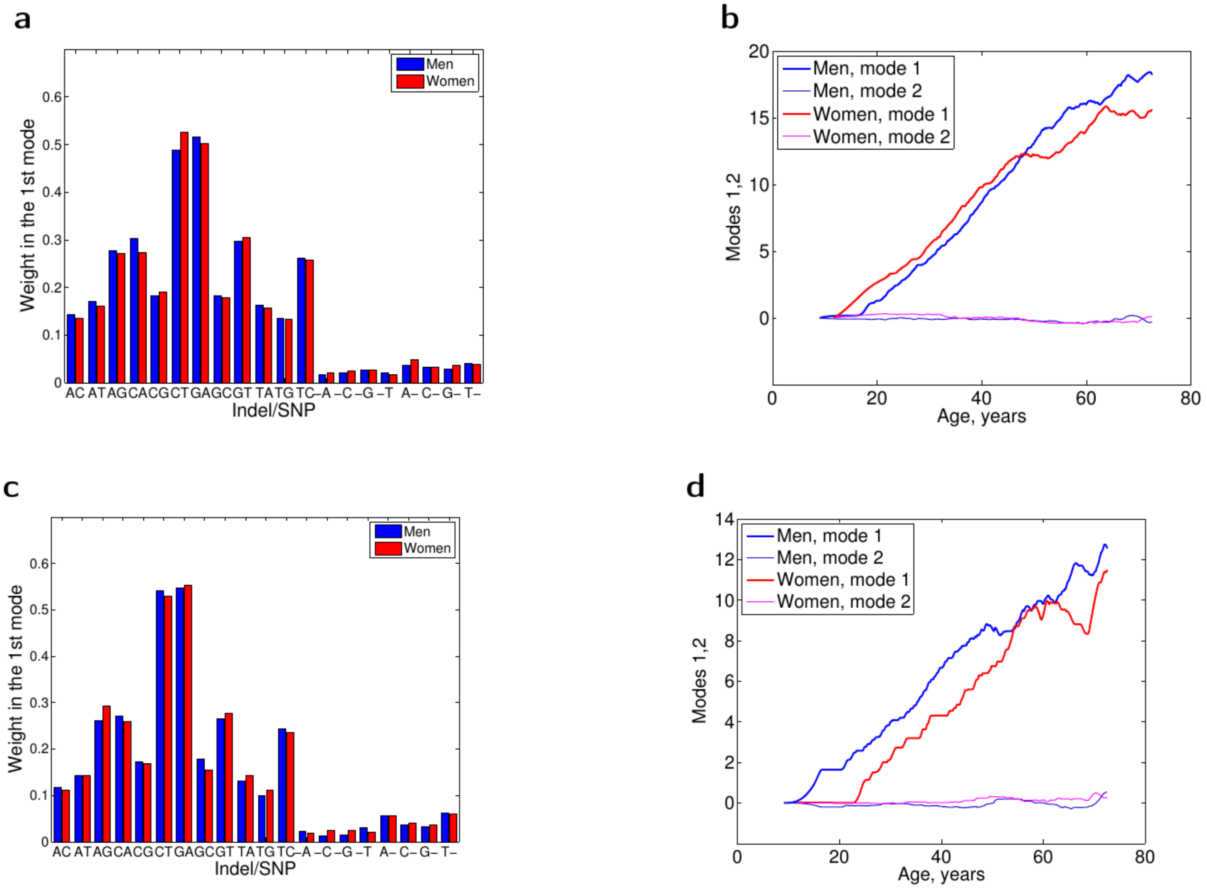

Supplementary Figure 55 | Modal structure of somatic mutation load in kidney renal clear cell carcinoma (KIRC). **a.** BCM, mixed IlluminaGA and SOLiD; relative weights of different SNVs and indels. **b.** BCM, mixed IlluminaGA and SOLiD; count estimation using projection on the leading pattern. **c.** BI, IlluminaGA DNASeq, automated pipeline; relative weights of different SNVs and indels. **d.** BI, IlluminaGA DNASeq, automated pipeline; count estimation using projection on the leading pattern.

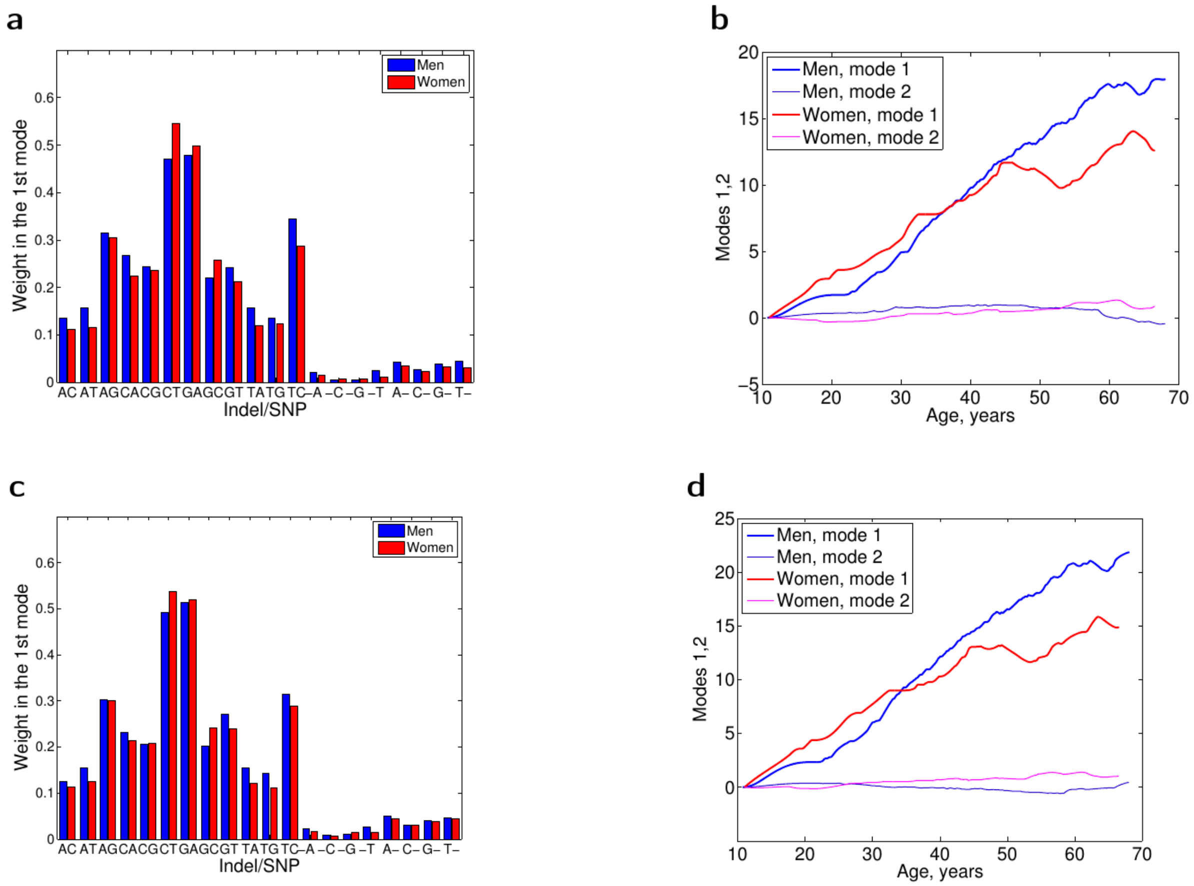

Supplementary Figure 56 | Modal structure of somatic mutation load in kidney renal papillary cell carcinoma (KIRP). **a.** BCM, IlluminaGA DNASeq, curated pipeline; relative weights of different SNVs and indels. **b.** BCM, IlluminaGA DNASeq, curated pipeline; count estimation using projection on the leading pattern. **c.** BCM, mixed IlluminaGA and SOLiD, curated pipeline; relative weights of different SNVs and indels. **d.** BCM, mixed IlluminaGA and SOLiD, curated pipeline; count estimation using projection on the leading pattern.

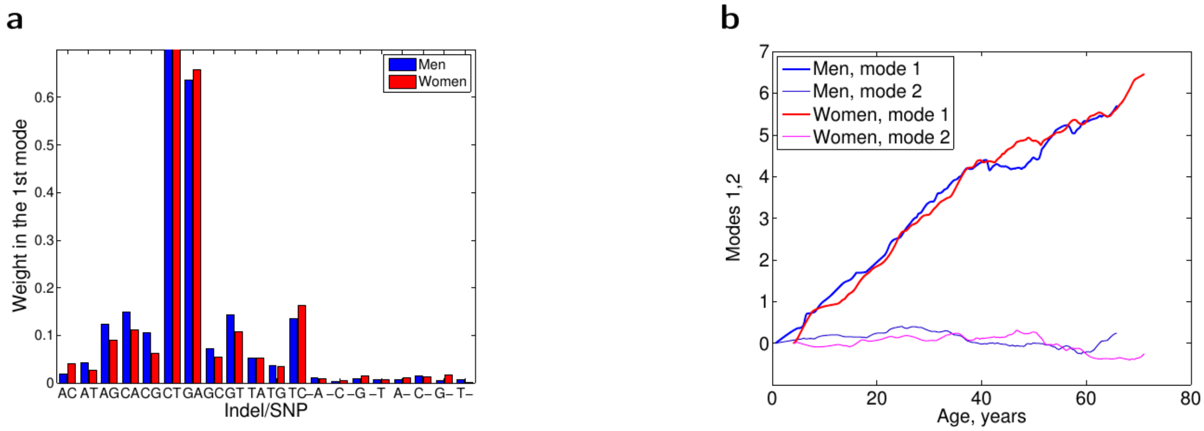

Supplementary Figure 57 | Modal structure of somatic mutation load in acute myeloid leukemia (LAML). **a.** WUSM, IlluminaGA DNASeq; relative weights of different SNVs and indels. **b.** WUSM, IlluminaGA DNASeq; count estimation using projection on the leading pattern.

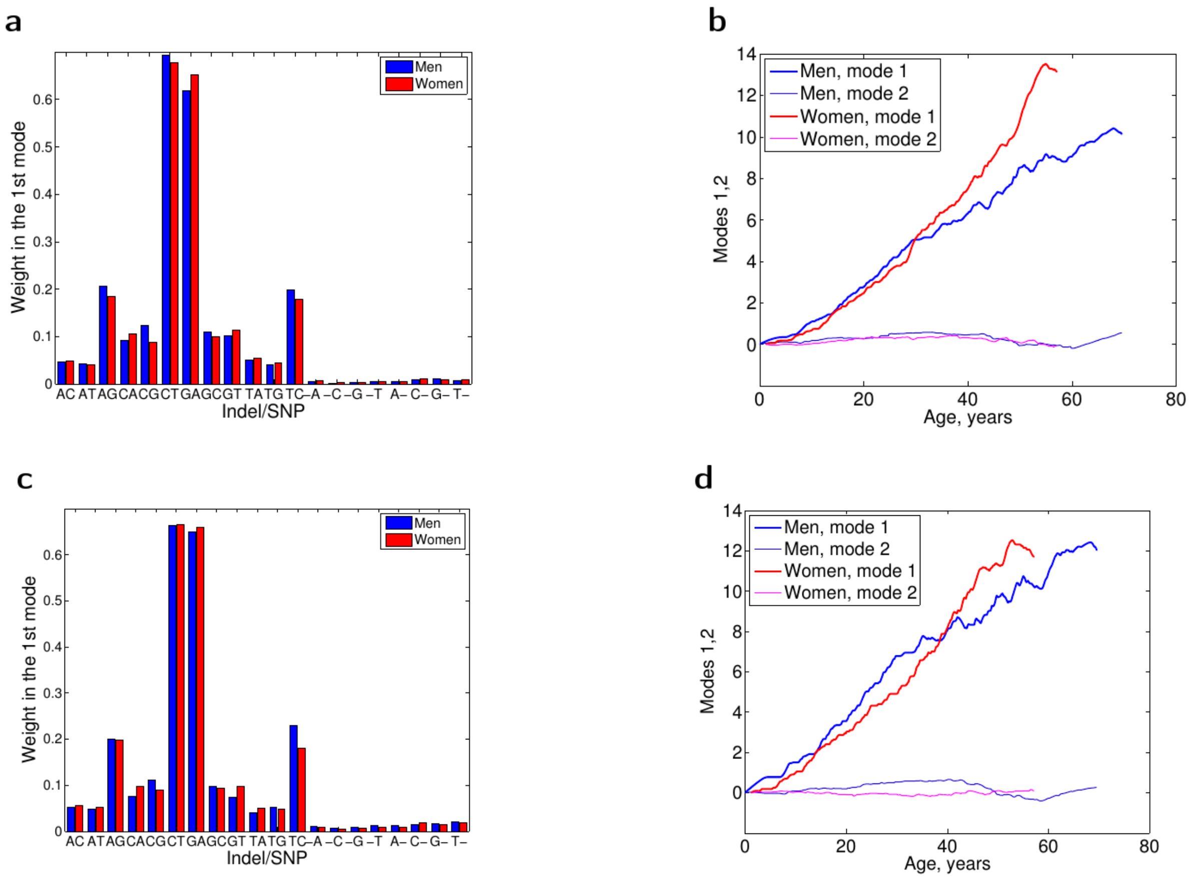

Supplementary Figure 58 | Modal structure of somatic mutation load in brain lower grade glioma (LGG). **a.** BCM, IlluminaGA DNASeq, automated pipeline; relative weights of different SNVs and indels. **b.** BCM, IlluminaGA DNASeq, automated pipeline; count estimation using projection on the leading pattern. **c.** BI, IlluminaGA DNASeq; relative weights of different SNVs and indels. **d.** BI, IlluminaGA DNASeq; count estimation using projection on the leading pattern.

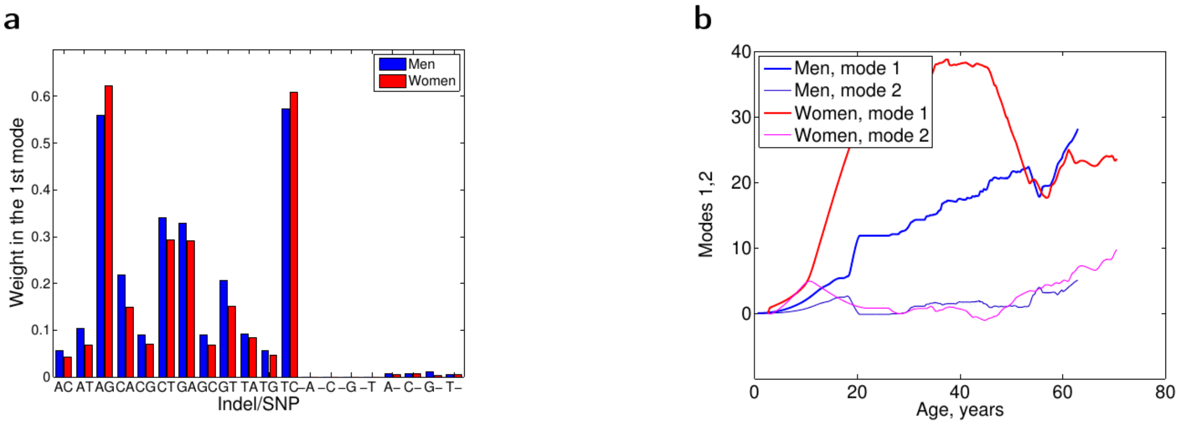

Supplementary Figure 59 | Modal structure of somatic mutation load in liver hepatocellular carcinoma (LIHC). **a.** BCGSC, IlluminaHiSeq DNASeq, automated pipeline; relative weights of different SNVs and indels. **b.** BCGSC, IlluminaHiSeq DNASeq, automated pipeline; count estimation using projection on the leading pattern.

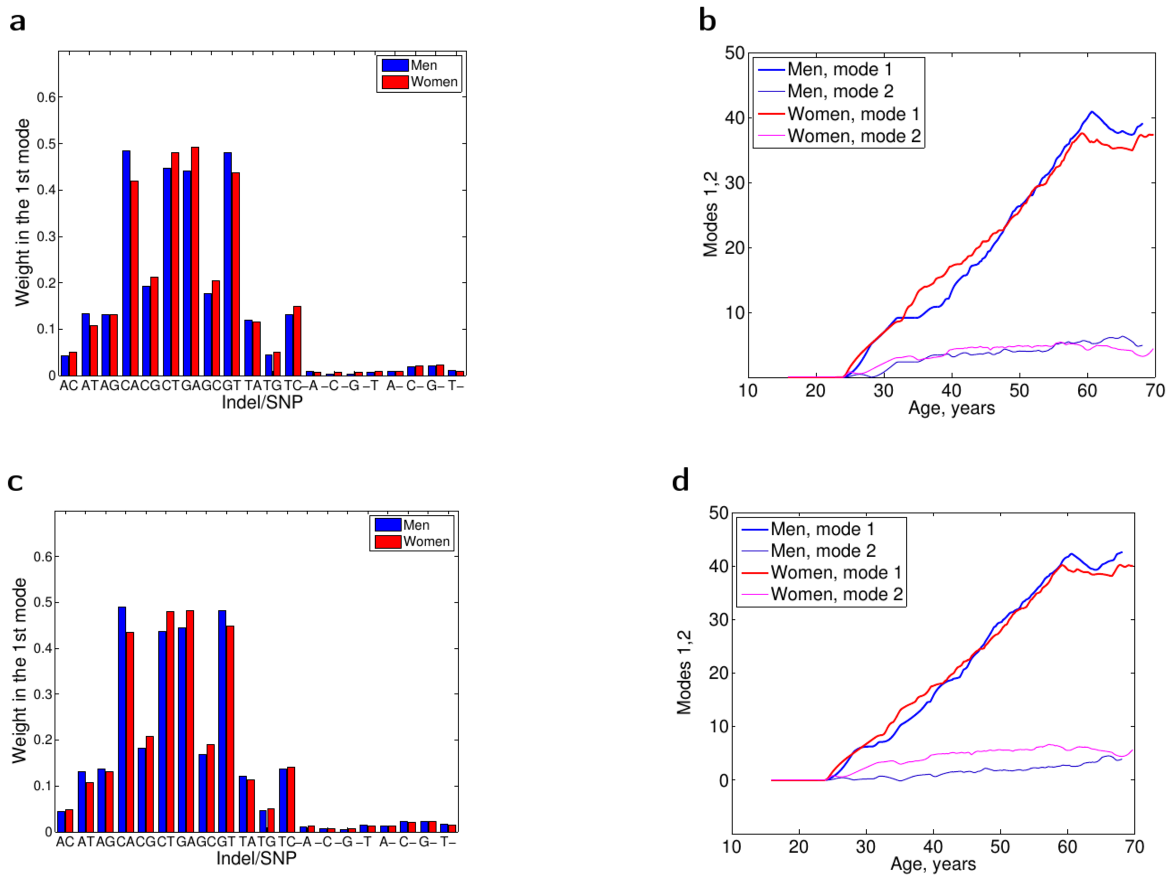

Supplementary Figure 60 | Modal structure of somatic mutation load in lung adenocarcinoma (LUAD). **a.** BI, IlluminaGA DNASeq; relative weights of different SNVs and indels. **b.** BI, IlluminaGA DNASeq; count estimation using projection on the leading pattern. **c.** BI, IlluminaGA DNASeq, automated pipeline; relative weights of different SNVs and indels. **d.** BI, IlluminaGA DNASeq, automated pipeline; count estimation using projection on the leading pattern.

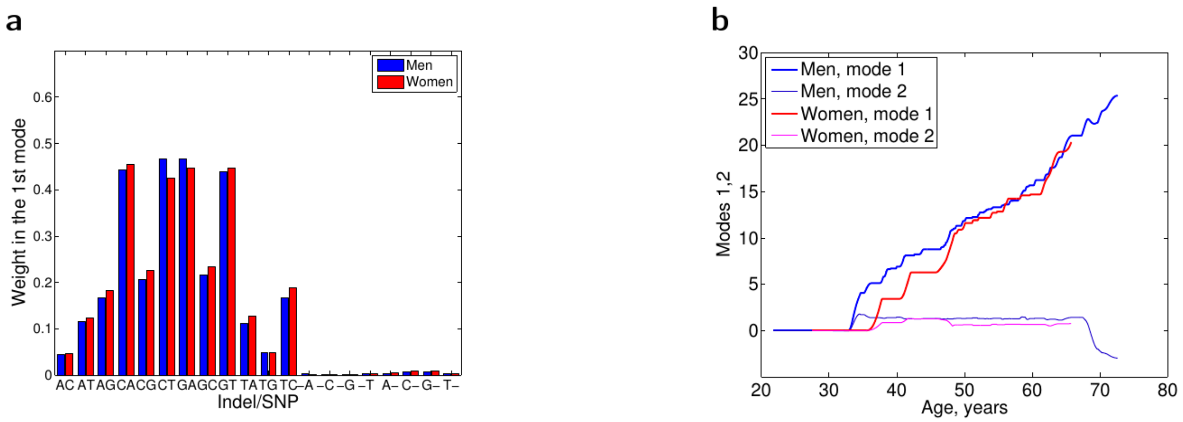

Supplementary Figure 61 | Modal structure of somatic mutation load in lung squamous cell carcinoma (LUSC). **a.** BI, IlluminaGA DNASeq; relative weights of different SNVs and indels. **b.** BI, IlluminaGA DNASeq; count estimation using projection on the leading pattern.

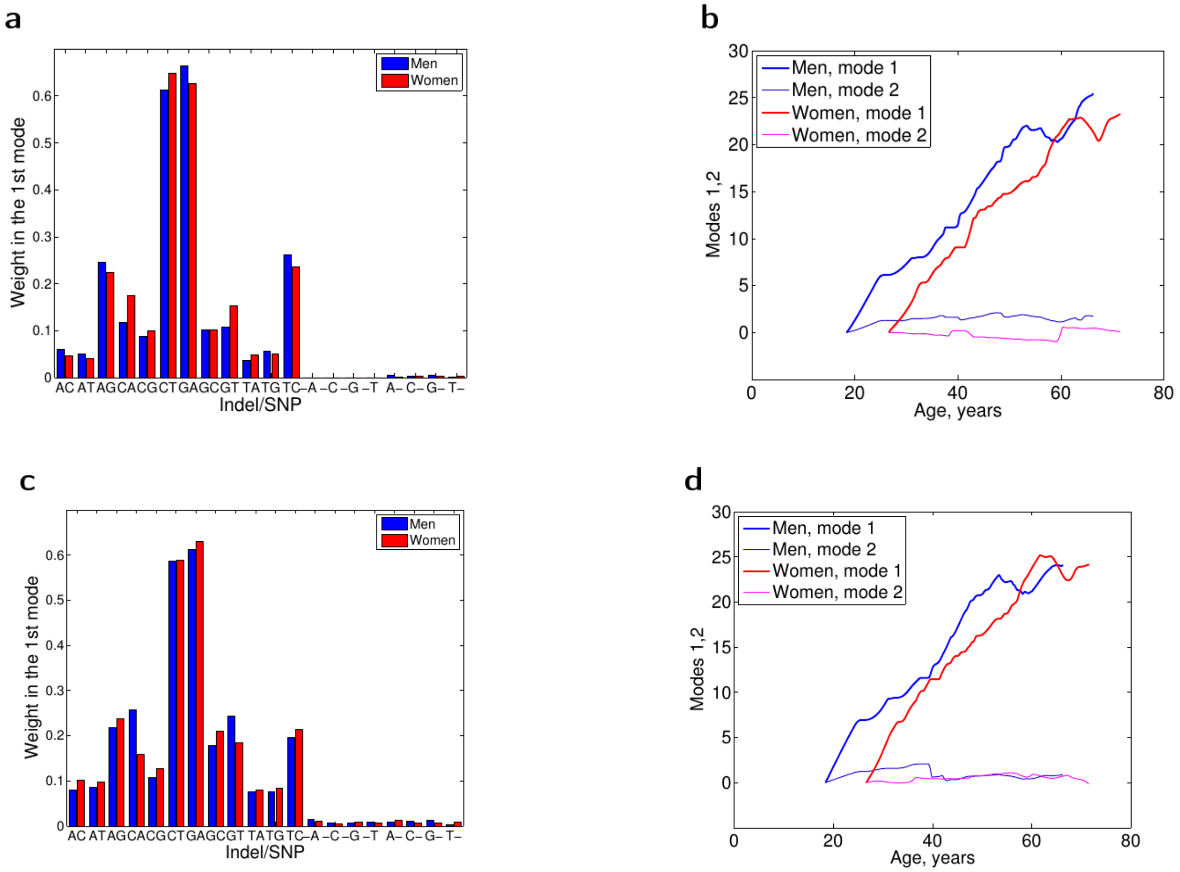

Supplementary Figure 62 | Modal structure of somatic mutation load in lung adenocarcinoma (LUAD). **a.** BCGSC, IlluminaHiSeq DNASeq, automated pipeline; relative weights of different SNVs and indels. **b.** BCGSC, IlluminaHiSeq DNASeq, automated pipeline; count estimation using projection on the leading pattern. **c.** BCM, IlluminaGA DNASeq, automated pipeline; relative weights of different SNVs and indels. **d.** BCM, IlluminaGA DNASeq, automated pipeline; count estimation using projection on the leading pattern.

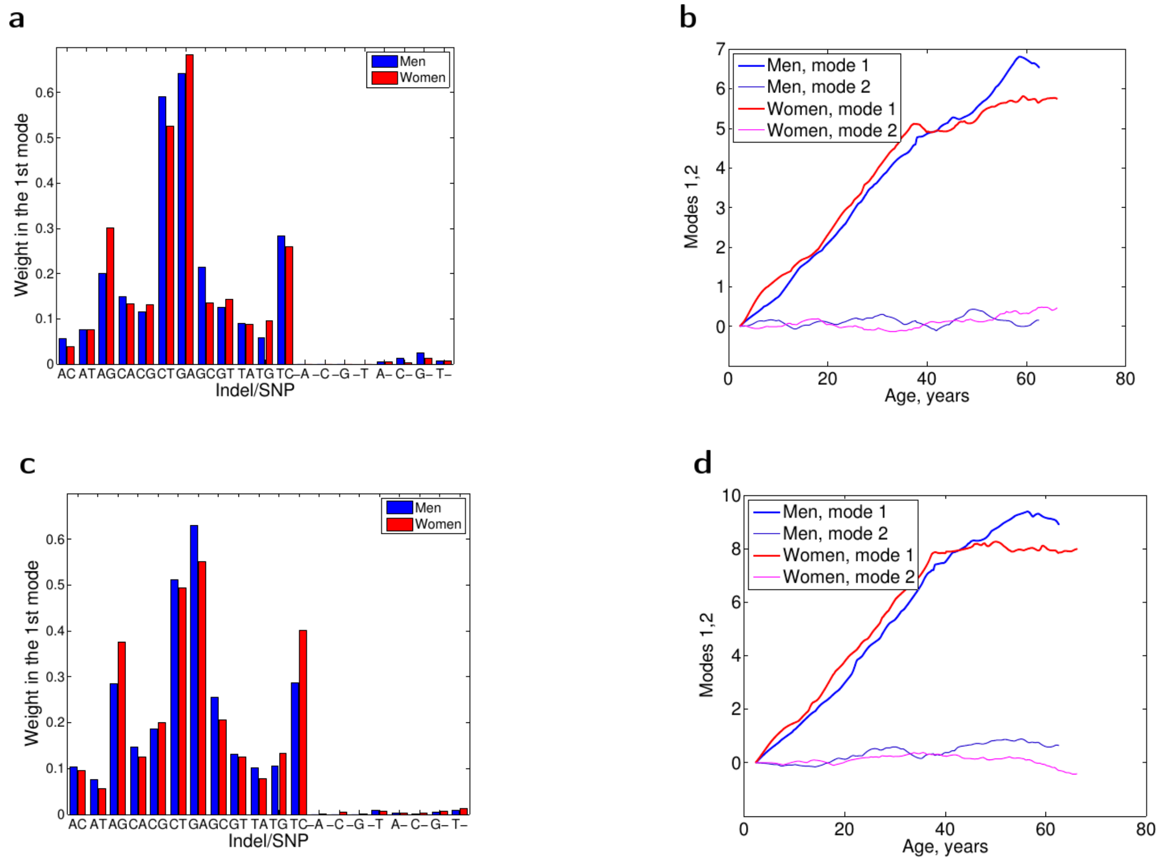

Supplementary Figure 63 | Modal structure of somatic mutation load in pheochromocytoma and paraganglioma (PCPG). **a.** BCGSC, IlluminaHiSeq DNASeq, automated pipeline; relative weights of different SNVs and indels. **b.** BCGSC, IlluminaHiSeq DNASeq, automated pipeline; count estimation using projection on the leading pattern. **c.** BCM, IlluminaGA DNASeq, automated pipeline; relative weights of different SNVs and indels. **d.** BCM, IlluminaGA DNASeq, automated pipeline; count estimation using projection on the leading pattern.

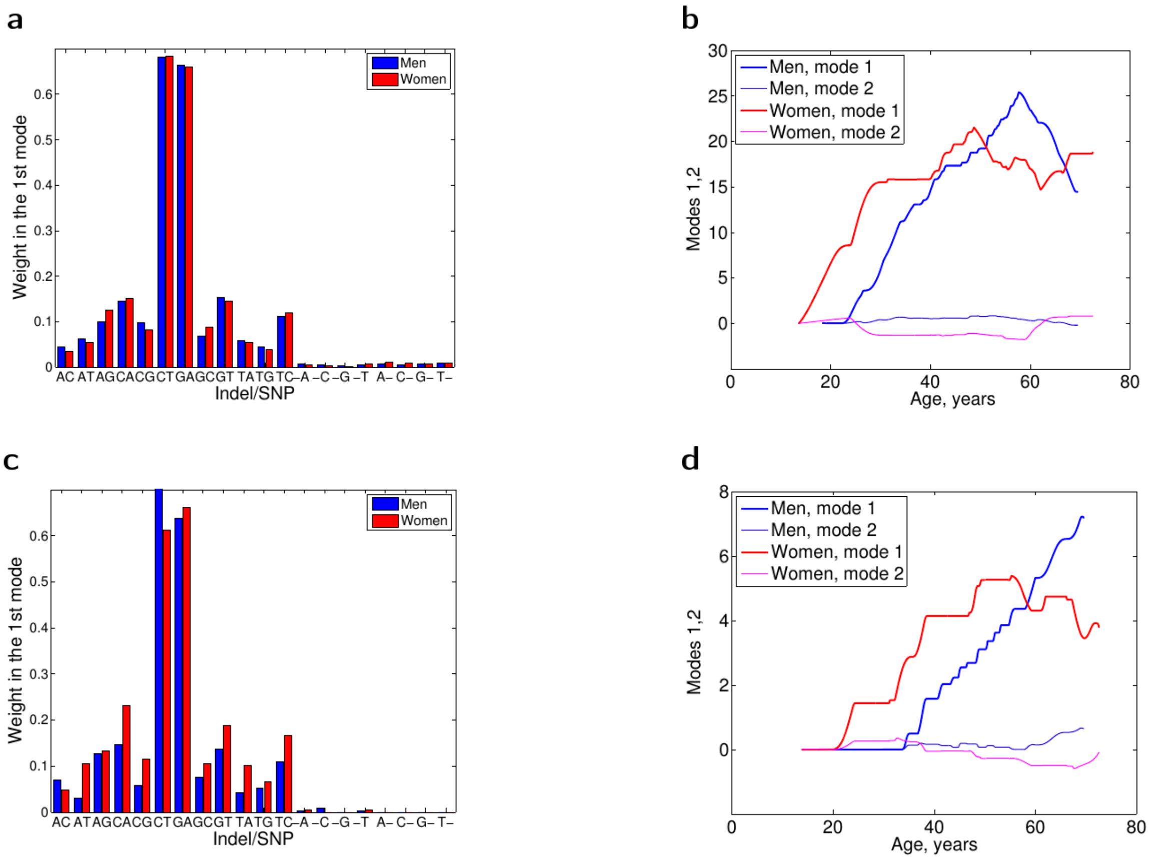

Supplementary Figure 64 | Modal structure of somatic mutation load in rectum adenocarcinoma (READ). **a.** BCM, IlluminaHiSeq DNASeq; relative weights of different SNVs and indels. **b.** BCM, IlluminaHiSeq DNASeq; count estimation using projection on the leading pattern. **c.** BCM, SOLiD DNASeq; relative weights of different SNVs and indels. **d.** BCM, SOLiD DNASeq; count estimation using projection on the leading pattern.

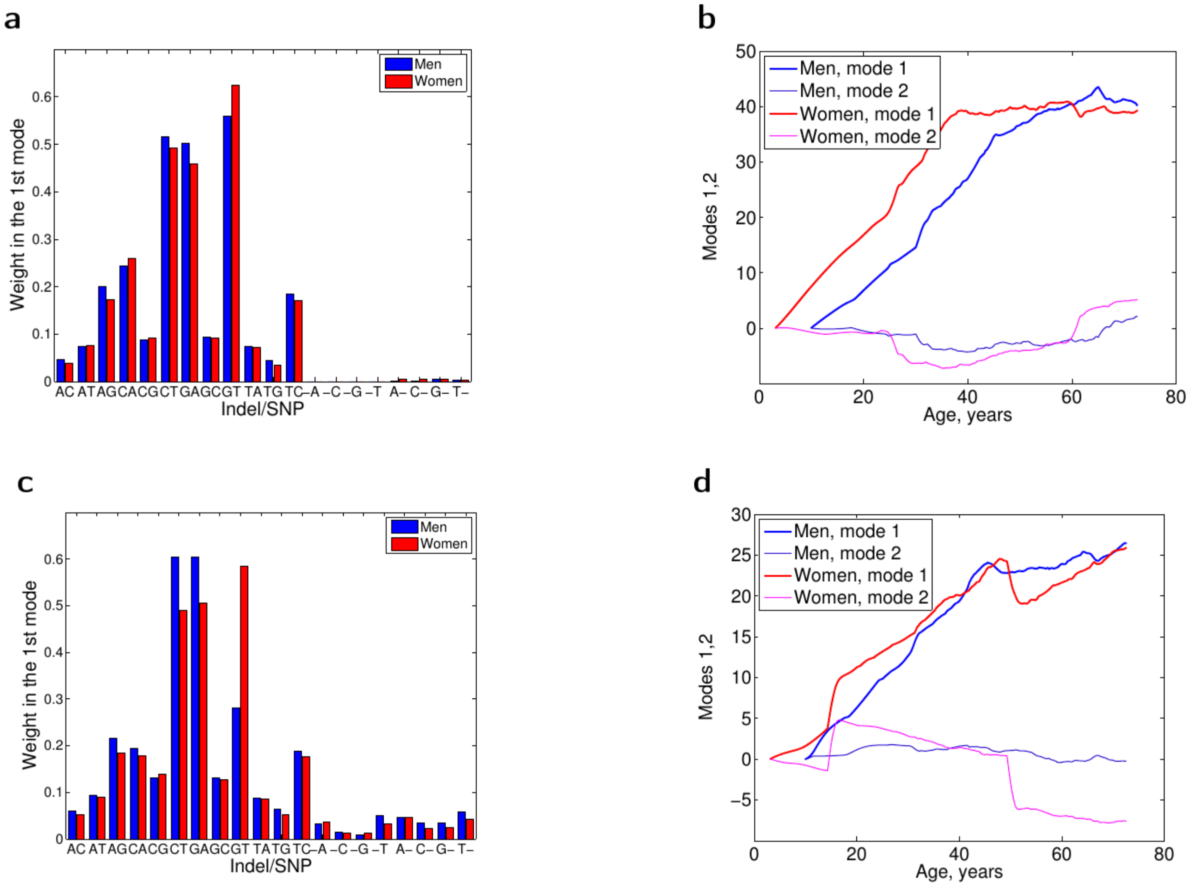

Supplementary Figure 65 | Modal structure of somatic mutation load in sarcoma (SARC). **a.** BCGSC, IlluminaHiSeq DNASeq, automated pipeline; relative weights of different SNVs and indels. **b.** BCGSC, IlluminaHiSeq DNASeq, automated pipeline; count estimation using projection on the leading pattern. **c.** BI, IlluminaGA DNASeq, automated pipeline; relative weights of different SNVs and indels. **d.** BI, IlluminaGA DNASeq, automated pipeline; count estimation using projection on the leading pattern.

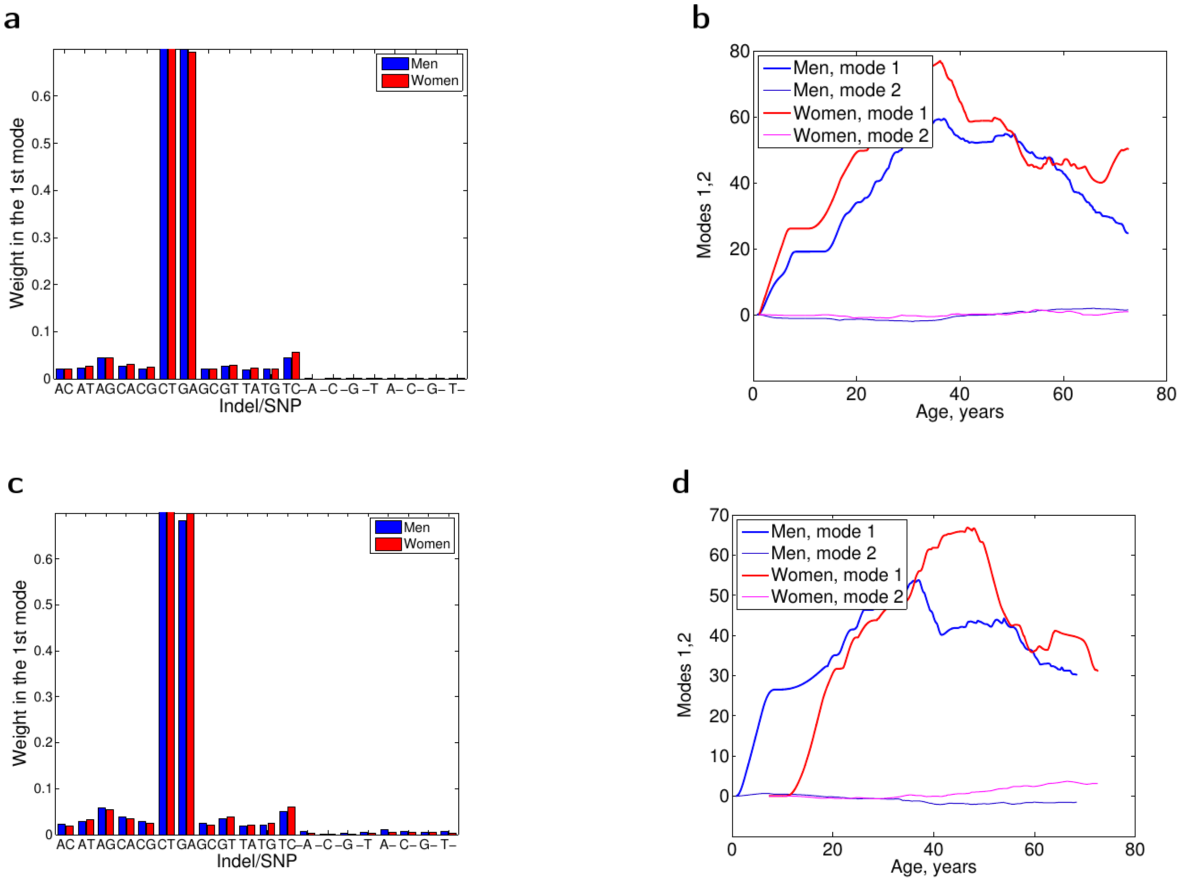

Supplementary Figure 66 | Modal structure of somatic mutation load in skin cutaneous melanoma (SKCM). **a.** BCM, IlluminaGA DNASeq, automated pipeline; relative weights of different SNVs and indels. **b.** BCM, IlluminaGA DNASeq, automated pipeline; count estimation using projection on the leading pattern. **c.** BI, IlluminaGA DNASeq, automated pipeline; relative weights of different SNVs and indels. **d.** BI, IlluminaGA DNASeq, automated pipeline; count estimation using projection on the leading pattern.

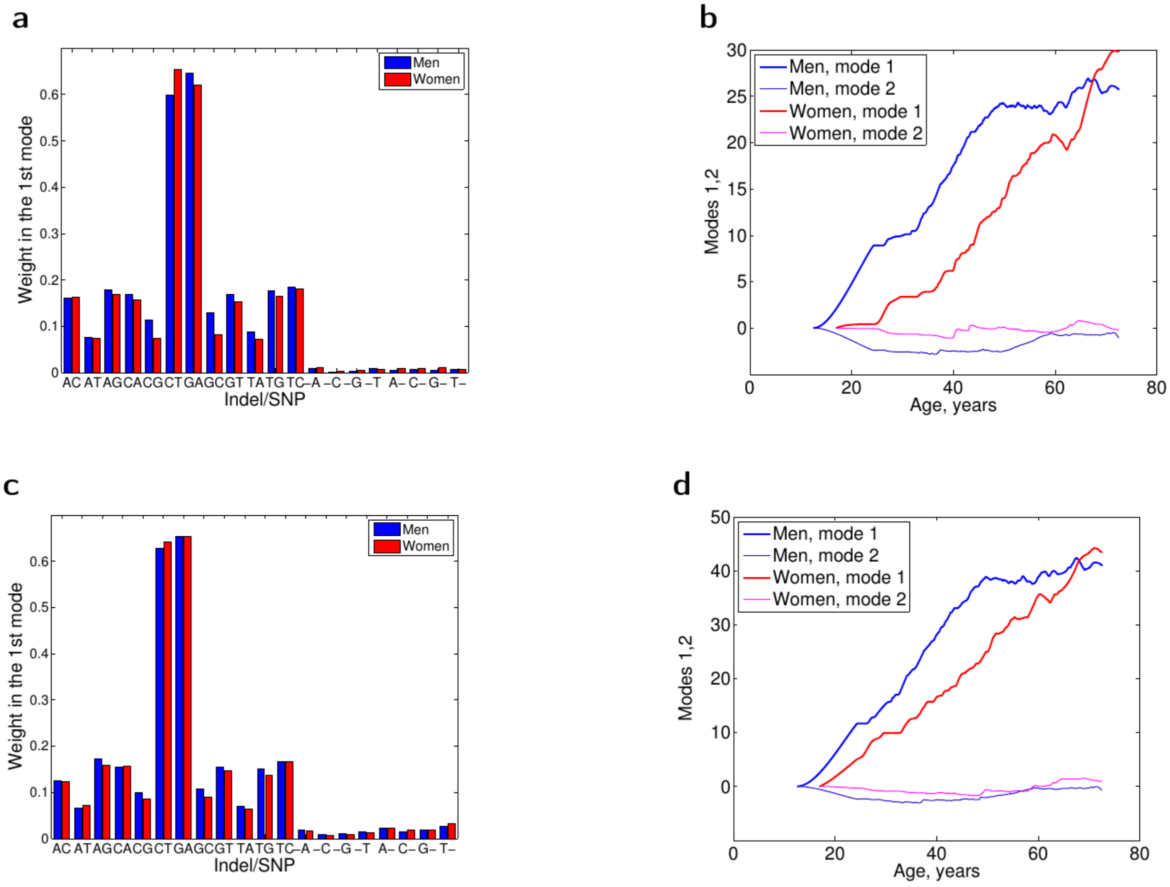

Supplementary Figure 67 | Modal structure of somatic mutation load in stomach adenocarcinoma (STAD). **a.** BCM, IlluminaGA DNASeq, automated pipeline; relative weights of different SNVs and indels. **b.** BCM, IlluminaGA DNASeq, automated pipeline; count estimation using projection on the leading pattern. **c.** BI, IlluminaGA DNASeq, automated pipeline; relative weights of different SNVs and indels. **d.** BI, IlluminaGA DNASeq, automated pipeline; count estimation using projection on the leading pattern.

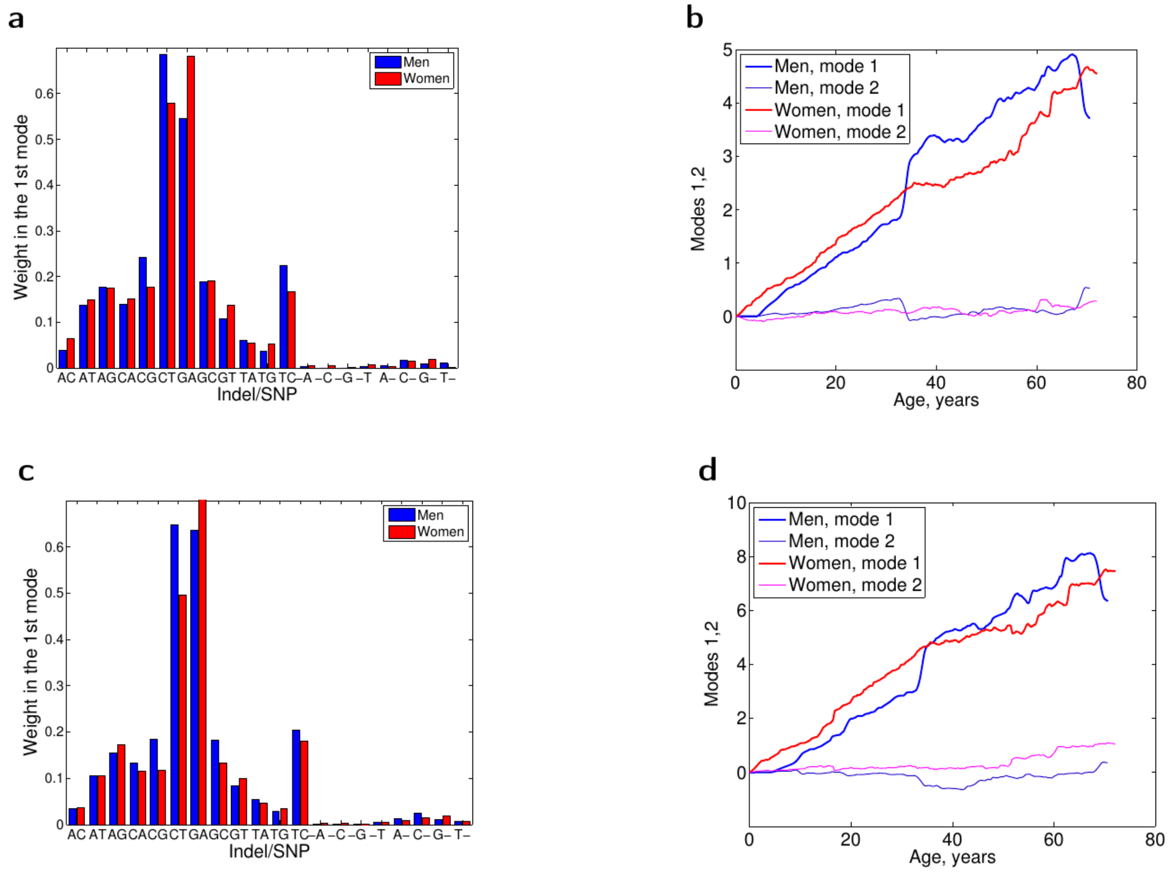

Supplementary Figure 68 | Modal structure of somatic mutation load in thyroid carcinoma (THCA). **a.** BCM, IlluminaGA DNASeq, automated pipeline; relative weights of different SNVs and indels. **b.** BCM, IlluminaGA DNASeq, automated pipeline; count estimation using projection on the leading pattern. **c.** BI, IlluminaGA DNASeq; relative weights of different SNVs and indels. **d.** BI, IlluminaGA DNASeq; count estimation using projection on the leading pattern.

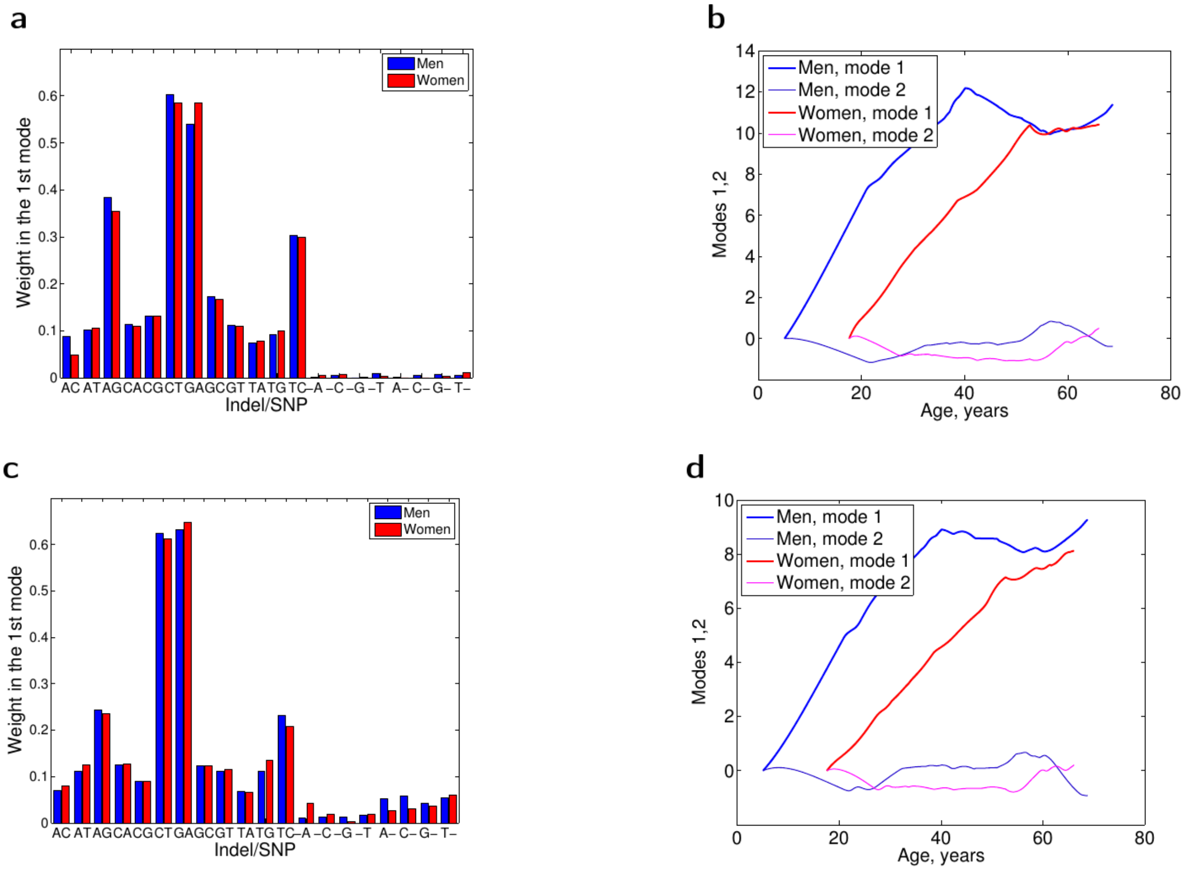

Supplementary Figure 69 | Modal structure of somatic mutation load in uveal melanoma (UVM). **a.** BCM, IlluminaGA DNASeq, automated pipeline; relative weights of different SNVs and indels. **b.** BCM, IlluminaGA DNASeq, automated pipeline; count estimation using projection on the leading pattern. **c.** BI, IlluminaGA DNASeq, automated pipeline; relative weights of different SNVs and indels. **d.** BI, IlluminaGA DNASeq, automated pipeline; count estimation using projection on the leading pattern.

Supplementary Table 1. Somatic mutation accumulation rates in different cancer types (women). Errors are s.d. determined by bootstrapping, see Methods.

Cancer	Datacenter, technology, pipeline	R_{full}, 1/year/exome	R_{silent}, 1/year/exome	α_{full}, 1/year	α_{silent}, 1/year
**ACC**	BCM,IlluminaGA,autom.	1.9429±0.0018	0.7747±0.0004	0.1407±0.0001	0.1457±0.0001
**ACC**	BCM,IlluminaGA,cur.	0.5744±0.0005	0.1536±0.0000	0.1301±0.0001	0.1404±0.0001
**ACC**	BCM,mixed,cur.	3.1138±0.0052	1.1939±0.0010	0.1407±0.0001	0.1466±0.0001
**ACC**	BI,IlluminaGA,cur.	1.2921±0.0009	0.2693±0.0000	0.1323±0.0001	0.1266±0.0001
**BLCA**	BI,IlluminaGA,	1.0181±0.0086	0.4339±0.0018	0.1238±0.0001	0.1680±0.0002
**BLCA**	BI,IlluminaGA,autom.	2.0762±0.0180	0.7617±0.0031	0.1228±0.0000	0.1594±0.0001
**BLCA**	BI,IlluminaGA,cur.	0.9689±0.0051	0.5299±0.0010	0.0860±0.0002	0.0731±0.0000
**BRCA**	WUSM,IlluminaGA	1.1525±0.0003	0.2262±0.0000	0.1341±0.0001	0.1462±0.0001
**BRCA**	WUSM,IlluminaGA,cur.	1.1922±0.0009	0.3339±0.0000	0.1532±0.0001	0.1587±0.0004
**CESC**	BCGSC,IlluminaHiSeq,autom.	2.7436±0.0063	1.3251±0.0038	0.1494±0.0001	0.1929±0.0002
**CESC**	BI,IlluminaGA	0.5505±0.0005	0.1718±0.0001	0.1635±0.0002	0.2284±0.0006
**CESC**	BI,IlluminaGA,autom.	0.5226±0.0004	0.1713±0.0001	0.1698±0.0003	0.2152±0.0005
**CESC**	UCSC,IlluminaGA,autom.	1.9090±0.0028	0.8186±0.0026	0.1802±0.0002	0.2266±0.0002
**CESC**	WUSM,IlluminaGA,cur.	2.4923±0.0031	0.8478±0.0024	0.1516±0.0002	0.2027±0.0002
**CHOL**	BCGSC,IlluminaHiSeq,autom.	0.2945±0.0006	0.0710±0.0000	0.0697±0.0000	0.0701±0.0000
**CHOL**	BCM,IlluminaGA,autom.	0.5789±0.0023	0.1622±0.0002	0.0808±0.0001	0.0997±0.0001
**CHOL**	BI,IlluminaGA,autom.	0.2873±0.0006	0.0562±0.0000	0.0744±0.0000	0.1004±0.0001
**CHOL**	UCSC,IlluminaGA,autom.	0.1604±0.0002	0.0529±0.0000	0.0758±0.0000	0.0944±0.0001
**COAD**	BCM,IlluminaGA	1.5759±0.0053	0.7315±0.0014	0.1408±0.0002	0.1621±0.0003
**COAD**	BCM,SOLiD	0.0637±0.0001	0.1003±0.0001	0.0266±0.0000	0.1225±0.0004
**ESCA**	BCGSC,IlluminaHiSeq,autom.	1.5824±0.0132	0.4244±0.0011	0.1139±0.0001	0.1281±0.0001
**ESCA**	BCM,IlluminaGA,autom.	2.0547±0.0210	0.5533±0.0017	0.1006±0.0000	0.1203±0.0001
**ESCA**	BI,IlluminaGA,autom.	1.4434±0.0117	0.4092±0.0011	0.1138±0.0001	0.1446±0.0001
**ESCA**	UCSC,IlluminaGA,autom.	1.3795±0.0118	0.3217±0.0006	0.1476±0.0001	0.1509±0.0001
**GBM**	BI,IlluminaGA	0.2446±0.0003	0.0554±0.0000	0.0952±0.0001	0.0951±0.0001
**HNSC**	BI,IlluminaGA	0.7703±0.0048	0.2471±0.0005	0.0561±0.0001	0.0705±0.0001
**HNSC**	BI,IlluminaGA,autom.	1.4736±0.0060	0.3949±0.0008	0.1621±0.0003	0.1633±0.0002
**KICH**	BCM,IlluminaGA	0.0677±0.0001	0.0126±0.0000	0.0926±0.0001	0.0972±0.0001
**KICH**	BCM,mixed,cur.	0.5056±0.0030	0.1297±0.0002	0.0849±0.0001	0.0848±0.0001
**KICH**	BI,IlluminaGA,	0.1801±0.0004	0.0343±0.0000	0.0798±0.0001	0.0904±0.0001
**KIRC**	BCM,mixed	0.7688±0.0035	0.1681±0.0002	0.1631±0.0000	0.1981±0.0001
**KIRC**	BI,IlluminaGA,autom.	0.7946±0.0006	0.4386±0.0008	0.1045±0.0001	0.1738±0.0001
**KIRP**	BCM,IlluminaGA,cur.	0.5469±0.0012	0.1296±0.0001	0.1139±0.0000	0.0956±0.0000
**KIRP**	BCM,Mixed,cur.	0.6573±0.0010	0.1569±0.0001	0.1125±0.0000	0.0972±0.0000

**KIRP**	BI,IlluminaGA	0.3288±0.0006	0.0677±0.0000	0.0594±0.0000	0.0447±0.0000
**KIRP**	BI,IlluminaGA,autom.	0.3697±0.0009	0.0653±0.0000	0.0629±0.0000	0.0448±0.0000
**KIRP**	BI,IlluminaGA,cur.	0.5798±0.0012	0.1171±0.0000	0.0837±0.0000	0.0640±0.0000
**KIRP**	UCSC,IlluminaGA,autom.	0.3606±0.0005	0.1557±0.0005	0.0984±0.0000	0.1215±0.0001
**LAML**	WUSM,IlluminaGA	0.2076±0.0001	0.0445±0.0000	0.1244±0.0001	0.1406±0.0003
**LGG**	BCM,IlluminaGA,autom.	0.3689±0.0002	0.1055±0.0000	0.1503±0.0000	0.1600±0.0001
**LGG**	BI,IlluminaGA	0.4227±0.0002	0.9229±0.0411	0.1314±0.0000	0.2523±0.0005
**LGG**	BI,IlluminaGA,autom.	1.0416±0.0012	1.0635±0.0468	0.1648±0.0001	0.2441±0.0003
**LGG**	BI,IlluminaGA,cur.	0.5981±0.0005	0.1339±0.0000	0.1417±0.0000	0.1507±0.0000
**LIHC**	BCGSC,IlluminaHiSeq,autom.	0.7157±0.0034	0.3764±0.0027	0.0790±0.0001	0.0885±0.0003
**LIHC**	BCM,IlluminaGA,autom.	0.3511±0.0013	0.0937±0.0001	0.1157±0.0003	0.1171±0.0003
**LIHC**	BCM,mixed,cur.	0.4919±0.0025	0.1212±0.0002	0.1158±0.0003	0.1137±0.0003
**LIHC**	BI,IlluminaGA,autom.	0.4816±0.0017	0.1624±0.0002	0.1031±0.0001	0.1122±0.0003
**LIHC**	UCSC,IlluminaGA,autom.	0.3653±0.0015	0.0851±0.0001	0.1107±0.0003	0.0972±0.0002
**LUAD**	BI,IlluminaGA	2.9307±0.0059	2.8546±0.0122	0.1305±0.0001	0.1317±0.0002
**LUAD**	BI,IlluminaGA,autom.	3.2698±0.0076	3.1238±0.0106	0.1371±0.0001	0.1392±0.0002
**LUSC**	BI,IlluminaGA	2.2059±0.0084	0.8414±0.0007	0.1243±0.0001	0.0983±0.0000
**OV**	BCM,SOLiD,cur.	0.1896±0.0000	0.0314±0.0000	0.1362±0.0001	0.1418±0.0001
**OV**	BI,IlluminaGA	0.2093±0.0000	0.0453±0.0000	0.1413±0.0003	0.1409±0.0002
**OV**	WUSM,IlluminaGA	0.5382±0.0032	0.1193±0.0002	0.2424±0.0002	0.2343±0.0002
**PAAD**	BCGSC,IlluminaHiSeq,autom.	1.2652±0.0012	2.9848±0.3132	0.1367±0.0001	0.2121±0.0024
**PAAD**	BCM,IlluminaGA,autom.	1.5968±0.0027	0.9587±0.0266	0.1299±0.0001	0.1805±0.0014
**PAAD**	BI,IlluminaGA	0.6053±0.0009	1.1815±0.0455	0.0833±0.0001	0.1629±0.0003
**PAAD**	BI,IlluminaGA,autom.	3.8809±0.0125	1.9321±0.0535	0.1361±0.0001	0.1652±0.0001
**PAAD**	UCSC,IlluminaGA,autom.	0.6379±0.0016	0.3144±0.0104	0.1264±0.0003	0.1583±0.0005
**PCPG**	BCGSC,IlluminaHiSeq,autom.	0.1433±0.0000	0.0368±0.0000	0.1059±0.0000	0.0793±0.0000
**PCPG**	BCM,IlluminaGA,autom.	0.2730±0.0001	0.0434±0.0000	0.1180±0.0001	0.0959±0.0000
**PCPG**	BI,IlluminaGA,autom.	0.2672±0.0001	0.0480±0.0000	0.1081±0.0001	0.0841±0.0000
**PCPG**	UCSC,IlluminaGA,autom.	0.1525±0.0001	0.0460±0.0000	0.1063±0.0000	0.0846±0.0000
**READ**	BCM,IlluminaGA	0.4438±0.0013	0.1396±0.0002	0.1082±0.0001	0.1222±0.0001
**READ**	BCM,SOLiD	0.3904±0.0008	0.0975±0.0001	0.1350±0.0001	0.1235±0.0001
**SARC**	BCGSC,IlluminaHiSeq,autom.	0.8523±0.0019	0.7174±0.0038	0.1208±0.0000	0.2012±0.0002
**SARC**	BI,IlluminaGA,autom.	0.8240±0.0034	0.1366±0.0001	0.1546±0.0001	0.1546±0.0001
**SARC**	UCSC,IlluminaGA,autom.	0.3827±0.0008	0.2152±0.0003	0.1527±0.0000	0.1850±0.0002
**SKCM**	BCM,IlluminaGA,autom.	2.1306±0.0247	2.6886±0.0809	0.1006±0.0000	0.1149±0.0001
**SKCM**	BI,IlluminaGA	2.9425±0.0143	3.9496±0.0519	0.1422±0.0003	0.0790±0.0001
**SKCM**	BI,IlluminaGA,autom.	2.9309±0.0199	4.2844±0.0604	0.1410±0.0004	0.0806±0.0001
**STAD**	BCGSC,IlluminaHiSeq,autom.	0.3740±0.0015	0.0951±0.0002	0.1078±0.0001	0.0991±0.0001
**STAD**	BCM,IlluminaGA,autom.	0.4049±0.0009	0.4030±0.0061	0.1768±0.0003	0.2684±0.0004
**STAD**	BI,IlluminaGA	1.3536±0.0028	1.8059±0.0097	0.1031±0.0000	0.2120±0.0003

**STAD**	BI,IlluminaGA,autom.	1.1559±0.0046	0.6909±0.0147	0.1962±0.0003	0.2686±0.0003
**STAD**	BI,IlluminaGA,cur.	0.5146±0.0015	0.5878±0.0133	0.1643±0.0002	0.2577±0.0004
**THCA**	BCM,IlluminaGA,autom.	0.1994±0.0000	0.0529±0.0000	0.1706±0.0005	0.1503±0.0004
**THCA**	BI,IlluminaGA	0.3211±0.0001	0.0751±0.0000	0.1525±0.0003	0.1436±0.0001
**UCEC**	BI,IlluminaGA	1.0686±0.0008	0.4332±0.0008	0.1576±0.0003	0.1333±0.0002
**UCEC**	WUSM,IlluminaGA	0.6860±0.0003	2.3757±0.0314	0.1573±0.0002	0.0313±0.0000
**UCS**	BCM,IlluminaGA,autom.	1.1407±0.0029	0.3411±0.0003	0.1425±0.0001	0.1578±0.0001
**UCS**	BI,IlluminaGA,autom.	1.7946±0.0066	0.3893±0.0005	0.1420±0.0001	0.1684±0.0001
**UCS**	BI,IlluminaGA,cur.	1.6217±0.0049	0.4064±0.0006	0.1404±0.0001	0.1561±0.0001
**UVM**	BCGSC,IlluminaHiSeq,autom.	0.2281±0.0002	0.3186±0.0022	0.1002±0.0000	0.2006±0.0011
**UVM**	BCM,IlluminaGA,autom.	0.3063±0.0004	0.3470±0.0017	0.0801±0.0000	0.1281±0.0004
**UVM**	BI,IlluminaGA,autom.	0.2329±0.0002	0.3376±0.0026	0.0965±0.0000	0.2171±0.0014
**UVM**	UCSC,IlluminaGA,autom.	0.1496±0.0001	0.2160±0.0010	0.1083±0.0001	0.1827±0.0009

Supplementary Table 2. Somatic mutation accumulation rates in different cancer types (men). Errors are s.d., determined by bootstrapping, see Methods.

Cancer	Datacenter, technology, pipeline	R_{full}, 1/year/exome	R_{silent}, 1/year/exome	α_{full}, 1/year	α_{silent}, 1/year
**ACC**	BCM,IlluminaGA,autom.	0.0945±0.0002	0.2139±0.0013	0.0263±0.0000	0.0763±0.0001
**ACC**	BCM,IlluminaGA,cur.	0.0871±0.0001	0.1668±0.0009	0.0652±0.0001	0.1535±0.0003
**ACC**	BCM,mixed,cur.	0.1717±0.0006	0.3671±0.0036	0.0315±0.0000	0.0898±0.0001
**ACC**	BI,IlluminaGA,cur.	0.0617±0.0001	0.2149±0.0017	0.0363±0.0000	0.1689±0.0006
**BLCA**	BI,IlluminaGA	0.4243±0.0011	0.2031±0.0001	0.1182±0.0001	0.0761±0.0001
**BLCA**	BI,IlluminaGA,autom.	1.6079±0.0135	0.8930±0.0069	0.1619±0.0000	0.2175±0.0001
**BLCA**	BI,IlluminaGA,cur.	0.6858±0.0073	0.5933±0.0067	0.1574±0.0002	0.2329±0.0005
**BRCA**	WUSM,IlluminaGA	0.1861±0.0007	0.0385±0.0000	0.0492±0.0000	0.0465±0.0000
**BRCA**	WUSM,IlluminaGA,cur.	0.2011±0.0004	0.0487±0.0000	0.0561±0.0000	0.0570±0.0000
**CHOL**	BCGSC,IlluminaHiSeq,autom.	1.0173±0.0028	0.1947±0.0001	0.1138±0.0000	0.1076±0.0001
**CHOL**	BCM,IlluminaGA,autom.	1.8091±0.0070	0.3916±0.0003	0.1139±0.0001	0.1164±0.0001
**CHOL**	BI,IlluminaGA,autom.	1.6668±0.0082	0.2457±0.0001	0.1050±0.0000	0.0938±0.0000
**CHOL**	UCSC,IlluminaGA,autom.	0.9276±0.0040	0.1863±0.0001	0.1211±0.0000	0.1185±0.0000
**COAD**	BCM,IlluminaGA	0.8474±0.0021	2.4542±0.0331	0.1071±0.0001	0.0849±0.0001
**COAD**	BCM,SOLiD	0.1058±0.0001	0.0524±0.0001	0.1164±0.0002	0.1395±0.0002
**ESCA**	BCGSC,IlluminaHiSeq,autom.	1.6260±0.0554	0.4185±0.0041	0.1171±0.0001	0.1293±0.0002
**ESCA**	BCM,IlluminaGA,autom.	1.9787±0.0699	0.5349±0.0060	0.1312±0.0001	0.1412±0.0002
**ESCA**	BI,IlluminaGA,autom.	1.5338±0.0504	0.3719±0.0037	0.1194±0.0001	0.1368±0.0002
**ESCA**	UCSC,IlluminaGA,autom.	1.2706±0.0369	0.3056±0.0023	0.1201±0.0001	0.1188±0.0002
**GBM**	BI,IlluminaGA	0.3712±0.0003	0.0743±0.0000	0.1292±0.0002	0.1203±0.0001
**HNSC**	BI,IlluminaGA	0.4938±0.0018	0.1337±0.0004	0.0863±0.0000	0.1034±0.0001
**HNSC**	BI,IlluminaGA,autom.	0.7247±0.0013	0.1910±0.0003	0.1189±0.0001	0.1156±0.0000
**KICH**	BCM,IlluminaGA	0.1618±0.0001	0.0407±0.0000	0.1079±0.0000	0.1117±0.0000
**KICH**	BCM,Mixed,cur.	1.0396±0.0044	0.2616±0.0004	0.1128±0.0000	0.1200±0.0000
**KICH**	BI,IlluminaGA,	0.4409±0.0008	0.0888±0.0000	0.1126±0.0000	0.1214±0.0000
**KIRC**	BCM,mixed	0.7405±0.0030	0.1780±0.0002	0.2325±0.0003	0.2628±0.0005
**KIRC**	BI,IlluminaGA,autom.	0.7046±0.0005	0.2813±0.0005	0.1062±0.0001	0.1207±0.0005
**KIRP**	BCM,IlluminaGA,cur.	0.8734±0.0064	0.2454±0.0005	0.1763±0.0002	0.1673±0.0001
**KIRP**	BCM,mixed,cur.	1.0116±0.0107	0.2501±0.0006	0.1733±0.0001	0.1624±0.0001
**KIRP**	BI,IlluminaGA	0.6483±0.0040	0.1405±0.0002	0.1574±0.0001	0.1376±0.0001
**KIRP**	BI,IlluminaGA,autom.	0.7444±0.0055	0.1590±0.0003	0.1753±0.0002	0.1510±0.0001
**KIRP**	BI,IlluminaGA,cur.	1.1436±0.0132	0.2687±0.0008	0.1984±0.0002	0.1833±0.0002
**KIRP**	UCSC,IlluminaGA,autom.	1.8955±0.0050	1.0576±0.0063	0.1439±0.0003	0.1149±0.0002
**LAML**	WUSM,IlluminaGA	0.1726±0.0001	0.0379±0.0000	0.1586±0.0004	0.1708±0.0005
**LGG**	BCM,IlluminaGA,autom.	0.3318±0.0003	0.0672±0.0000	0.1600±0.0001	0.1609±0.0000
**LGG**	BI,IlluminaGA	0.4224±0.0005	0.0742±0.0000	0.1425±0.0001	0.1754±0.0001

**LGG**	BI,IlluminaGA,autom.	0.9809±0.0019	0.1678±0.0001	0.1904±0.0001	0.1999±0.0002
**LGG**	BI,IlluminaGA,cur.	0.5404±0.0005	0.0810±0.0000	0.1603±0.0001	0.1726±0.0000
**LIHC**	BCGSC,IlluminaHiSeq,autom.	1.3393±0.0114	0.7262±0.0072	0.1735±0.0002	0.0389±0.0001
**LIHC**	BCM,IlluminaGA,autom.	0.4983±0.0005	0.6117±0.0060	0.0699±0.0000	0.0731±0.0005
**LIHC**	BCM,mixed,cur.	1.1337±0.0059	0.4553±0.0072	0.0927±0.0000	0.0540±0.0004
**LIHC**	BI,IlluminaGA,autom.	1.0842±0.0037	0.4638±0.0071	0.1543±0.0003	0.0539±0.0004
**LIHC**	UCSC,IlluminaGA,autom.	0.7949±0.0010	0.3041±0.0059	0.0691±0.0000	0.0432±0.0003
**LUAD**	BI,IlluminaGA	2.7142±0.0098	4.2332±0.0084	0.1412±0.0005	0.1395±0.0001
**LUAD**	BI,IlluminaGA,autom.	3.2160±0.0065	4.1589±0.0110	0.1492±0.0004	0.1385±0.0001
**LUSC**	BI,IlluminaGA	1.3383±0.0026	0.9408±0.0005	0.0748±0.0001	0.0477±0.0000
**PAAD**	BCGSC,IlluminaHiSeq,autom.	0.4994±0.0025	0.1488±0.0004	0.0997±0.0000	0.1115±0.0001
**PAAD**	BCM,IlluminaGA,autom.	0.5629±0.0014	0.0874±0.0000	0.0808±0.0000	0.0891±0.0000
**PAAD**	BI,IlluminaGA	0.6100±0.0038	0.0800±0.0001	0.0996±0.0001	0.0843±0.0001
**PAAD**	BI,IlluminaGA,autom.	1.7058±0.0074	0.2139±0.0001	0.1023±0.0001	0.1127±0.0001
**PAAD**	UCSC,IlluminaGA,autom.	0.1500±0.0002	0.0392±0.0000	0.0695±0.0000	0.0606±0.0000
**PCPG**	BCGSC,IlluminaHiSeq,autom.	0.2388±0.0001	0.0439±0.0000	0.1159±0.0000	0.0861±0.0000
**PCPG**	BCM,IlluminaGA,autom.	0.4127±0.0002	0.0615±0.0000	0.1245±0.0000	0.1055±0.0000
**PCPG**	BI,IlluminaGA,autom.	0.4357±0.0002	0.0696±0.0000	0.1252±0.0000	0.1357±0.0001
**PCPG**	UCSC,IlluminaGA,autom.	0.2286±0.0001	0.0541±0.0000	0.1220±0.0000	0.1196±0.0001
**PRAD**	BCM,IlluminaGA,autom.	0.6015±0.0003	0.1974±0.0000	0.1246±0.0002	0.1172±0.0001
**PRAD**	BI,IlluminaGA,autom.	1.2047±0.0001	0.2725±0.0000	0.1342±0.0002	0.1552±0.0003
**PRAD**	BI,IlluminaGA,cur.	1.1487±0.0001	0.2611±0.0000	0.1316±0.0002	0.1528±0.0003
**READ**	BCM,IlluminaGA	1.8285±0.0023	1.2048±0.0212	0.1197±0.0001	0.0263±0.0000
**READ**	BCM,SOLiD	0.4171±0.0003	N/A	0.1231±0.0001	N/A
**SARC**	BCGSC,IlluminaHiSeq,autom.	0.9468±0.0029	0.2607±0.0003	0.1092±0.0000	0.0943±0.0002
**SARC**	BI,IlluminaGA,autom.	0.9357±0.0016	0.1491±0.0001	0.1018±0.0002	0.1023±0.0002
**SARC**	UCSC,IlluminaGA,autom.	0.4724±0.0008	0.1179±0.0000	0.1043±0.0003	0.1168±0.0002
**SKCM**	BCM,IlluminaGA,autom.	1.7010±0.0162	2.6219±0.0210	0.0838±0.0000	0.1249±0.0001
**SKCM**	BI,IlluminaGA	1.7919±0.0275	3.1573±0.0251	0.0773±0.0000	0.1248±0.0001
**SKCM**	BI,IlluminaGA,autom.	1.0395±0.0138	3.2819±0.0229	0.0533±0.0001	0.1248±0.0001
**STAD**	BCGSC,IlluminaHiSeq,autom.	0.3589±0.0011	0.0857±0.0001	0.0529±0.0001	0.0605±0.0001
**STAD**	BCM,IlluminaGA,autom.	1.4883±0.0086	1.0617±0.0043	0.1370±0.0000	0.1878±0.0005
**STAD**	BI,IlluminaGA	2.0189±0.0093	1.0874±0.0055	0.1357±0.0001	0.1866±0.0006
**STAD**	BI,IlluminaGA,autom.	3.1120±0.0094	1.4803±0.0064	0.1528±0.0001	0.2065±0.0006
**STAD**	BI,IlluminaGA,cur.	1.8112±0.0111	1.2758±0.0063	0.1367±0.0000	0.2036±0.0006
**TGCT**	BCGSC,IlluminaHiSeq_autom.	0.7363±0.0005	1.6580±0.0319	0.1962±0.0003	0.3707±0.0006
**TGCT**	BCM,IlluminaGA,autom.	0.6170±0.0003	0.1448±0.0000	0.1941±0.0003	0.2378±0.0006
**TGCT**	BI,IlluminaGA,autom.	1.2249±0.0013	0.2745±0.0000	0.1645±0.0001	0.1473±0.0002
**TGCT**	UCSC,IlluminaGA,autom.	0.5448±0.0007	0.1850±0.0001	0.2167±0.0003	0.2294±0.0002
**THCA**	BCM,IlluminaGA,autom.	0.1573±0.0000	0.0413±0.0000	0.1217±0.0001	0.1093±0.0000

**THCA**	BI,IlluminaGA	0.2861±0.0001	0.0509±0.0000	0.1597±0.0002	N/A
**UVM**	BCGSC,IlluminaHiSeq_autom.	0.0847±0.0004	0.0135±0.0000	0.0642±0.0002	0.0589±0.0002
**UVM**	BCM,IlluminaGA,autom.	0.1240±0.0007	0.0405±0.0001	0.0599±0.0002	0.0578±0.0001
**UVM**	BI,IlluminaGA,autom.	0.1011±0.0005	0.0113±0.0000	0.0650±0.0002	0.0642±0.0002
**UVM**	UCSC,IlluminaGA,autom.	0.0650±0.0002	0.0146±0.0000	0.0749±0.0002	0.1221±0.0006

Supplementary Table 3. Median chronological ages of screened individuals with samples present in TCGA vs. median chronological ages of cancer incidence for women.

Cancer type	Median age of cancer incidence	Median age of screened individuals (TCGA)
**BLCA**	52.5	73
**BRCA**	46.4	59
**CESC**	32	46
**COAD**	52.5	68
**HNSC**	45	64
**LIHC**	45	63
**OV**	45	59
**PAAD**	50	64
**READ**	54.7	66.5
**SKCM**	39.4	58
**STAD**	52.5	69
**THCA**	29.3	46
**UCEC**	44.8	64
**UCS**	44.8	69
**UVM**	45	60

Supplementary Table 4. Median chronological ages of screened individuals with samples present in TCGA vs. median chronological ages of cancer incidence for men.

Cancer type	Median age of cancer incidence	Median age of screened individuals (TCGA)
**BLCA**	45	68
**BRCA**	46	61
**COAD**	52.5	69
**HNSC**	41	60
**LIHC**	45	60.5
**PAAD**	52.5	67
**PRAD**	45	61
**READ**	51.3	65
**SKCM**	45	57
**STAD**	55	66
**TGCT**	13.4	31
**THCA**	36.4	50.5
**UVM**	42.9	64
